# A 6-gene signature identifies four molecular subgroups of neuroblastoma

**DOI:** 10.1186/1475-2867-11-9

**Published:** 2011-04-14

**Authors:** Frida Abel, Daniel Dalevi, Maria Nethander, Rebecka Jörnsten, Katleen De Preter, Joëlle Vermeulen, Raymond Stallings, Per Kogner, John Maris, Staffan Nilsson

**Affiliations:** 1 Department of Clinical Genetics, Gothenburg University, Gothenburg, Sweden; 2 Department of Mathematical Statistics, Chalmers University of Technology, Gothenburg, Sweden; 3 Genomics Core Facility, Gothenburg University, Gothenburg, Sweden; 4 Center for Medical Genetics, Ghent University Hospital, Ghent, Belgium; 5 Department of Cancer Genetics, Royal College of Surgeons in Ireland and Children's Research Centre, Our Lady's Children's Hospital, Dublin, Ireland; 6 Childhood Cancer Research Unit, Karolinska Institute, Astrid Lindgren Children's Hospital Q6:05, S-171 76 Stockholm, Sweden; 7 Children's Hospital of Philadelphia, Division of Oncology, The University of Pennsylvania, Philadelphia, PA

## Abstract

**Background:**

There are currently three postulated genomic subtypes of the childhood tumour neuroblastoma (NB); Type 1, Type 2A, and Type 2B. The most aggressive forms of NB are characterized by amplification of the oncogene *MYCN *(MNA) and low expression of the favourable marker *NTRK1*. Recently, mutations or high expression of the familial predisposition gene Anaplastic Lymphoma Kinase (*ALK*) was associated to unfavourable biology of sporadic NB. Also, various other genes have been linked to NB pathogenesis.

**Results:**

The present study explores subgroup discrimination by gene expression profiling using three published microarray studies on NB (47 samples). Four distinct clusters were identified by Principal Components Analysis (PCA) in two separate data sets, which could be verified by an unsupervised hierarchical clustering in a third independent data set (101 NB samples) using a set of 74 discriminative genes. The expression signature of six NB-associated genes *ALK*, *BIRC5*, *CCND1*, *MYCN*, *NTRK1*, and *PHOX2B*, significantly discriminated the four clusters (p < 0.05, one-way ANOVA test). PCA clusters p1, p2, and p3 were found to correspond well to the postulated subtypes 1, 2A, and 2B, respectively. Remarkably, a fourth novel cluster was detected in all three independent data sets. This cluster comprised mainly 11q-deleted MNA-negative tumours with low expression of *ALK, BIRC5*, and *PHOX2B*, and was significantly associated with higher tumour stage, poor outcome and poor survival compared to the Type 1-corresponding favourable group (INSS stage 4 and/or dead of disease, p < 0.05, Fisher's exact test).

**Conclusions:**

Based on expression profiling we have identified four molecular subgroups of neuroblastoma, which can be distinguished by a 6-gene signature. The fourth subgroup has not been described elsewhere, and efforts are currently made to further investigate this group's specific characteristics.

## Background

Neuroblastoma (NB) is a childhood tumour of the sympathetic nervous system, and is the most common cancer diagnosed during infancy. The prognosis of NB patients depend upon clinical factors as stage [1], age at diagnosis [2], tumour histopathology [3], and several genetic factors as *MYCN *amplification (MNA) status [4] and DNA index [5]. Generally, children diagnosed before the age of 18 months with a localized tumour have a favourable outcome, whereas older children with metastasised tumours show poor prognosis and low survival rate. However, MNA is proven to be strongly associated with rapid progression and poor prognosis in all patients despite age and stage of disease [4,6] and is therefore central to the risk stratification system in all clinical trial groups [7]. It is still important to emphasize that the majority of metastatic tumours do not show amplification of the *MYCN *oncogene, and other chromosomal aberrations are being evaluated for the International Neuroblastoma Risk Group (INRG) classification system [8].

Studies show that sporadic NB tumours can be assigned to three major subtypes based on their genomic profile, and these molecular signatures also categorize risk groups of NB patients [9]. Type 1 comprise low-risk tumours with triploid DNA content, numeric alterations, and high expression of the nerve growth factor TrkA [10,11], Type 2A involve intermediate-risk tumours with high occurrence of 11q-deletion (del11q) and 17q gain (gain17q) but no MNA, and Type 2B comprise high-risk *MYCN *amplified tumours with high occurrence of gain17q and 1p-deletion (del1p) [12,13]. The outcome prediction of intermediate-risk tumours still remains uncertain and some tumours cannot be definitively assigned to any of the three major groups, indicating this division to be only broadly outlined.

Genome-wide transcriptome microarray analysis enables the possibility to investigate the expression of all genes in a tumour simultaneously. De Preter and colleagues established a 132-gene classifier that discriminates the three major genomic NB subtypes reflecting inherent differences in gene expression between these subtypes [14]. Several studies have established predictive gene signatures of NB tumours [14-20], and others have focused on the differential expression of genes between tumour subsets [21-29]. The three genes *MYCN *[30], *ALK *[31], and *PHOX2B *[32] have been directly linked to NB pathogenesis; *MYCN *is amplified in a subgroup of aggressive metastasizing tumours, activating mutations of *ALK *or amplification is seen in approximately 7% of sporadic cases [33,31-37], and *PHOX2B *is mutated in a subset of familial cases and in a small percentage of sporadic cases [32,38].

In the present study, we explored subtype discoveries by unsupervised expression profiling using Principal Components Analysis (PCA). The analyses identified four distinct PCA clusters in two independent data sets, which were verified in a third larger data set by PCA and unsupervised hierarchical clustering. This study presents a new alternative way of subtype discrimination which will hopefully facilitate the search for subtype-specific therapeutic targets and the development of personalized medicine for children with neuroblastoma.

## Results

### Subtype discovery by PCA

Principal Components Analysis (PCA) was performed on Affymetrix HU133A expression profiles from 17 [22] and 30 [25,39] samples, respectively. Using variance filtering, four distinct clusters appeared (p1-p4, Figure 1). Among the 414 (De Preter data set) and 716 (McArdle/Wilzén data set) genes which defined the clusters in the two test data sets, 226 genes overlapped between the two gene lists (Additional file 1). By cross-validation using a "leaving-one-out" strategy we found our cluster assignments to be relatively stable with only a few exceptions. The De Preter data set showed instability of three samples which shifted cluster belonging in up to 30% of cross-validations. The McArdle/Wilzén data set showed instability of two samples which shifted cluster belonging in one out of 30 cross-validations, respectively. In order to check the robustness of the clustering we made use of the cortex (n = 3) and neuroblast samples (n = 3) from the De Preter data set which were investigated by PCA in relation to the 17 NB samples. As expected, the three cortex samples formed a distinct cluster separated from the neuroblasts and NB tumour samples (Additional file 2).

**Figure 1:**
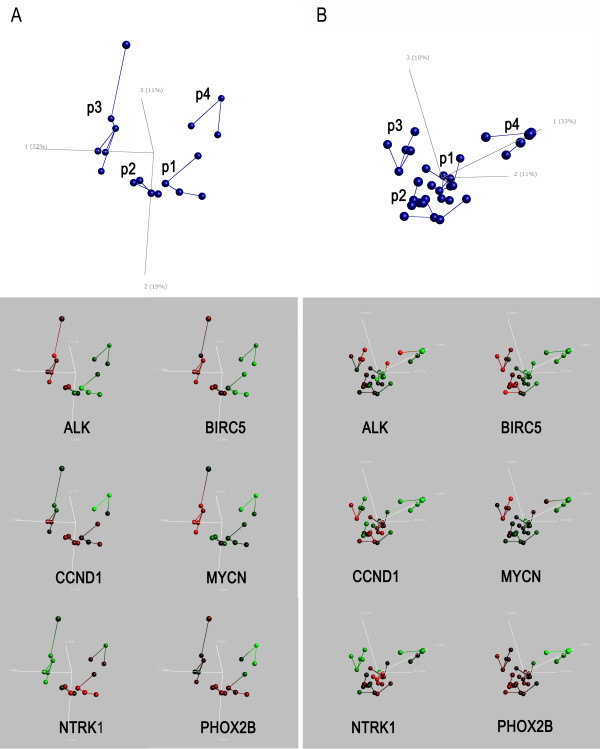
**PCA plots of two parallel cluster analyses**. Principal Components Analysis (PCA) of the two test groups using a variance cut-off of 0.4. **A**. HU133A De Preter data set (414 variables, 17 tumour samples, [22]). **B**. HU133A McArdle/Wilzén data sets (716 variables, 30 tumour samples, [25,59]). Upper panel: PCA clusters, samples (spheres) are joined by nearest Euclidean neighbour. Lower panel: Expression of six NB associated genes Red = High expression; Green = Low expression. The colour scale is based on standard deviations (SD) and ranges from +2 SD (red) to -2 SD (green).

**Figure 2:**
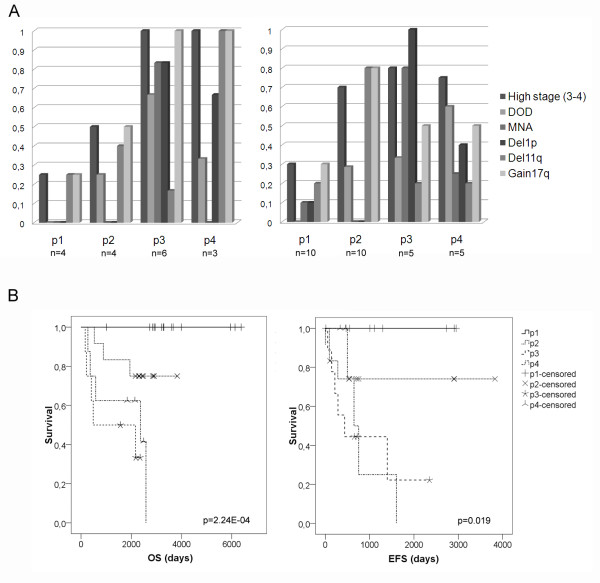
**Frequency of prognostic factors and survival probability in PCA clusters**. **A**. The frequency of prognostic factors in the four PCA clusters p1-p4 for the De Preter data set (n = 17) and McArdle/Wilzén (n = 30). High stage (3-4) = INSS stage 3 and 4; DOD = Dead of disease; MNA = MYCN amplification; Del = deletion. Significant occurrence of prognostic factors in one PCA cluster compared to the others is marked with stars (Fisher's exact test). n = the number of samples in each subgroup. **B**. Kaplan Meier survival curves of PCA clusters (p1-p4) from three data sets (De Preter, McArdle, and Wilzén). OS = Overall survival (n = 43). EFS = Event-free survival (n = 35). Chi-square significance by Log-rank (Mantel-Cox).

By Fisher's exact test, MNA and del1p were found to be significantly more frequent in PCA cluster p3 (p = 0.018 and p = 3.9E-04, Fisher's exact test, table 1, Figure 2A). High stage (INSS stage 3-4), poor outcome, and gain17q were observed in higher frequencies in PCA clusters p2, p3 and p4 (Figure 2A) compared to cluster p1. The frequency of del11q was considerably higher in PCA clusters p2 (data set 2 p = 0,012, Fisher's exact test, table 1) and p4 (p = 0.05, Fisher's exact test, data set 1, table 1). Nineteen out of 47 tumours from both data sets showed del11q, and among those 14 were found in PCA clusters p2 and p4 (Figure 2A).

**Table 1:** Characteristics of the four PCA clusters(p1-p4)

Fisher's exact test	p1		p2		p3		p4			
						
	Data set 1 (n = 4)	Data set 2 (n = 10)	Data set 1 (n = 4)	Data set 2 (n = 10)	Data set 1 (n = 6)	Data set 2 (n = 5)	Data set 1 (n = 3)	Data set 2 (n = 5)		
		
High stage (3 or 4)	0,053*	0,056	0,330	0,691	0,075	0,355	0,324	1,000		
Stage 4	0,029*	0,001**	0,241	0,062	0,088	0,304	0,124	0,696		
Outcome	0,139	0,051*	0,555	1,000	0,072	1,000	0,728	0,070		
MNA	0,208	0,375	0,208	0,141	0,001**	0,003**	0,324	1,000		
Del1p	0,088	0,210	0,088	0,067	0,018*	3,9E-04**	0,360	0,589		
Del11q	0,441	0,235	0,559	0,013*	0,160	0,622	0,051*	0,622		
Gain17q	0,063	0,128	0,365	0,109	0,058	1,000	0,458	0,651		
		
										
**ANOVA one-way**	**ANOVA significance**		**FC p1**		**FC p2**		**FC p3**		**FC p4**	
					
	**Data set 1**	**Data set 2**	**Data set: 1**	**2**	**Data set: 1**	**2**	**Data set: 1**	**2**	**Data set: 1**	**2**

*ALK*	1,1E-03**	0,277	-1,8	-0,5	1,2	0,0	3,6	1,4	-1,0	-0,6
*BIRC5*	6,1E-06**	1,4E-05**	-2,9	-1,7	2,9	2,0	7,8	2,1	-3,0	-2,6
*CCND1*	0,011*	0,003**	0,5	1,1	2,0	0,4	1,0	0,1	-1,9	-2,6
*MYCN*	2,0E-04**	7,0E-04**	-0,6	-0,3	0,4	-0,4	12,6	2,4	-3,5	-1,4
*NTRK1*	6,7E-04**	1,1E-05**	3,7	3,4	3,6	0,7	0,1	-4,0	-0,3	-2,6
*PHOX2B*	0,006**	3,4E-08**	0,5	0,6	2,0	1,0	1,2	0,7	-2,3	-3,2

Fisher's exact test: Each cluster (p1, p2, p3, or p4) versus the other clusters combined. n = the number of samples in each subgroup. High stage = INSS stage 3 and 4; outcome = dead of disease; MNA = *MYCN *amplification; del = deletion. Significance is marked by stars: * p < 0.05, ** p < 0.01. ANOVA one-way: The differential expressions from six genes in PCA clusters (p1-p4) were tested by an ANOVA one-way test. Significance is marked by stars: * p < 0.05, ** p < 0.01. FC = The log2 fold change of genes in each PCA group compared to the other groups combined.

With the intention to explore the expression of genes that have previously been associated with NB, we performed a mining of gene lists from literature. Starting with 15 gene lists [14-16,18-29], 212 genes were found to be present in at least two of the lists, and among those 157 were present in all three data sets (Additional file 3). A PubMed search was performed on the Gene Symbol in co-occurrence with the terms "neuroblastoma" and "gene expression" (Additional file 4) resulting in 30 genes with hits in PubMed. Out of these 30 genes, six NB-associated genes were selected; i) the pre-disposition genes *ALK *and *PHOX2B*, ii) *MYCN *and *CCND1*which are amplified in 20-35% and 3-6% of sporadic neuroblastomas respectively [13,30,40], and iii) *NTRK1 *and *BIRC5 *which have been found to be differentially expressed between subsets of NB [41,42]. All six NB-associated genes were found to be differentially expressed between PCA clusters (Figure 1), which was statistically confirmed by a one-way ANOVA (table 1) and a multiple comparison post-hoc test (Additional file 5). *ALK *and *BIRC5 *were found to be up-regulated in cluster p3 (fold > 2.5) and these genes also showed elevated expression in cluster p2. This was in contrast to clusters p1 and p4 in which *ALK *and *BIRC5 *were found to be down-regulated (table 1, table 2). Also, a 5 times up-regulation of *MYCN *was found in cluster p3 in comparison to clusters p1 or p4. The opposite effect was found for *NTRK1 *which was highly expressed in cluster p1 (10-fold, table 1) and specifically down-regulated in cluster p3 (16-fold, table 1). Cluster p4 consistently showed low expression of all six NB-associated genes compared to the other clusters (table 1, table 2).

**Table 2:** Characteristics of cluster p4

Progn. factor	p4 vs p1			p4 vs p2			p4 vs p3		
High stage (3-4)	0,047988	*	↑	0,48968			0,375645		
Stage 4	0,002127	**	↑	0,583591			0,506192		
DOD	0,009569	**	↑	0,296618			0,600782		
Del1p	0,039341	*	↑	0,009569	*	↑	0,071035		
MNA	0,566667			0,35			0,00905	**	↓
Del11q	0,181631			0,291194			0,165635		
Gain17q	0,212934			0,428148			0,31448		

**Transcript**	**p4 vs p1**			**p4 vs p2**			**p4 vs p3**		

*ALK*	0,696703			0,403066			0,005545	**	↓
*BIRC5*	0,042222	*	↓	1,06E-06	**	↓	4,78E-06	**	↓
*CCND1*	5,43E-05	**	↓	0,000243	**	↓	0,046452	*	↓
*MYCN*	0,036238	*	↓	0,055719	**	↓	0,002016	**	↓
*NTRK1*	0,001017	**	↓	0,03105	*	↓	0,093904		
*PHOX2B*	0,004037	**	↓	0,002773	**	↓	0,006196	**	↓

Progn. Factor: Fischer's exact test of frequency occurrence of prognostic factors in group p4 compared to the other groups separately. High stage = INSS stage 3 and 4; DOD = Dead of disease; Del = deletion; MNA = MYCN amplification. Transcript: T-test by Welch of differential expression in p4 compared to the other groups separately. p-values from each data-set are combined (see text for details). The arrows marks the direction: ↑ Higher frequency or higher expression in p4, ↓ Lower frequency or lower expression in p4.

According to Kaplan-Meier, overall survival (OS) and event free survival (EFS) rates were significantly different between the four clusters (OS p = 2.24E-04, EFS p = 0.019, Log-rank, Mantel-cox). The lowest survival probabilities were found in PCA clusters p3 and p4 with a 5-year OS rate of 50% and 62.5% respectively, and an EFS rate of 22.2% and 25% at 5 year from diagnosis (Figure 2B). In contrast, none out of 14 patients with tumours belonging to PCA cluster p1 died from disease, and the survival probability in this cluster was 100% at 5 years from diagnosis.

### Verification by hierarchical clustering and PCA

In order to verify the existence of the four groups, a discriminative gene set was defined and applied to a third independent data set. First, the p-clusters in data sets 1 and 2 were integrated by reassignment of tumours based on their 6-gene expression profile (r1-r4, Additional file 6). Rules for the r-group assignments were defined by standard deviations (SD) of expression levels and applied to both data sets. The r1-r4 representative of the p-group assignments was found to be very stable (table 3). However, three cases from the De Preter data set and seven cases from the McArdle/Wilzén data set could not be assigned to any r-group based on the rules, resulting in two data sets of 14 and 23 tumour samples, respectively. Next, four Significance Analysis of Microarray (SAM) tests were performed by multiple comparisons on the two data sets separately. The four SAM output gene lists from the two test data sets were compared to generate combined lists of overlapping genes. From the overlapping lists, 30 genes with the highest combined fold change for each of the four contrasts were selected, generating a set of 98 genes. Out of the 98 genes, 74 were present in the third data set (Wang). Third, unsupervised hierarchical clustering and PCA was performed on the Wang data set using the 74 discriminative gene set.

**Table 3:** r-groups vs. p-groups

	**r1**	**r2**	**r3**	**r4**	**ND**
	
**p1**	**9**	1	0	0	4
	
**p2**	1	**10**	0	0	3
	
**p3**	0	0	**10**	0	1
	
**p4**	0	0	0	**6**	2

Contingency table of r-groups and p-groups of the two test data sets (De Preter and McArdle/Wilzén), 47 samples in total. The r-group assignment was based upon MNA status, INSS stage and expression levels of the six NB-associated genes with the highest PubMed search score (see additional file 4). ND = Not determined. The p-group assignment was based upon the PCA clusters (see text for details).

**Figure 3:**
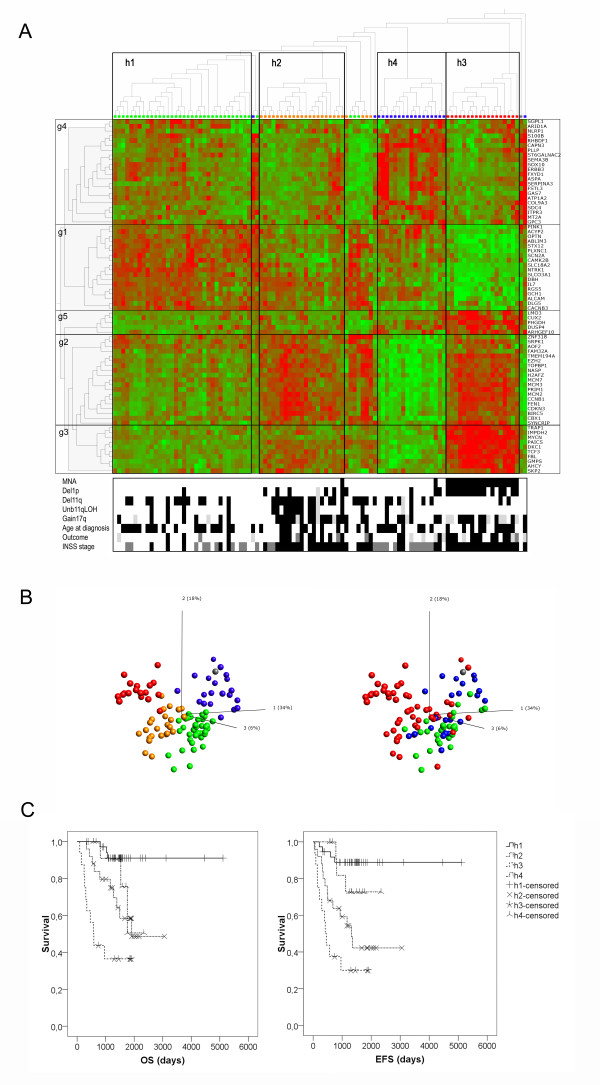
**Hierarchical clustering using a 74 discriminative gene set**. **A**. Unsupervised hierarchal clustering of the Affymetrix HGU95Av2 Wang data set (102 tumour samples in total [28]) by the 74-gene set. The heat map colour scale is based on standard deviations (sd) and ranges from +2 sd (red) to -2 sd (green). The five gene clusters seen are named g1-g5. Status of eight prognostic factors is shown by black and white squares in the lower part: Outcome = Dead of disease; MNA = MYCN amplification; Del = deletion. Black = event, White = No event, Light grey = Not determined. INSS stage is marked as follows: Black = stage 4, dark grey = stage 3. The four hierarchical subgroups seen in the heat map are marked by colour dots: Green = Hierarchical cluster 1 (h1), Orange = h2, Blue = h4, Red = h3, Grey = Human fetal brain. **B**. PCA plot of the 74-classifier gene-set. Left panel: the four hierarchical clusters colour-coded according to the Hierarchical clustering (see above). Right panel: three INSS stages: Green = stage 1, Blue = stage 3, Red = stage 4, Grey = Human fetal brain. **C**. Kaplan Meier survival curves of hierarchical clusters (h1-h4) from the Wang data set. OS = Overall survival (n = 92). EFS = Event-free survival (n = 92). Chi-square significance by Log-rank (Mantel-Cox).

The unsupervised hierarchical clustering of the 101 NB samples clearly divided tumour cases into four distinct subgroups (Figure 3A). Ninety samples were clearly allocated into one of the four hierarchical clusters (h1-h4) based on the dendogram (Figure 3A), and the remaining 11 samples were assigned to a cluster based on the nearest Euclidian neighbour in the PCA (Figure 3B). All the 17 samples assigned to the hierarchical cluster 3 (h3) were stage 4, *MYCN *amplified tumours with high frequency of del1p (Figure 3A, table 4). Samples assigned to hierarchical clusters h2 and h4 consisted of high stage tumours (stage 3-4) with high frequency of del11q without *MYCN *amplification, thus corresponding to the genomic subtype 2A (table 4). Tumours of clusters h2, h3, and h4 all show high content of gain17q.

**Table 4:** Expression subgroups vs. Genomics subgroups

Data sets 1 & 2 (De Preter & McArdle/Wilzén)					
	**p1**	**p2**	**p3**	**p4**	
		
**Type 1**	**10**	4	0	1	
		
**Type 2A**	3	**10**	1	**4**	
		
**Type 2B**	1	0	**9**	1	In total 47
		
**Other**	0	0	1	2	
		
					
**Data set 3 (Wang)**					

	**h1**	**h2**	**h3**	**h4**	
		
**Type 1**	**21**	4	0	**9**	
		
**Type 2A**	15	**15**	0	**7**	
		
**Type 2B**	0	1	**18**	1	In total 101
		
**Other**	2	5	0	3	

Contingency table of p-group assignment and genomic subgroup assignment of the two test data sets (De Preter and McArdle/Wilzén). The p-group assignment was based upon the PCA clusters. The genomic subtypes were established based on INSS stage, MNA status, and Del11q status (see text for details). The highest numbers of cases of the p- and h-group assignments falling into a specific genomic subgroup category are shown in bold.

Patients of cluster h3 showed the worst outcome, with 10 out of 16 dead of disease (2 were lost for follow up) and a survival probability of 36.5% at 5 years from diagnosis (Figure 3C). Patients of cluster h2 also showed a worse outcome, with 10 out of 25 dead of disease and an OS rate of 58.4% at 5 years from diagnosis. In the fourth cluster h4, 3 out of 14 patients died from the disease (6 patients were lost for follow-up), and showed an OS probability of 50.5% at 5 years from diagnosis. The lowest death and relapse rates were seen in hierarchical cluster h1, which showed a 91.1% OS rate and an 88.9% EFS rate at 5 years from diagnosis (Figure 3C). A PCA of the 74 discriminative gene set in the 101 samples shows that the hierarchical subdivision overlaps well with INSS stage subdivision, and that the human fetal brain sample clusters to the hierarchical group h4 (Figure 3B).

Five specific gene clusters (g1-g5) showing differential expression in the four h-groups were noted (Figure 3A). The eighteen genes in cluster g1 mainly involved nervous system maintenance and developmental genes (*e.g. NTRK1*, *DBH*), and those were highly expressed in the favorable h1 group. The h2 and h3 groups showed high expression of a gene cluster encoding cell cycle related proteins (g2). Gene cluster 3 (g3) comprised *MYCN *as well as nine MYCN/c-MYC downstream targets and was highly expressed in the MNA-specific sample group h3. The fourth gene cluster (g4) was specifically over-expressed in tumours of the h4 group and comprised 22 genes which were all found to be involved in nervous system development (*e.g. ERBB3*, *GAS7*, *GPC3*, *SOX10*), nervous system maintenance (*e.g. ATP1A2*, *COL9A3*, *FXYD1*), or associated to CNS in other ways (*e.g. ASPA*, *CAPN3*, *MT2A*, *SERPINA3*, *SGPL1*). The fifth gene set (g5) which was highly expressed in sample group h3, and elevated in sample groups h2 and h4 (Figure 3A) comprised five genes (*ARHGEF10*, *CUX2*, *DUSP4*, *LMO3*, and *PHGDH*) with different functions.

### Validation of PCA clusters and the 6-gene signature

A PCA of unfiltered global transcripts of the three data sets clearly confirmed the existence of four distinct subgroups (Additional file 7). The PCA loadings from data set 2 (McArdle/Wilzén) were utilized to plot the other two data sets (De Preter and Wang). Next, a back-check test was performed by using the PCA loadings from data set 1 (De Preter) to plot the other two data sets (McArdle/Wilzén and Wang).

**Figure 4:**
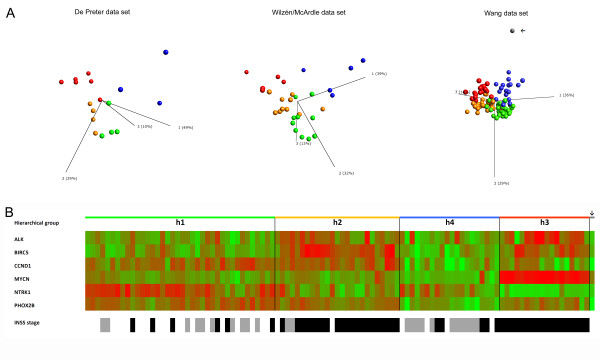
**Verification of 6-gene signature**. **A**. PCA of the six NB associated genes *ALK*, *BIRC5*, *CCND1*, *MYCN*, *NTRK1*, and *PHOX2B *on three data sets: De Preter (left panel), McArdle/Wilzén (middle panel), and Wang (right panel). Samples (spheres) in the two test data sets (De Preter and McArdle/Wilzén) are coloured by the PCA clusters: Green = p1, Orange = p2, Red = p3, and Blue = p4. Samples (spheres) in the Wang data set are coloured by the hierarchical subgroups: Green = h1, Orange = h2, Blue = h4, and Red = h3, Grey = Human fetal brain. Human fetal brain (marked with an arrow) is distinct from the 101 NB samples. **B**. Expression heat map of the 6-gene signature of the Wang data set. The colour scale is based on standard deviations (SD) and ranges from +2 SD (red) to -2 SD (green). Samples are presented in the same order as in Figure 3A.

Also, the 6-gene signature (*ALK*, *BIRC5*, *CCND1*, *MYCN*, *NTRK1*, and *PHOX2B*) was verified to be sufficient for subtype discrimination of both the PCA clusters (p1-p4) identified in data sets 1 and 2 (De Preter and McArdle/Wilzén) and the hierarchical clusters (h1-h4) identified in the Wang data set (Figure 3). Moreover, the 6-gene expression pattern seen in the four hierarchical clusters corresponded well with the expression pattern found in the four initial PCA clusters (Figure 1 and Figure 4B). In addition, the 6-gene set specifically separated the human fetal brain specimen from the other NB tumour samples (Figure 4B), which was in contrast to what could be when seen using the 74-gene set (Figure 3A). A comparison between the expression-based p- and h- group assignments and the genomic-based subtypes showed that the p1 cluster corresponded well to the favourable subtype 1, the p2 to the 2A type, and the p3 to the MNA-specific subtype 2B (table 4). Most tumour cases of the p4 cluster were found among the genomic subtype 2A, although a few p4 cases were also found among other genomic subtypes (table 4). Approximately the same pattern could be seen when comparing the expression-based h1-h3 assignments of data set 3 (Wang) to the genomic subtypes (table 4). However, in the Wang data set a considerable higher number of cases assigned to the fourth cluster h4 were found among the favourable genomic subgroup Type 1 (table 4).

In order to check the previously reported relationship of the *MYCN *and *c-MYC *downstream effects we investigated the expression patterns of the MYC-target genes in the four identified groups (p- and h-groups, Additional file 8). Pearson correlation tests showed *MYCN *and *c-MY*C to be significantly negatively correlated in all three data sets (table 5). *MYCN *was found to be specifically over-expressed in the MNA-specific group p3/h3. In contrast, *c-MYC *over-expression could be seen in a subset of tumours of the p1/h1, p2/h2, and p4/h4 subgroups, but not in any of the tumours assigned to the MNA-specific group p3/h3. The expression pattern of the MYCN/c-MYC downstream targets *AHCY*, *DKC1*, *FBL*, *GMPS*, and *PAICS *were found to preferentially follow the *MYCN *expression levels, and thus most highly expressed in the p3/h3group. Also, a slightly elevated expression of the MYCN/c-MYC downstream targets could be seen in group p2/h2 (Additional file 8).

**Table 5:** Correlations *MYCN *and *c-MYC*

De Preter	Sign.	-,470
	PCC	,057
	N	17
McArdle/Wilzén	Sign.	-,420*
	PCC	,021
	N	30

Wang	Sign.	-,366**
	PCC	,000
	N	101

Pearson Correlations of *MYCN *and *c-MYC *for three data sets. Sign = Significance ** p < 0.01 (2-tailed). * p < 0.05 (2-tailed). PCC = Pearson correlation coefficient. N = number of samples.

## Discussion

A large number of publications prove that cancer can be classified through gene expression profiling. Principal components analysis (PCA) is a useful tool to reduce the dimensions of data to be able to identify and visualize hidden patterns. PCA has been widely used in genome expression studies to discriminate tumour subtypes. For example, Yeoh and colleagues identified prognostically important subtypes and a novel subgroup of pediatric acute lymphoblastic leukaemia (ALL) by PCA of gene expression data [43]. In order to develop more effective and less toxic cancer treatment it is necessary to identify and correctly classify the molecular subtypes, as well as to unravel the underlying oncogenic driving pathways for each type.

In the current study, subtypes of neuroblastoma were explored by expression profiles from four microarray studies [22,25,28,39]. In the first step, PCA was performed on two independent data sets and four distinct clusters were identified in both sets. Prognostic factors such as high INSS stage, MNA, and del1p, differed significantly between clusters. Three of the four clusters (*i.e. *p1-p3) corresponded well to the previously established genomic subtypes 1, 2A, and 2B. Remarkably, a fourth novel cluster (p4) with a considerable different expression profile appeared independently in both data sets, and has not been described elsewhere. This new cluster was found to encompass mainly high stage tumours with poor outcome and high frequency of del11q and del1p, but low frequency of MNA. In data set 1 (De Preter) all 3 cases assigned to cluster p4 were found to be of clinical INSS stage 4, and in data set 2 (McArdle/Wilzén) 3 out of 5 patients died from disease. However, samples assigned to the p4 cluster showed a significantly lower expression of *MYCN *and *ALK *compared to the MNA-specific cluster p3 (table 2), which indicate an alternative progression pathway within tumours assigned to the fourth cluster.

In the second step, the existence of four groups could be verified by an unsupervised hierarchical clustering and PCA of a third data set (Wang data set [28]) using a discriminative gene set of 74 genes from the first step. All tumour samples assigned to the h3 cluster were MNA tumours of clinical stage 4, and tumours assigned to the h2 and h4 clusters comprised high stage tumours with no MNA but with high content of del11q tumours. The lowest survival probability and highest relapse rates were seen in cluster h3, followed by clusters h2 and h4 in descending order (Figure 3C). A comparison between the survival probabilities of the hierarchical and PCA sub-divisions indicated that the h4 cluster constituted less unfavourable tumours compared to those assigned to the p4 cluster. The heterogeneous results seen between data sets may be explained by the small number of cases in data sets 1 and 2 as well as different tumour cohorts. As for example, the Wang data set does not include any clinical stage 2 tumours which might indicate that the cases in this data set were not randomly selected. This would of course affect the subgroup characteristics and might explain the divergent outcome seen in patient cohorts from the different datasets. Also, since the fourth subgroup (p4, h4) is a very small group it is difficult to say if the frequencies of different genetic alterations are stably distributed between the subgroups. Moreover, six patients of the h4 cluster in the Wang data set were lost for follow up, which makes it difficult to draw any major conclusions.

The hierarchical clustering also identified five gene clusters. Nervous system developmental genes, including *NTRK1 *and *DBH *were found to be highly expressed in the favourable tumour cluster h1. Cell-cycle related genes including *BIRC5*, *CCNB1 *and *MCM*-genes were found to be highly expressed in clusters h2 and h3. Not surprisingly, the MYC gene cluster (g3) was specifically found to be over-expressed in the MNA-specific group h3. Westermann and colleagues recently defined a core set of MYCN/c-MYC downstream target genes which were associated with malignant progression in NB [44]. In line with their results, we found *MYCN *and *c-MY*C to be significantly negatively correlated in all three data sets. We also wondered whether the *c-MYC *over-expression could be specifically connected to any of the four groups. However a heat map showed the *c-MYC *over-expression to be evenly distributed among all groups except for the MNA-specific group p3/h3 (Additional file 8). The transcription factor LMO3 (LIM domain only 3) found in gene cluster g5 has been significantly associated with a poor prognosis in NB [45]. This is concordance with the present study in which the highest expression of *LMO3 *was found in the most unfavourable tumour group h3. Interestingly, LMO3 has been shown to interact and act as a co-repressor of p53 [46].

The fourth novel tumour group (h4) was found to be characterized by high expression of several brain-specific and nervous system developmental genes. The Erbb receptors (*e.g. *Erbb3) and the SoxE family (Sox8, Sox9 and Sox10) are essential for development of the sympathetic nervous system and the development of neural crest cells. Interestingly, Leon and colleagues reported that Sox10 and Phox2b act together with the NK2 homeobox Nkx2-1 to modify RET signalling and suggest this interaction to contribute to HSCR (Hirschprungs disease) susceptibility [47]. The growth arrest-specific 7, Gas7, is regulated by Sox9 and the ERK1/2 MAP kinase and is involved in chondrogenesis, and has been reported to form a *MLL/GAS7 *fusion protein in a pediatric case of B-cell acute lymphoblastic leukaemia [48]. In addition, the g4 cluster comprised the suggested 3p tumour suppressor gene *SEMA3B *[49]. The subgroup discrimination properties of *SEMA3B *found in the present study, as well as the significantly lower expression observed in tumour groups p2/h2 and p3/h3, could support its tumour suppressor function.

**Figure 5:**
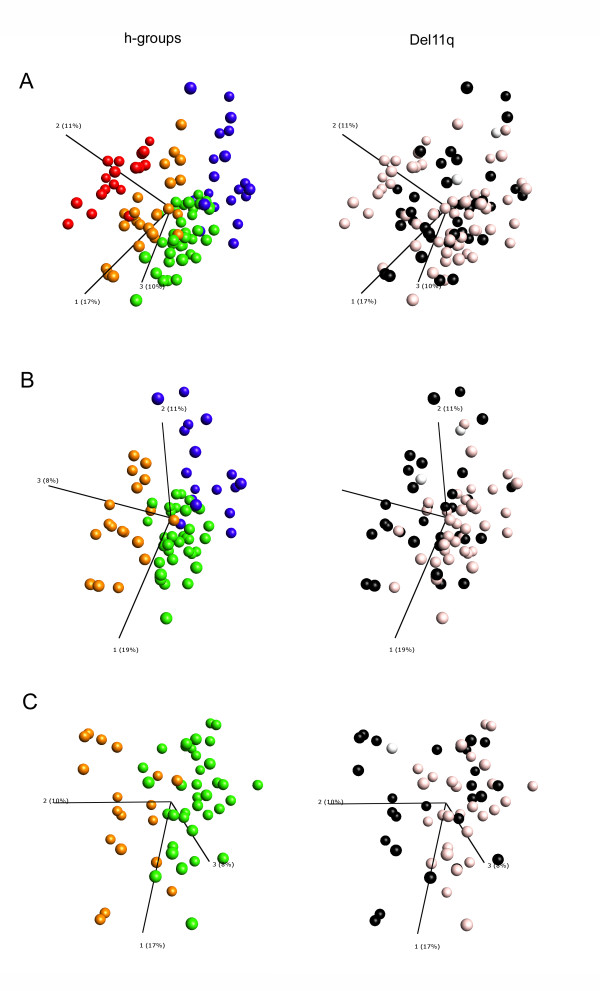
**Del11q groups of the Wang data set**. Unfiltered PCA plots of the Wang data set (101 NB samples, 7542 genes). The four hierarchical clusters (h-groups) in panels A-C (left) are colour-coded as follows: Green = h1, Orange = h2, Red = h3, Blue = h4. Del11q genetic aberrations in panels A-C (right) are colour-coded as follows: Black = 11q-deletion, off-white = No 11q-deletion, White = Undetermined. **A**. PCA of all 101 neuroblastoma cases. **B**. PCA of 74 cases without MNA and Del1p **C**. PCA of 55 cases of the hierarchical groups h1 and h2 without MNA and Del1p.

The validation test of the four PCA clusters using unsupervised and unfiltered global transcripts clearly shows that the four subgroups exist in all three data sets (Additional file 7). Moreover, the PCA of the 6-gene signature in all three data sets convincingly show that this expression profile is sufficient for NB subtype discrimination (Figure 4A). Overall, our results indicate that the most unfavourable group displaying MNA (corresponding to Type 2B) and the most favourable group with high *NTRK1 *expression (corresponding to Type 1) can be easily discriminated by their expression profiles. Moreover, our data indicates that the del11q tumours (corresponding to Type 2A) are divided into at least two expression subgroups (see table 4). Interestingly, the existence of two del11q expression subgroups has recently been reported by two other research groups [23,50]. Fischer and colleagues studied the gene expression patterns of del11q tumours divided into two clinical groups of favourable and unfavourable biology using their previously described prognostic 144-gene expression classifier. They found that the clinical groups clustered using unsupervised PCA and hierarchical clustering [23]. Also, Buckley and colleagues identified a 15-miRNA signature that discriminates two distinct biological subtypes of del11q tumours [50]. Our current study differs from these two studies in one important aspect- it does not rely on any prior subtype division (*e.g. *genomic subtypes, clinical groups *etc.*), which means that it is entirely unbiased. As stated by Fischer and colleagues the 11q-deletion is most likely a secondary event, and it is possible that the decision between favourable and unfavourable neuroblastoma is made by a yet undefined transformation event, for example ALK. This hypothesis is completely consistent with our finding, where *ALK *expression is significantly elevated in subgroup p2/h2 (see Figure 1 and 4B). In order to clarify and relate our subgroup discoveries to these recent findings, we performed a PCA of the Wang data showing tumours marked by their del11q status and coloured by their h-group belongings (Figure 5). Deletion of 11q was found to be distributed through all tumour groups except for the MNA-specific h3-group in which only two cases with 11q-deletion could be found (Figure 5A). In line with Fischer et al. [23] we filtered out all cases with MNA and/or del1p, and ended up with a PCA on 74 cases (Figure 5B). This left us with three expression groups of del11q tumours, one favourable group (h1), and two unfavourable groups (h2 and h4, see Figure 3C). In the last step we removed the h4 expression subgroup, leaving us with two groups of del11q tumours, one favourable (h1) and one unfavourable (h2). These results suggest that our subgroup discoveries are not contradictory to the findings by Fischer et al. [23] and Buckley et al. [50], but rather indicate that there are three del11q expression subgroups instead of two; one favourable with high *NTRK1 *expression (h1), one unfavourable with high *ALK*, *BIRC5 *and *CCND1 *expression (h2), and one smaller group (h4) characterized by high expression of nervous system developmental genes (*e.g. ERBB3*, *SOX10*).

The discriminative power of the six NB genes strengthen the fact that these genes are indeed important in neuroblastoma development. *ALK *was recently recognized as the NB predisposition gene and has thereafter also been found to be affected in sporadic tumours, either though mutations of the tyrosine kinase domain or by genomic amplification [31,34-37]. Interestingly, Passoni and colleagues investigated the *ALK *expression and protein phosphorylation status and found that over-expression of either mutated or wild-type *ALK *defines poor prognosis patients [51]. In this study, we found elevated expression of *ALK *in the p2/h2 group, and the highest expression level was found in the MNA-specific group p3/h3. Moreover, various cell-cycle related genes, including *BIRC5 *and *CCND1*, were found to be highly expressed in sample groups p2/h2 and p3/h3, but low in group p4/h4. The *CCND1 *gene region on 11q has been shown to be amplified in a subset of primary neuroblastic tumours [13,40], and several cases have been found to show an extensive over-expression of cyclin D1 which correlates with histological subgroups [52]. Moreover, *CCND1 *is used as a marker for minimal residual disease of NB [53]. The anti-apoptotic gene *BIRC5 *(also known as survivin) is located in the often gained region on 17q (gain17q), and has previously been found to be associated with poor prognostic factors and low survival probability in NB [41,54]. Recently, Eckerle and colleagues found that *BIRC5 *is a direct transcriptional target of activating E2Fs, and that *BIRC5 *is indirectly induced by N-myc [55]. These findings are supported by the significantly higher expression of both *BIRC5 *and *MYCN *found in tumour group p3/h3 in the current study. Moreover, an elevated expression of *BIRC5 *was found in sample group p2/h2, and a significant down-regulation of both *MYCN *and *BIRC5 *was found in group p4/h4 (p < 0.05, Welch t-test). The MNA-specific p3/h3 group was also characterized by a very low expression of *NTRK1*, whereas the favorable tumour group p1/h1 showed the highest expression of *NTRK1*. TrkA (or NTRK1) is a well-known marker of favorable NB tumours and its expression has been linked to several cancer forms [56].

## Conclusions

In conclusion, by expression profiling of 148 NB tumours from four different Affymetrix-based microarray studies, our data suggest the existence of at least four molecular subgroups of neuroblastoma tumours. Three of the expression-based tumour groups corresponded well to the previously postulated genomic subtypes and a fourth novel group was identified which has not been described elsewhere. The novel tumour group comprised high-stage 11q-deleted tumours with low expression of *ALK *and *MYCN*, but high expression of various CNS and nervous system developmental genes. Our findings suggest an alternative classification system based on expression profiling of a 6-gene signature. Further studies of the novel subgroup's specific characteristics are warranted, and will hopefully lead to discoveries on new specific therapeutic targets for children with neuroblastoma.

## Materials and methods

### Data pre-processing

Raw data files from four published neuroblastoma expression microarray studies generated from two different platforms (*i.e. *three data sets run on the Affymetrix HU133A platform [22,25,39], and one data set generated from the Affymetrix HGU95Av2 platform [28]) were obtained from ArrayExpress http://www.ebi.ac.uk/microarray-as/ae/ and Gene Expression Omnibus http://www.ncbi.nlm.nih.gov/geo/ and reanalyzed. The data were pre-processed in three separate groups; i, Data set 1 (De Preter data set) comprising profiles from 23 NB tumours, preamplified and run on the HU133A platform, ii, data set 2 (McArdle and Wilzén data set) comprising 30 neuroblastic tumours run on the HU133A (not preamplified), iii, data set 3 (Wang data set) comprising 101 NB tumours and one brain tissue sample run on the HGU95Av2. Bioconducter for R 2.9.2 (library BioC 2.4) was used to perform gcRMA normalisation on each data set separately [57]. For each probe-set, the maximum expression value over all samples was determined, and probe-sets which showed very low or no detectable expression levels were filtered out (max log2 expression <6). Next, the mean log2 expression level for each Gene symbol was calculated, resulting in 7439 genes for data set 1, 8106 genes for data set 2, and 7542 genes for data set 3.

### Principal Components Analysis (PCA)

Principal Components Analysis (PCA) was performed using Omics Explorer 2.0 Beta from Qlucore http://www.qlucore.se on data sets 1 and 2 separately. Using the filtering variance slider, genes with the lowest variance were filtered out until a distinct pattern of groups in the PCA plot appeared, resulting in a set of 414 variables for test group 1 and a set of 716 variables for test group 2 (variance cut-off was approximately 0,4 in both data sets). Next, samples were joined to the nearest neighbour using Euclidean distances of all active samples, and clusters of connected samples were defined as separate PCA sample groups. These were cross-validated by a "leaving-one-out" strategy. The eigenvectors of genes included in the variance-filtered PCA for each data set were sorted according to their loadings in Principal component 1, 2, and 3 (PC1, PC2, and PC3) respectively, and gene lists were compared between the two test groups (Additional file 1).

### PubMed gene list

In order to identify known genes which have previously been identified as predictive or differentially expressed in NB disease, the literature was reviewed and gene lists from 15 neuroblastoma expression studies were selected according to the following: 16 differentially expressed genes from Albino et al., 2008 [21], the 55 PCA-based gene module from Asgharzadeh et al., 2006 [15], the 132 PAM classifier gene set from De Preter et al., 2009 [14], 191 differentially expressed genes from De Preter et al., 2006 [22], 220 differentially expressed genes with a fold change above 2 from Fischer et al., 2010 [23], 18 differentially expressed genes from Fischer et al., 2006 [24], the 31 transcripts most strongly associated with the major genetic subtypes of neuroblastoma from McArdle et al., 2004 [25], the 38 top-ranked PAM classifying genes from Oberthuer et al., 2007 [18], the 144 PAM predictor set from Oberthuer et al., 2006 [16], the 41 top-ranked predictive genes from Ohira et al., 2005 [19], the 133 top-ranked genes from Schramm et al., 2005 (from both SAM and PAM analyses) [26], the 89 top-ranked differentially expressed genes from Thorell et al., 2009 [27], 155 differentially expressed genes from Wang et al., 2006 (genes differentially expressed on 1p36 and 11q23, and genes from hierarchical clustering) [28], 72 differentially expressed genes from Warnat et al., 2007 [29], 59 genes selected by data-mining from Vermeulen et al., 2009 [20] (Additional file 4). The intersection of these gene lists resulted in a total number of 1012 unique genes, and among those the genes that occurred in at least two of the 15 gene lists were selected (212 genes, see Additional file 3.). Out of the 212 genes, 157 genes expressed in all three data sets were selected for further co-occurrence search in PubMed. Search terms were the following: "Gene Symbol"[TIAB] AND "Gene expression"[MeSH Terms] AND "neuroblastoma"[MeSH Terms] (search1, Additional file 4), and resulted in hits for 30 genes. In order to get a fair number of PubMed scores we redid the same search including gene alias names for all 30 genes according to Gene cards http://www.genecards.org/ (search 2, Additional file 4). Based on the PubMed results and biological relevance we selected six NB-associated genes from the 30 high score gene list; *ALK*, *BIRC5*, *CCND1*, *MYCN*, *NTRK1 *and *PHOX2B*.

### Statistical analysis and subtype discrimination

The frequency of prognostic marker, *i.e. *INSS stage, outcome, del1p, MNA, del11q, and Gain17q was calculated for each PCA subgroup and tested for significance using Fisher's exact test (table 1). The discriminative power of the six NB-associated genes were tested for significance using a one-way ANOVA test (table 1) and a post hoc (Tukey) test for multiple comparisons. The differential expressions between subgroups were also investigated by Welch t-test (2 sample comparison, unequal variance, table 2). A combined statistic for each gene from the two data sets was constructed as a linear combination of the z-scores (inverse normal distribution of transformed p-values) weighted by the square root of the data sets samples size proportion.

The genomics subtypes were defined based on INSS stage, MNA status, and del11q status (table 4). All tumour cases with MNA were assigned to subtype 2B, all cases displaying del11q with no MNA were assigned to subtype 2A, and all tumours of INSS stage 1, 2, or 3 with no MNA and/or del11q and/or del1p and/or del3p, and which were not dead of disease were assigned to the favourable subtype 1. Tumours that did not fall into any of the categories stated were termed "other" (table 4).

### Verification by hierarchical clustering

In order to identify a subgroup discriminative gene set, the 98 most differentially expressed genes between subgroups were identified by SAM. First, the p- group assignments from the two data sets were translated by reassignment into four integrated groups (r1-r4) defined by rules for expression levels of the six NB genes (Additional file 6). Based on these rules, ten samples (three from the DePreter data set and seven from the McArdle/Wilzén data set) could not be assigned to any r-group, which resulted in two sets of 14 and 23 tumours respectively (Additional file 6). A 2 × 2 contingency table shows the r1-r4 representative of the p1-p4 cluster assignment (table 3). From each independent data set the r1-r4 groups were analyzed by SAM using multiclass comparison (*i.e. *each group was compared to the other groups combined, resulting in four contrasts) [58]. The 4000 most significant genes with a fold change above 2 were selected in each independent data set. Next, SAM gene-lists from the two data sets were compared to create a list of overlapping (or common) genes from each specific contrast. Probe sets with hybridization to more than one gene were filtered out, which resulted in a total of 1987 unique genes overlapping the SAM gene lists from both data sets. For each gene and contrast, the mean log2 fold change from the two data sets was calculated. Next, the genes with the highest combined fold change in each contrast (n = 30) were selected, resulting in a list of 98 unique genes.

The existence of molecular clusters was verified by an unsupervised hierarchical clustering of a third independent data set (Wang data set, comprising 102 samples, [28]). Out of the 98 discriminative genes, 74 genes were present on the Affymetrix HGU95Av2 platform as well as expressed among the 102 samples. Hierarchical clustering of both samples and genes was done using the Average linkage of Euclidian metric (Pearson correlation) for which each variable has been normalized to mean 0 and variance 1. Samples were divided into hierarchical groups based on the dendogram. Samples that allocated in between dendogram trees were assigned to a cluster based on the nearest euclidian neighbour in the PCA-output.

### Validation of PCA

In order to verify that the four identified groups could be recognized and discriminated in all three data sets we performed PCA using the same Principal Components loadings. PCA was performed using the R function prcomp on unfiltered expression data, and PCA plots were visualized in 3D using MatLab R2009a. Prior to the analyses, the three pre-processed data sets were filtered to contain the same set of genes (4728 genes in total) and each gene was normalized to center around zero with unit variance.

In the first test, a PCA was performed using the McArdle/Wilzén data (data set 2) and the loading scores from the first three Principal Components were plotted using different colours for each previously identified group (p1-p4 in data sets 1 and 2, and h1-h4 in data set3, see Additional file 7). Next, the loadings from the McArdleWilzén data set were applied to the De Preter and Wang data sets to examine if the same Principal Components (PC1-3) could discriminate the four groups.

In the second test, we repeated the analysis starting with a PCA on the De Preter data set, and the loadings from the De Preter data were then applied to the McArdle/Wilzén and Wang data sets to check if the same pattern appeared (Additional file 7).

### Survival analyses by Kaplan Meier

The Overall survival (OS) and Event-free survival (EFS) of patients assigned to the four PCA subgroups (p1-p4) from the two test data sets (De Preter, McArdle/Wilzén) were analysed by Kaplan Meier. OS included totally 43 samples and 4 patients were lost for follow up (3 in p2, and 2 in p3). EFS included totally 35 samples and 11 patients were lost for follow up (5 in p1, 3 in p2, 2 in p3, and 2 in p4). Also, OS and EFS analyses of patients assigned to the four hierarchical subgroups (h1-h4) from the Wang data set were analysed by Kaplan Meier. The OS and EFS analyses included totally 92 samples and 9 patients were lost for follow up (1 in h1, 2 in h3, and 6 in h4). The OS significance was calculated by chi-square Log-rank (Mantel-Cox), and the five year survival significance was calculated by Fisher's exact test.

## Competing interests

The authors declare that they have no competing interests.

## Authors' contributions

FA formulated the study design, performed the microarray analysis, PCA, and hierarchical clustering. FA also drafted the manuscript. DD performed programming and cluster calculation, and revised the manuscript. MN verified groups by PCA using unfiltered data. RJ supervised the study design. KD, JV, RS, and JM provided clinical data in terms of status of prognostic marker and survival, and revised the manuscript. SN supervised the study design, statistical analysis, and interpretations of results. All authors read and approved the final manuscript.

## Supplementary Material

Additional file 1 **PCA loadings from the De Preter and McArdle/Wilzén data set**. Column 1-6: Variables (genes/probe-sets) and their PCA loadings for Principal components 1, 2, and 3 (PC1, PC2, PC3) in data-set 1 and 2 (De Preter and McArdle/Wilzén respectively). Common variables: Genes/probe-sets that were present in the PCA analysis of both data-sets. Click here for file

Additional file 2 **PCA of cortex, neuroblast and NB samples**. Unfiltered Principal Components Analysis (PCA) of the De Preter data set (7438 variables, 23 samples. Colour codes of spheres: Red = neuroblastoma tumour specimens; Blue = Neuroblasts; Green = Cortex. Click here for file

Additional file 3 **Workflow of the study**. Step 1: Subtype discovery by unsupervised PCA of two data sets (De Preter and McArdle/Wilzén) from three microarray expression studies (upper panel). Data-mining of gene lists from literature, resulting in the selection of 6 NB-associated genes (lower panel). Step 2: Defining the 74-gene subtype discrimination gene set by SAM (upper panel). Verification of subgroup existence by hierarchical clustering and PCA in a third data set (Wang) using the 74-gene set (lower panel). Click here for file

Additional file 4 **Gene lists from literature & hits in PubMed**. **A**. List of 15 expression studies used for the data-mining. **B**. PubMed searches of 157 and 30 genes respectively. PubMed searches were performed as follows: Search 1(left): 157 genes, search term "Gene Symbol"[TIAB] AND "Gene expression"[MeSH Terms] AND "neuroblastoma"[MeSH Terms]. Search 2 (right):30 genes, search term "Gene Symbol"[TIAB] OR "Alias name"[TIAB]) AND "Gene expression"[MeSH Terms] AND "neuroblastoma"[MeSH Terms]. The six NB-associated genes *ALK*, *BIRC5*, *CCND1*, *MYCN*, *NTRK1*, and *PHOX2B *were selected for further analysis (see text for details). Click here for file

Additional file 5 **Multiple comparisons by Post hoc test (Tukey)**. Gene expression of *ALK*, *BIRC5*, *CCND1*, *MYCN*, *NTRK1*, and *PHOX2B *in PCA clusters p1-p4 of the two data sets De Preter (left) and McArdle/Wilzén (right) was analysed by a Post-hoc test (Tukey). Significance level is marked by a grey colour scale. Click here for file

Additional file 6 **Rules and assignments of r-groups**. Rules for r-group assignments (upper table): Groups (r1-r4) were defined based on the standard deviation (sd) of expression for the six NB-associated genes. R-Assignments of samples from data set 1 and 2 into r-groups (lower table): Expression sd intervals of 5 out of 6 genes had to be in agreement with the rules for each r-group in order to be categorized. Click here for file

Additional file 7 **PCA validation of p- and h-groups using unfiltered expression data**. Principal Components Analysis (PCA) of unfiltered global expression data (4728 genes) from three data sets (De Preter, McArdle/Wilzén, and Wang). **A**. PCA plotted by loadings generated from the McArdle/Wilzén data set. **B**. PCA plotted by loadings generated from the De Preter data set. Cases (spheres) are coloured by their group assignments: Green = p1/h1, Orange = p2/h2, Red = p3/h3, Blue = p4/h4. Click here for file

Additional file 8 **Expression heat map of MYCN, c-MYC and MYCN/c-MYC downstream targets**. The two test data sets De Preter (n = 17, Upper left panel) and McArdle/Wilzén (n = 30, lower left panel) are divided into four PCA clusters (p1-p4), and the verification data set Wang (n = 102, right panel) is divided into four hierarchical clusters (h1-h4). The heat-map colour scale is based on standard deviations (sd) and ranges from +2 sd (red) to -2 sd (green). Status of prognostic factors is shown by black and white squares to the right of each panel. Stage/DOD: Black = INSS stage 4 or dead of disease, Dark grey = INSS stage 3, White = Low INSS stage (stage 1 or 2) and alive, Light grey = Not determined. Click here for file
