# Supplementary material for: A 6-gene signature identifies four molecular subgroups of neuroblastoma
Source: Cancer Cell Int. 2011 Apr 14;11:9. doi: 10.1186/1475-2867-11-9 (PMC3095533; doi:10.1186/1475-2867-11-9)
Supplement: Additional file 1 — PCA loadings from the De Preter and McArdle/Wilzén data set. Column 1-6: Variables (genes/probe-sets) and their PCA loadings for Principal components 1, 2, and 3 (PC1, PC2, PC3) in data-set 1 and 2 (De Preter and McArdle/Wilzén respectively). Common variables: Genes/probe-sets that were present in the PCA analysis of both data-sets. [file 1475-2867-11-9-S1.PDF]

Additional file 1. PCA loadings

De Preter data set					
**Variable**	**PC1**	**Variable**	**PC2**	**Variable**	**PC3**
RRM2	0,1257	SCN3A	0,1288	ALB	0,1700
PRC1	0,1194	RGS7	0,1281	APOH	0,1573
MLF1IP	0,1190	ZFPM2	0,1173	ORM1	0,1542
CCNB1	0,1148	FAM70A	0,1133	LOC100133662 /// RPS4Y1	-0,1519
TYMS	0,1134	SLC18A2	0,1107	FGG	0,1493
CENPF	0,1102	SCG2	0,1097	TF	0,1399
GAL	0,1092	NTRK1	0,1081	HP /// HPR	0,1334
FAM64A	0,1075	RNF11	0,1073	AMBP	0,1316
FOXM1	0,1075	TBC1D9	0,1067	C4BPA	0,1293
CDC20	0,1074	GCH1	0,1016	AHSG /// LOC100131613	0,1212
NUSAP1	0,1073	NT5DC2	0,0979	APCS	0,1209
KIAA0101	0,1041	CRNKL1	0,0962	XIST	0,1191
ASCL1	0,1027	PCDHA1 /// PCDHA10 /// PCDHA11 /// PCDHA12 /// PCDHA13 /// PCDHA2 /// PCDHA3 /// PCDHA4 /// PCDHA5 /// PCDHA6 /// PCDHA7 /// PCDHA8 /// PCDHA9 /// PCDHAC1 /// PCDHAC2	0,0956	FGL1	0,1187
TPX2	0,1024	NOL4	0,0953	ORM1 /// ORM2	0,1187
TOP2A	0,1021	SERPINA3	-0,0949	CYP2C8	0,1136
CKS2	0,1010	FNBP1L	0,0948	FGB	0,1130
DTL	0,1009	PLXNC1	0,0929	HMGCS2	0,1128
BUB1B	0,1001	MFF	0,0927	SAA4	0,1124
DLK1	0,0997	CAMTA1	0,0921	SERPINA3	0,1121
MCM2	0,0996	AMIGO2	0,0920	ARG1	0,1104
PBK	0,0987	MEIS2	0,0910	GC	0,1104
BIRC5	0,0987	ZNF804A	0,0899	HPX	0,1104
PRIM1	0,0987	MEIS1	0,0898	AGT	0,1096
DYNC1I1	-0,0962	AKAP11	0,0895	RBP4	0,1090
CDH19	-0,0955	DOCK4	0,0877	FGA	0,1068
PTTG1	0,0932	PRKCB	0,0872	KNG1	0,1064
CCNB2	0,0927	OPHN1	0,0865	APOC3	0,1063
GINS2	0,0919	SLC39A8	-0,0864	CYP2B6 /// CYP2B7P1	0,1052
MATN2	-0,0918	DDC	0,0856	APOA2	0,1039
ZMAT4	0,0915	GNAI1	0,0854	CP	0,1036
TRIP13	0,0908	SLC18A1	0,0844	APOA1	0,1035
MYCN	0,0897	RGS5	0,0843	SERPINC1	0,1023
SPOCK2	-0,0897	CALB1	0,0841	LBP	0,1019
SV2B	-0,0881	EPHA5	0,0836	CYP2E1	0,1017
MELK	0,0874	FAM69A	0,0834	SPARCL1	-0,1012
MCM6	0,0872	PRKCZ	0,0829	ALDOB	0,1001
UBE2C	0,0870	JUP /// KRT19	0,0829	CRP	0,0976
ASPM	0,0868	HP /// HPR	-0,0825	CACNA2D3	0,0970
POSTN	0,0866	GP1BB	-0,0823	APOB	0,0968
KIF2C	0,0862	APOD	-0,0822	MEIS2	0,0835
ABCA8	-0,0847	FUCA1	0,0817	NCAN	-0,0829
ZWINT	0,0842	CDK5R1	0,0816	PRAME	-0,0821
CUX2	0,0833	NAP1L3	0,0812	IGH@ /// IGHG1 /// IGHG2 /// IGHM /// IGHV4-31	-0,0819
ARHGAP15	-0,0827	MAB21L1	0,0808	MX1	-0,0797
APOD	-0,0817	RABGAP1L	0,0807	DDX3Y	-0,0788
DDX1	0,0802	ZCCHC14	0,0806	STC1	-0,0787
KIF20A	0,0802	ATP6V1G2	0,0804	ZMAT4	-0,0770
SCG2	-0,0796	BAI3	0,0803	P2RX5	-0,0769
KIF15	0,0794	RAMP3	0,0793	GJA1	-0,0756
SST	-0,0792	C4A /// C4B	-0,0785	HOXC4 /// HOXC6	0,0752
MYBL2	0,0792	SV2B	0,0776	ODZ4	-0,0750
MAP1A	-0,0780	NEBL	0,0772	DDC	0,0747
NAV3	-0,0772	FGF13	0,0758	DBH	0,0745
LOC100133662 /// RPS4Y1	-0,0768	NRCAM	0,0757	SIX3	-0,0726
LMO3	0,0758	DNAJC6	0,0757	RGS5	0,0726
CPEB1	-0,0755	PTAFR	-0,0755	FZD2	-0,0717
SEMA3B	-0,0753	FOXC1	-0,0739	EYA1	0,0714
GMNN	0,0753	KIAA1598	0,0739	GSTA1	0,0695
SMC4	0,0752	MRPL48	0,0738	ATF3	-0,0685
TMEM194A	0,0746	ZNHIT3	0,0738	C3	0,0682
DLGAP5	0,0737	CDH18	0,0736	CALCB	-0,0679
KIF4A	0,0723	MIA	-0,0731	TFAP2B	0,0675
NMU	0,0723	GSPT2	0,0730	JUP /// KRT19	0,0668
CEBPD	-0,0721	EGFR	-0,0729	IL7	0,0662
PCNA	0,0709	SST	-0,0717	PHGDH	-0,0659
PTPRH	-0,0702	SERPING1	-0,0708	LMO3	-0,0655
HRASLS3	-0,0702	CDH19	-0,0707	SLCO3A1	0,0644
MIA	-0,0698	CACNA2D3	0,0703	JARID1D	-0,0640
RNASEH2A	0,0696	ALDOC	0,0695	NTRK1	0,0629
JUP /// KRT19	-0,0688	KIAA1107	0,0693	THBS4	-0,0628
CACNA2D3	-0,0686	C3	-0,0690	SERPINF1	-0,0626
CCL2	-0,0685	ZNF91	0,0687	FUT9	-0,0617
ADCY2 /// LOC100133953	-0,0682	DBH	0,0687	MMP12	-0,0614
LGI1	-0,0679	TFAP2B	0,0687	DNAJB1	-0,0608
TWIST1	0,0678	HOXC4 /// HOXC6	0,0685	HBG1 /// HBG2	0,0606
NR4A3	-0,0677	EYA1	0,0685	ZFPM2	0,0599
FZD2	0,0672	ABCA8	-0,0684	TWIST1	-0,0598
COPG2IT1	-0,0672	ORM1	-0,0683	INSM1	0,0597
PHGDH	0,0671	GPR22	0,0677	NHLH2	-0,0595
NTRK1	-0,0670	ST6GALNAC5	0,0668	EIF1AY	-0,0594
S100B	-0,0670	ALB	-0,0662	SERPINE2	0,0589
CRYAB	-0,0667	IL7	0,0659	DLK1	0,0580
ODZ4	0,0660	ELAVL4	0,0658	GABBR1 /// UBD	0,0563
PMP2	-0,0658	ID2 /// ID2B	-0,0657	HOXD11	-0,0558
SLC18A2	-0,0653	MAL	-0,0649	IGH@ /// IGHA1 /// IGHA2 /// IGHV3OR16-13 /// LOC100126583	-0,0558
MGC39900 /// TMSL8	0,0651	ADRB2	0,0649	NEFL	-0,0556
PTN	-0,0644	APOB	-0,0646	SLC18A2	0,0554
MAL	-0,0644	POSTN	0,0646	CALB1	0,0550
NRXN3	-0,0642	CNTNAP2	0,0642	RET	-0,0544
SOX10	-0,0639	TDRD12	0,0630	GCH1	0,0544
PLP1	-0,0635	SOX10	-0,0627	ADAMTS3	-0,0538
P2RX5	0,0629	LYZ	0,0622	HLA-DMA	-0,0538
CRISPLD2	-0,0622	GOLSYN	0,0619	VIP	-0,0536
NEFH	-0,0616	DPP6	0,0615	SV2C	-0,0535
PLAT	-0,0609	PMP2	-0,0615	PRNP	-0,0533
COX7A1	-0,0609	PLP1	-0,0612	ODZ3	0,0532
DET1	0,0609	DET1	0,0609	DDX1	-0,0527
HBB	0,0600	MT2A	-0,0608	CCL18	-0,0515
RELN	-0,0597	APOH	-0,0607	MAL	-0,0502
NR4A2	-0,0596	TST	-0,0607	ELAVL4	0,0501
BAMBI	0,0595	SLCO3A1	0,0601	PLAC8	-0,0501
ASPA	-0,0594	TH	0,0589	MRPL48	-0,0497
MMP9	0,0591	SERPINF1	-0,0588	PTPRH	0,0495
LOC100129762 /// PRUNE2	-0,0588	HLA-DQA1 /// HLA-DQA2	0,0585	EPB41L4B	0,0491
RCAN2	-0,0583	CD53	0,0577	TAGLN	-0,0482

HLA-DMA	-0,0581	LOC100129762 /// PRUNE2	0,0575	TST	0,0472
COBL	-0,0580	EPB41L3	0,0575	CAMTA1	0,0466
HLA-DRA	-0,0574	CD48	0,0574	SELL	-0,0462
FILIP1L	-0,0574	CCL19	0,0573	AKAP7	0,0459
AHNAK2	-0,0572	KIAA0746 /// SERINC2	0,0564	HBB	0,0454
CNTNAP2	-0,0572	MRPL3	0,0558	ASPN	0,0453
ITGA8	-0,0567	SV2C	0,0558	SERPINA5	0,0452
ST6GALNAC2	-0,0561	FGL1	-0,0557	APOD	-0,0450
FAM70A	-0,0560	SRPX	-0,0551	TPX2	-0,0449
SRPX	-0,0560	SEMA3B	-0,0548	FAIM3	-0,0444
RET	0,0559	CALCB	-0,0545	CNTNAP2	0,0443
CALCA	-0,0555	ANGPTL7	-0,0544	NDN	-0,0439
MAB21L1	0,0552	HSPA6	-0,0544	IGK@ /// IGKC	-0,0438
C1S	-0,0551	CYP2E1	-0,0536	HSPA6	-0,0438
CYR61	-0,0550	SNF1LK	0,0535	PTGDS	-0,0437
NT5DC2	0,0536	ASPA	-0,0535	NAV3	0,0433
ECHDC2	-0,0536	DCN	-0,0532	MT1H	0,0433
PTGDS	-0,0533	CRYAB	-0,0531	RRM2	0,0432
INSM1	0,0531	ARG1	-0,0531	SLC18A1	0,0429
ATF3	-0,0530	MGC39900 /// TMSL8	0,0531	HLA-DRA	-0,0427
NPY	-0,0530	PIK3R3	0,0528	ALDH1A2	-0,0425
AMIGO2	-0,0529	GPM6B	-0,0525	IGL@ /// IGLC2 /// IGLV2-14	-0,0418
MRPL3	0,0528	RBP4	-0,0522	DLGAP5	-0,0417
SERPINA3	-0,0525	PRC1	0,0519	MAB21L2	0,0415
CALY	-0,0521	CRP	-0,0516	DET1	-0,0410
PMP22	-0,0520	A2BP1	0,0516	MT1E	0,0408
PDGFRA	0,0516	S100B	-0,0516	C4A /// C4B	0,0404
RGS5	-0,0515	GADD45B	-0,0514	SST	0,0403
MAGEA3	0,0515	FGB	-0,0513	NRCAM	0,0401
XIST	0,0510	FGG	-0,0512	PRKCA	-0,0396
CAMTA1	-0,0505	APOA1	-0,0512	GPM6B	-0,0394
STC1	-0,0504	CALCA	-0,0512	HLA-DQA1 /// HLA-DQA2	-0,0392
GPM6B	-0,0503	PRKCA	0,0506	IFI44L	-0,0392
KIAA1107	-0,0496	TF	-0,0506	ITGB2	-0,0390
PRAME	0,0496	MT1P2	-0,0505	IGHM	-0,0388
NRCAM	-0,0495	THBS4	-0,0505	CALCA	-0,0386
DIRAS3	-0,0492	PTPRH	0,0496	TRBC1 /// TRBC2 /// TRBV19	-0,0385
EGFR	-0,0490	KNG1	-0,0496	C2CD2	-0,0385
MMP12	0,0486	AMBP	-0,0495	SFRP1	0,0381
GADD45B	-0,0485	SERPINE2	0,0493	TCL1A	-0,0381
ADAMTS3	0,0483	RCAN2	0,0492	FAM69A	0,0380
OLFML2A	-0,0477	PHGDH	-0,0489	COX7A1	0,0379
HLA-DPA1	-0,0475	AHSG /// LOC100131613	-0,0486	BAMBI	-0,0379
F12	0,0472	C4BPA	-0,0486	TH	0,0378
MT1E	-0,0472	CFH	-0,0486	ALDOC	-0,0377
MAGEA6	0,0467	PTPRD	0,0483	AMPH	-0,0376
GOLSYN	-0,0461	APOC3	-0,0483	GABRB1	-0,0376
ANGPTL7	-0,0461	ADAMDEC1	0,0482	RGN	0,0372
RALYL	-0,0460	ITGB2	0,0479	PDGFRA	-0,0372
TNS3	-0,0458	SCD5	0,0479	MRPL3	-0,0370
ZCCHC14	0,0456	ASPN	0,0478	MYCN	-0,0369
CGA	-0,0455	SEPP1	0,0477	C1S	0,0368
MT2A	-0,0452	CXCL9	0,0473	CD52	-0,0367
GRP	-0,0452	ALDOB	-0,0472	PLP1	-0,0367
ADAMTS1	-0,0449	PCNA	0,0471	COPG2IT1	0,0358
C4A /// C4B	-0,0448	NHLH2	-0,0470	FOS	-0,0355
SLCO3A1	-0,0446	TG	0,0469	CDH19	-0,0352
MAOB	0,0444	XIST	0,0465	C2orf43	-0,0352
CFH	-0,0443	STC1	-0,0462	GPR22	0,0350
CD74	-0,0443	ST6GALNAC2	-0,0460	FOXC1	-0,0350
PRKCB	-0,0439	ORM1 /// ORM2	-0,0458	BHLHB2	-0,0350
HLA-DPB1	-0,0437	MT1H	-0,0453	GP1BB	0,0349
NEBL	-0,0436	ECHDC2	-0,0450	CPEB1	-0,0346
HOXD11	0,0429	CYP2C8	-0,0446	CUX2	-0,0345
SEPP1	-0,0428	APCS	-0,0443	MT1P2	0,0342
MT1P2	-0,0427	VSNL1	0,0440	ZNF804A	0,0341
GCH1	-0,0427	ASS1	-0,0437	HLA-DPA1	-0,0340
DNAJC6	-0,0425	RELN	-0,0437	SPP1	-0,0338
APOB	0,0425	COPG2IT1	0,0436	LOC100129762 /// PRUNE2	0,0331
SNF1LK	-0,0425	C1S	-0,0431	CD48	-0,0331
TAC1	-0,0420	SAA4	-0,0430	BAG3	-0,0330
EPHA5	-0,0415	MAB21L2	0,0426	PLXNC1	0,0328
ZNHIT3	0,0414	RAB3B	0,0426	A2BP1	0,0327
ALDOC	-0,0412	PDGFRA	-0,0426	CFH	0,0327
SIX3	0,0408	NAV3	0,0422	PRC1	0,0326
DDX3Y	-0,0408	HMGCS2	-0,0421	LGI1	-0,0323
ATP6V1G2	-0,0408	GC	-0,0420	SLC39A8	0,0320
GABRB1	-0,0408	MEST	0,0418	SCN3A	0,0319
GPNMB	-0,0407	FGA	-0,0417	PTPRD	0,0319
C2orf43	0,0405	HPX	-0,0416	SOX10	-0,0311
DPP6	-0,0402	HLA-DRB4	0,0415	ADAMTS1	-0,0307
DNASE1L3	-0,0400	TWIST1	-0,0414	TRBC1	-0,0306
MAGEA4	0,0396	APOA2	-0,0413	DOCK4	0,0304
C7orf16	-0,0393	MT1E	-0,0412	EPHA5	0,0302
HOXC4 /// HOXC6	-0,0389	CYP2B6 /// CYP2B7P1	-0,0411	CCL21	-0,0300
TG	-0,0388	ODZ3	0,0411	DPP6	0,0297
GAGE12F /// GAGE12G /// GAGE12I /// GAGE2A /// GAGE2B /// GAGE2E /// GAGE4 /// GAGE7	0,0388	CP	-0,0410	AHNAK2	-0,0292
BAG3	-0,0383	PRAME	-0,0409	FOSB	-0,0291
RGS7	-0,0381	INSM1	0,0407	EPB41L3	0,0287
RNF11	-0,0379	NMU	-0,0404	OLFML2A	-0,0286
PRNP	-0,0378	HBG1 /// HBG2	0,0403	ASPA	-0,0286
RAB3B	-0,0375	LMO3	-0,0403	AGTR1	0,0285
TH	-0,0368	CEBPD	-0,0401	S100B	-0,0285
NCAN	0,0367	TRBC1 /// TRBC2 /// TRBV19	0,0399	KIAA1598	0,0285
SERPINA5	-0,0366	C2CD2	-0,0395	NRXN3	-0,0281
CTNNAL1	-0,0361	TRBC1	0,0395	SCD5	0,0281
CCL19	-0,0361	FZD2	-0,0395	PMP2	-0,0280
GAGE1 /// GAGE12B /// GAGE12C /// GAGE12D /// GAGE12E /// GAGE12F /// GAGE12G /// GAGE12H /// GAGE12I /// GAGE12J /// GAGE13 /// GAGE2A /// GAGE2B /// GAGE2C /// GAGE2D /// GAGE2E /// GAGE4 /// GAGE5 /// GAGE6 /// GAGE7 /// GAGE8	0,0355	SELL	0,0392	PLAT	-0,0279
KIAA1598	-0,0351	TPX2	0,0389	ECHDC2	0,0279
PPL	-0,0349	LBP	-0,0389	TOP2A	0,0278
MAGEA10	-0,0347	SERPINC1	-0,0386	SNF1LK	-0,0275
SPARCL1	-0,0345	CXCL2	-0,0383	SERPING1	0,0275
CRH	-0,0342	PRIM1	0,0382	ANGPTL7	-0,0274
FOXC1	0,0339	GMNN	0,0376	MAGEA3	0,0274
EYA1	0,0338	HOXD11	-0,0376	CRNKL1	-0,0270
BHLHB2	-0,0335	ODZ4	-0,0375	ARHGAP15	-0,0269

GAGE1 /// GAGE12F /// GAGE12G /// GAGE12I /// GAGE12J /// GAGE2A /// GAGE2B /// GAGE2C /// GAGE2D /// GAGE2E /// GAGE3 /// GAGE4 /// GAGE5 /// GAGE6 /// GAGE7 /// GAGE8	0,0329	DDAH1	0,0373	ARID5B	-0,0265
MT1H	-0,0326	KIAA0101	0,0370	CDK5R1	-0,0264
CXCL2	-0,0326	SIX3	-0,0367	HLA-DPB1	-0,0263
MET	0,0325	PTGDS	-0,0363	GPNMB	-0,0263
GAGE1 /// GAGE12F /// GAGE12G /// GAGE12I /// GAGE12J /// GAGE2A /// GAGE2B /// GAGE2E /// GAGE4 /// GAGE5 /// GAGE6 /// GAGE7	0,0325	DIRAS3	0,0362	NT5DC2	-0,0259
MRPL48	0,0323	MMP12	0,0361	CD74	-0,0259
CRNKL1	0,0320	COX7A1	0,0357	RALYL	-0,0255
CYP11B1	-0,0319	VIP	-0,0351	EGFR	0,0255
GAGE12B /// GAGE12C /// GAGE12D /// GAGE12E /// GAGE12F /// GAGE12G /// GAGE12H /// GAGE12I /// GAGE13 /// GAGE2A /// GAGE2B /// GAGE2C /// GAGE2E /// GAGE4 /// GAGE5 /// GAGE6 /// GAGE7	0,0315	GPNMB	0,0351	GNAI1	0,0253
CCL18	0,0315	DYNC1I1	0,0350	NR4A2	-0,0251
MT1X	-0,0314	DLK1	0,0346	POU2AF1	-0,0250
ZFPM2	-0,0313	NRXN3	-0,0345	IL8	-0,0249
AKAP7	-0,0311	MAGEA3	-0,0344	RNASEH2A	-0,0245
AKR1C2	-0,0306	KCNK3	0,0342	ST6GALNAC2	-0,0243
CDH18	-0,0306	ADCY2 /// LOC100133953	0,0341	FAM70A	0,0242
ASS1	0,0305	MCM6	0,0341	MAP1A	-0,0241
ARID5B	-0,0302	COBL	-0,0340	TDRD12	0,0240
HSD3B2	-0,0302	IL8	-0,0340	CTNNAL1	-0,0239
IL7	-0,0300	AMPH	0,0336	ST6GALNAC5	0,0238
HBA1 /// HBA2	0,0300	PLAC8	0,0334	SLC47A1	0,0236
CYP11A1	-0,0294	P2RX5	-0,0331	AKAP11	-0,0234
CYP21A2	-0,0290	NR4A3	-0,0330	MT1X	0,0233
FUT9	0,0273	ZWINT	0,0330	RAMP3	0,0231
FOSB	-0,0272	ZMAT4	-0,0328	PTAFR	0,0231
STAR	-0,0270	HRASLS3	-0,0327	SRPX	-0,0230
DCN	-0,0268	LGI1	-0,0326	NUSAP1	0,0225
SLC47A1	-0,0260	ARHGAP15	-0,0324	CCL19	0,0218
XAGE1A /// XAGE1B /// XAGE1C /// XAGE1D /// XAGE1E	0,0257	MAOB	-0,0324	GSPT2	-0,0216
FGF13	0,0255	MT1X	-0,0323	EFEMP1	0,0216
JARID1D	-0,0254	NUSAP1	0,0321	MAOB	-0,0214
C7	-0,0253	XAGE1A /// XAGE1B /// XAGE1C /// XAGE1D /// XAGE1E	-0,0320	CEBPD	-0,0213
PTPRD	-0,0250	F12	-0,0317	KIF4A	-0,0211
MX1	-0,0246	ASCL1	-0,0316	S100A8	-0,0210
MAGEA11	0,0242	GJA1	0,0314	TG	0,0206
CXCL9	-0,0241	STAR	-0,0307	MT2A	0,0206
SLC18A1	-0,0239	TAGLN	0,0306	MAGEA4	-0,0204
CDK5R1	0,0236	RGN	-0,0302	CARTPT	0,0204
SERPING1	-0,0231	AKAP7	0,0295	RELN	0,0203
NHLH2	0,0228	ADAMTS3	-0,0295	CCL2	-0,0202
CTGF	-0,0228	MYBL2	-0,0293	PTN	0,0201
EPB41L3	-0,0226	IGH@ /// IGHG1 /// IGHG2 /// IGHM /// IGHV4-31	0,0289	ID2 /// ID2B	-0,0200
CYP2E1	0,0225	NR4A2	-0,0286	UBE2C	0,0196
CYP17A1	-0,0222	TAC1	-0,0284	NOL4	-0,0195
CPB1	-0,0221	HLA-DRA	0,0284	SPOCK2	-0,0194
TEX14	0,0220	MAGEA6	-0,0282	HBA1 /// HBA2	0,0194
ADRB2	-0,0220	IGF2	-0,0280	DIRAS3	0,0190
DBH	-0,0219	CXCL14	-0,0278	PRKCB	0,0189
ELAVL4	-0,0216	SPARCL1	0,0277	CD53	-0,0188
CCL21	-0,0213	TYMS	0,0269	CYP21A2	0,0185
RABGAP1L	-0,0213	NDN	0,0263	STAR	-0,0183
GABBR1 /// UBD	-0,0212	UBE2C	0,0262	RGS7	0,0180
TST	0,0212	SFRP1	0,0262	ABCA8	-0,0178
DHCR24	-0,0212	ADAMTS1	-0,0259	CD163	-0,0175
PLXNC1	-0,0210	TEX14	0,0259	C7orf16	0,0172
RAMP3	-0,0208	GAGE1 /// GAGE12F /// GAGE12G /// GAGE12I /// GAGE12J /// GAGE2A /// GAGE2B /// GAGE2C /// GAGE2D /// GAGE2E /// GAGE3 /// GAGE4 /// GAGE5 /// GAGE6 /// GAGE7 /// GAGE8	-0,0258	POSTN	0,0172
DDC	-0,0204	CTGF	-0,0255	CALY	-0,0171
IGH@ /// IGHA1 /// IGHA2 /// IGHV3OR16-13 /// LOC100126583	0,0201	CKS2	0,0254	ZNF91	-0,0171
BAI3	-0,0201	ITGA8	0,0250	VSNL1	0,0169
AMPH	-0,0200	NEFH	-0,0247	DYNC1I1	-0,0169
TCL1A	0,0199	PMP22	0,0246	ZCCHC14	0,0169
SLC39A8	-0,0195	RRM2	0,0245	TEX14	0,0168
C3	-0,0193	FAIM3	0,0245	RABGAP1L	-0,0168
CALCB	-0,0185	MX1	-0,0244	BAI3	0,0166
DNAJB1	-0,0181	AGTR1	0,0240	ABCB1	0,0163
ODZ3	-0,0173	EPB41L4B	0,0239	TAC1	-0,0160
SCD5	-0,0173	DNAJB1	-0,0238	CYR61	-0,0158
ORM1	0,0171	FOXM1	-0,0232	MAGEA10	-0,0155
GSTA1	-0,0171	ABCB1	0,0227	COBL	0,0155
RBP4	0,0169	EFEMP1	-0,0227	CRYAB	-0,0153
IGH@ /// IGHG1 /// IGHG2 /// IGHM /// IGHV4-31	0,0169	CCL21	0,0226	CXCL2	0,0152
MEST	-0,0166	CTNNAL1	-0,0225	DTL	0,0151
EIF1AY	-0,0163	GSTA1	-0,0225	CGA	-0,0149
ASPN	0,0160	SMC4	0,0224	NEFH	-0,0148
FNBP1L	0,0159	CD52	0,0223	MGC39900 /// TMSL8	0,0146
FOS	-0,0159	BAMBI	-0,0220	GADD45B	-0,0144
S100A8	0,0159	NCAN	-0,0220	CCNB2	0,0144
IGHM	0,0158	MLF1IP	0,0207	GOLSYN	0,0141
MFF	0,0153	PRNP	0,0196	FOXM1	0,0138
HSPA6	-0,0152	RNASEH2A	0,0196	ADAMDEC1	-0,0137
TRBC1	-0,0147	IGH@ /// IGHA1 /// IGHA2 /// IGHV3OR16-13 /// LOC100126583	-0,0195	CKS2	0,0136
ZNF804A	0,0146	BAG3	-0,0193	GRP	-0,0135
A2BP1	-0,0146	CGA	-0,0193	MET	-0,0134
PLAC8	0,0143	KIF15	-0,0192	SCG2	0,0132
POU2AF1	0,0138	CD74	-0,0191	MATN2	-0,0131
IGF2	0,0136	IGK@ /// IGKC	0,0189	PMP22	0,0130
KNG1	0,0135	SLC47A1	0,0187	IGF2	0,0129
GNAI1	-0,0133	MMP9	0,0185	SMC4	0,0126
HLA-DRB4	0,0132	MCM2	0,0173	GAGE12F /// GAGE12G /// GAGE12I /// GAGE2A /// GAGE2B /// GAGE2E /// GAGE4 /// GAGE7	-0,0123
THBS4	-0,0130	FOS	0,0168	CDH18	0,0122
PTAFR	-0,0129	GRP	-0,0167	NR4A3	-0,0121
APOH	0,0128	AKR1C2	-0,0166	TYMS	0,0118
TAGLN	-0,0125	RET	-0,0166	MCM2	0,0117
FAM69A	-0,0123	GABRB1	0,0165	KIF2C	0,0117
NOL4	0,0122	GAGE12F /// GAGE12G /// GAGE12I /// GAGE2A /// GAGE2B /// GAGE2E /// GAGE4 /// GAGE7	-0,0162	LYZ	0,0117
DOCK4	-0,0116	CUX2	-0,0161	TNS3	-0,0116
PRKCA	0,0113	NEFL	-0,0158	CRH	-0,0114

ALB	0,0110	BUB1B	0,0158	ASCL1	0,0112
SPP1	-0,0110	TNS3	-0,0158	CXCL14	-0,0112
GP1BB	-0,0110	MATN2	0,0158	KIAA0746 /// SERINC2	-0,0112
AKAP11	0,0110	CYR61	-0,0157	ZWINT	0,0111
FGG	0,0109	CCNB1	-0,0154	MMP9	0,0111
NEFL	-0,0105	IGHM	0,0154	HRASLS3	-0,0111
TFAP2B	0,0104	FUT9	0,0154	PBK	0,0111
ADAMDEC1	0,0102	MAGEA9 /// MAGEA9B	0,0154	MLF1IP	0,0110
FAIM3	0,0101	PPL	0,0152	MFF	-0,0110
PRKCZ	-0,0098	GAGE1 /// GAGE12B /// GAGE12C /// GAGE12D /// GAGE12E /// GAGE12F /// GAGE12G /// GAGE12H /// GAGE12I /// GAGE12J /// GAGE13 /// GAGE2A /// GAGE2B /// GAGE2C /// GAGE2D /// GAGE2E /// GAGE4 /// GAGE5 /// GAGE6 /// GAGE7 /// GAGE8	-0,0151	MELK	0,0110
FGL1	0,0097	KIF20A	0,0151	TMEM194A	0,0109
SERPINF1	0,0096	DTL	0,0148	GMNN	0,0109
SERPINE2	-0,0095	DNASE1L3	0,0147	GAGE12B /// GAGE12C /// GAGE12D /// GAGE12E /// GAGE12F /// GAGE12G /// GAGE12H /// GAGE12I /// GAGE13 /// GAGE2A /// GAGE2B /// GAGE2C /// GAGE2E /// GAGE4 /// GAGE5 /// GAGE6 /// GAGE7	-0,0109
AMBP	0,0094	LOC100133662 /// RPS4Y1	-0,0147	ATP6V1G2	0,0105
GJA1	-0,0092	DHCR24	0,0145	NPY	0,0105
C4BPA	0,0092	DPEP3	0,0143	F12	-0,0101
FDX1	-0,0092	SPP1	-0,0141	ASPM	-0,0100
MAB21L2	-0,0091	GAGE1 /// GAGE12F /// GAGE12G /// GAGE12I /// GAGE12J /// GAGE2A /// GAGE2B /// GAGE2E /// GAGE4 /// GAGE5 /// GAGE6 /// GAGE7	-0,0139	DHCR24	0,0098
TDRD12	0,0090	S100A8	-0,0133	GAGE1 /// GAGE12B /// GAGE12C /// GAGE12D /// GAGE12E /// GAGE12F /// GAGE12G /// GAGE12H /// GAGE12I /// GAGE12J /// GAGE13 /// GAGE2A /// GAGE2B /// GAGE2C /// GAGE2D /// GAGE2E /// GAGE4 /// GAGE5 /// GAGE6 /// GAGE7 /// GAGE8	-0,0096
NAP1L3	0,0090	CRH	-0,0133	CCNB1	-0,0094
SCN3A	-0,0090	KIF2C	0,0133	CRISPLD2	-0,0092
APOC3	0,0089	POU2AF1	0,0127	ADRB2	0,0088
MAGEA9 /// MAGEA9B	-0,0088	TCL1A	0,0122	PTTG1	0,0087
CD53	0,0088	HLA-DPB1	0,0119	BIRC5	0,0086
MEIS2	0,0086	CCL2	-0,0117	KIAA1107	0,0085
RGN	-0,0084	AHNAK2	-0,0116	KCNK3	0,0085
HMGCS2	0,0084	GAL	0,0115	SEMA3B	-0,0084
APCS	0,0084	GINS2	0,0111	CXCL9	-0,0084
AHSG /// LOC100131613	0,0083	EIF1AY	-0,0111	MYBL2	-0,0081
ORM1 /// ORM2	0,0082	DDX1	0,0107	MIA	0,0078
ABCB1	-0,0082	CALY	0,0103	MEIS1	-0,0075
HPX	0,0081	TOP2A	0,0102	RAB3B	0,0074
SELL	0,0081	MET	-0,0100	MEST	0,0068
IL8	-0,0078	CPEB1	-0,0096	ZNHIT3	0,0067
LBP	0,0078	BIRC5	-0,0096	KIF15	-0,0064
CYP2C8	0,0077	RALYL	0,0095	FILIP1L	-0,0063
CD48	-0,0076	GAGE12B /// GAGE12C /// GAGE12D /// GAGE12E /// GAGE12F /// GAGE12G /// GAGE12H /// GAGE12I /// GAGE13 /// GAGE2A /// GAGE2B /// GAGE2C /// GAGE2E /// GAGE4 /// GAGE5 /// GAGE6 /// GAGE7	-0,0091	NAP1L3	0,0061
GC	0,0075	JARID1D	-0,0091	CTGF	-0,0060
SERPINC1	0,0074	CRISPLD2	-0,0089	TBC1D9	-0,0060
SAA4	0,0073	ASPM	0,0086	PPL	-0,0059
HBG1 /// HBG2	0,0072	HLA-DPA1	0,0085	TRIP13	-0,0058
CALB1	0,0070	MAP1A	0,0083	MAGEA11	-0,0057
FGA	0,0068	SERPINA5	0,0082	AKR1C2	0,0053
FUCA1	-0,0065	C7orf16	0,0077	MAB21L1	-0,0052
TBC1D9	-0,0065	CCNB2	-0,0076	CENPF	0,0049
CYP2B6 /// CYP2B7P1	0,0064	CYP21A2	-0,0073	FUCA1	0,0048
CRP	0,0063	SPOCK2	0,0071	RNF11	0,0047
PCDHA1 /// PCDHA10 /// PCDHA11 /// PCDHA12 /// PCDHA13 /// PCDHA2 /// PCDHA3 /// PCDHA4 /// PCDHA5 /// PCDHA6 /// PCDHA7 /// PCDHA8 /// PCDHA9 /// PCDHAC1 /// PCDHAC2	-0,0063	CCL18	0,0070	MAGEA9 /// MAGEA9B	-0,0047
CP	0,0058	HBA1 /// HBA2	0,0066	KIF20A	0,0046
VSNL1	-0,0057	DLGAP5	0,0066	AMIGO2	-0,0046
FGB	0,0057	PTN	0,0064	ADCY2 /// LOC100133953	-0,0044
HP /// HPR	-0,0057	MELK	0,0063	DNAJC6	0,0043
SFRP1	-0,0055	CPB1	0,0062	SEPP1	0,0042
IGK@ /// IGKC	0,0053	TRIP13	0,0057	PRIM1	0,0041
CARTPT	0,0052	CYP11B1	0,0052	C7	-0,0040
ID2 /// ID2B	-0,0047	CD163	0,0052	HSD3B2	-0,0040
TF	0,0046	KIF4A	0,0052	MCM6	-0,0039
APOA2	0,0046	CYP11A1	0,0051	BUB1B	-0,0038
ALDOB	0,0044	AGT	-0,0047	ASS1	-0,0036
C2CD2	0,0043	MAGEA11	0,0047	SV2B	-0,0035
IFI44L	0,0042	IFI44L	0,0046	PRKCZ	0,0035
AGTR1	-0,0041	PTTG1	-0,0045	FDX1	-0,0034
ZNF91	0,0039	PBK	0,0042	FNBP1L	0,0033
CD163	0,0037	CDC20	0,0042	DNASE1L3	0,0032
CXCL14	-0,0036	IGL@ /// IGLC2 /// IGLV2-14	0,0038	MAGEA6	-0,0031
OPHN1	0,0032	FOSB	-0,0038	NEBL	-0,0030
LYZ	0,0030	C7	0,0037	GAGE1 /// GAGE12F /// GAGE12G /// GAGE12I /// GAGE12J /// GAGE2A /// GAGE2B /// GAGE2E /// GAGE4 /// GAGE5 /// GAGE6 /// GAGE7	0,0030
PIK3R3	-0,0028	OLFML2A	0,0035	XAGE1A /// XAGE1B /// XAGE1C /// XAGE1D /// XAGE1E	-0,0027
DPEP3	-0,0027	TMEM194A	0,0035	HLA-DRB4	0,0026
SV2C	-0,0026	HBB	-0,0029	GAL	0,0025
DDAH1	-0,0025	MYCN	-0,0029	PCNA	0,0023
HLA-DQA1 /// HLA-DQA2	-0,0025	FILIP1L	-0,0027	FAM64A	-0,0020
IGL@ /// IGLC2 /// IGLV2-14	0,0025	C2orf43	-0,0027	PIK3R3	0,0018
NDN	0,0024	CYP17A1	0,0023	GAGE1 /// GAGE12F /// GAGE12G /// GAGE12I /// GAGE12J /// GAGE2A /// GAGE2B /// GAGE2C /// GAGE2D /// GAGE2E /// GAGE3 /// GAGE4 /// GAGE5 /// GAGE6 /// GAGE7 /// GAGE8	-0,0016
AGT	0,0024	HLA-DMA	0,0021	NMU	-0,0015
GPR22	0,0023	PLAT	-0,0018	DCN	0,0015
EFEMP1	0,0023	FAM64A	-0,0017	FGF13	-0,0015
CD52	-0,0022	ALDH1A2	0,0017	KIAA0101	0,0010
KIAA0746 /// SERINC2	0,0021	GABBR1 /// UBD	0,0012	OPHN1	0,0009
ARG1	0,0021	CARTPT	0,0009	GINS2	0,0008
EPB41L4B	0,0020	MAGEA10	-0,0009	PCDHA1 /// PCDHA10 /// PCDHA11 /// PCDHA12 /// PCDHA13 /// PCDHA2 /// PCDHA3 /// PCDHA4 /// PCDHA5 /// PCDHA6 /// PCDHA7 /// PCDHA8 /// PCDHA9 /// PCDHAC1 /// PCDHAC2	-0,0007
TRBC1 /// TRBC2 /// TRBV19	-0,0018	ATF3	-0,0008	DDAH1	0,0006
ST6GALNAC5	-0,0015	DDX3Y	-0,0007	CYP17A1	-0,0005
GSPT2	0,0015	CENPF	-0,0005	CYP11B1	-0,0004
MEIS1	-0,0012	NPY	0,0005	DPEP3	-0,0003

VIP	-0,0005	ARID5B	-0,0003	CPB1	0,0002
KCNK3	0,0004	FDX1	0,0002	RCAN2	0,0002
ITGB2	-0,0004	MAGEA4	-0,0002	CDC20	-0,0001
APOA1	0,0003	BHLHB2	-0,0001	CYP11A1	0,0001
ALDH1A2	-0,0003	HSD3B2	-0,0001	ITGA8	0,0000

McArdle/Wilzén data set

Variable	PC1	Variable	PC2	Variable	PC3
PLP1	-0,0993	FOXM1	0,1061	LOC100133662 /// RPS4Y1	0,1002
S100B	-0,0919	FABP4	0,1039	PENK	0,0988
APOD	-0,0917	RRM2	0,1010	CUX2	0,0862
CDH19	-0,0893	KIF11	0,1006	ASS1	0,0805
ABCA8	-0,0886	ASPM	0,0940	GULP1	0,0746
FXYD1	-0,0836	IGH@ /// IGHG1 /// IGHG2 /// IGHM /// IGHV4-31	0,0939	DLK1	0,0743
ARHGAP15	-0,0835	BUB1B	0,0932	ATP10D	0,0739
ERBB3	-0,0792	KIF4A	0,0911	GLDC	0,0724
SEMA3B	-0,0790	PBK	0,0895	DDX3Y	0,0670
ST6GALNAC2	-0,0786	ST6GALNAC5	-0,0866	DUSP4	0,0657
PMP2	-0,0784	DTL	0,0861	SEMA5A	0,0635
ITIH5	-0,0782	CDKN3	0,0850	EFS	0,0611
MAL	-0,0761	KIF15	0,0848	IL8	0,0610
SERPINA3	-0,0756	CDC20	0,0845	GAL	0,0606
ALDH1A1	-0,0755	ALDH1A2	0,0838	EIF1AY	0,0582
ASPA	-0,0746	ZWINT	0,0833	JARID1D	0,0577
ISL1	0,0743	TTK	0,0830	ETV1	0,0563
MYOT	-0,0741	FAM64A	0,0820	KIF15	0,0551
CRYAB	-0,0736	KIF20A	0,0809	RAI14	0,0523
RELN	-0,0731	EFEMP1	0,0795	MFAP4	0,0521
PTPRZ1	-0,0712	CCNB2	0,0788	KIF20A	0,0517
GPM6B	-0,0710	CREB5	-0,0786	CHODL	0,0517
COL14A1	-0,0709	ESPL1	0,0785	CRABP1	0,0511
F3	-0,0697	CDC2	0,0783	CKS2	0,0502
NR4A2	-0,0685	TPX2	0,0782	FAM64A	0,0502
VIP	-0,0683	RAMP3	-0,0779	PTX3	0,0501
NRN1	-0,0680	NEK2	0,0776	HSPA6	0,0493
SOX10	-0,0673	PTTG1	0,0764	TWIST1	0,0492
GPR126	-0,0667	KIF14	0,0763	SLC19A2	0,0490
HEPH	-0,0662	SV2C	-0,0763	SNX7	0,0486
CHL1	-0,0652	TOP2A	0,0760	CXCL2	0,0474
MATN2	-0,0650	SERPINF1	0,0748	CCNB1	0,0473
SRPX	-0,0650	SMC4	0,0739	NHLH2	0,0472
ZNF804A	0,0645	SH3GL3	0,0739	TGIF1	0,0472
CX3CR1	-0,0642	CENPF	0,0738	PDE10A	0,0465
SOX11	0,0640	TYMS	0,0730	CCNB2	0,0465
LAMA2	-0,0639	INSM1	-0,0727	PTGS2	0,0462
F13A1	-0,0638	PRC1	0,0721	ECT2	0,0455
ATP1A2	-0,0637	EZH2	0,0716	TPBG	0,0454
PLSCR4	-0,0636	CHL1	-0,0716	ASCL1	0,0448
MAB21L2	0,0635	CCNB1	0,0712	KAL1	0,0438
ZCCHC24	-0,0635	BIRC5	0,0711	PRSS3	0,0434
PLEKHB1	-0,0633	IGK@ /// IGKC	0,0707	ASPM	0,0433
IL7	0,0632	DIXDC1	-0,0701	CENPF	0,0425
STARD13	-0,0627	GINS2	0,0696	ENTPD4 /// LOXL2	0,0424
PRC1	0,0624	MMP12	0,0694	NETO2	0,0423
C3	-0,0623	GAS1	0,0691	SLIT1	0,0423
C10orf116	-0,0623	C3	0,0690	FBP1	0,0406
LGI1	-0,0622	IGH@ /// IGHA1 /// IGHA2 /// IGHV3OR16-13 /// LOC100126583	0,0689	TMEM158	0,0402
MOXD1	-0,0620	CD163	0,0687	IGF2	0,0398
ADCYAP1	-0,0617	KIF2C	0,0685	HMG20B	0,0396
GATA3	0,0615	CCNA2	0,0682	MYO10	0,0393
MGC39900 /// TMSL8	0,0613	PDGFRA	0,0669	PBK	0,0391
FBLN5	-0,0612	MPHOSPH8	-0,0667	TTK	0,0390
MPZ	-0,0608	ECT2	0,0663	CREB5	0,0390
RRM2	0,0608	ZNF365	-0,0662	DLGAP5	0,0389
IGFBP6	-0,0602	BLM	0,0662	FGF1	0,0389
P2RY14	-0,0600	NUSAP1	0,0662	CDKN3	0,0388
KCNQ2	0,0600	CDH18	-0,0658	PTTG1	0,0384
CACNA2D3	0,0596	KIF18B	0,0654	FOXM1	0,0382
OGN	-0,0595	ZBTB38	-0,0649	ADM	0,0381
CYP1B1	-0,0595	NCAN	0,0649	PMAIP1	0,0380
SDC4	-0,0593	HJURP	0,0648	BUB1B	0,0379
DCN	-0,0589	MELK	0,0645	FOSL2	0,0375
ANGPTL7	-0,0579	CKS2	0,0644	ZNF536	0,0375
UTS2	-0,0577	FANCI	0,0643	ADAMTS5	0,0365
RNASE4	-0,0576	SELL	0,0641	COLEC12	0,0362
EGFL8 /// PPT2	-0,0575	LOC100130100	0,0640	TTTY15	0,0361
DTL	0,0574	UBE2C	0,0640	RRM2	0,0357
WFDC1	-0,0572	MAD2L1	0,0639	CENPE	0,0356
ABCG2	-0,0569	CD36	0,0636	LOC100130216 /// USP9Y	0,0354
CFH	-0,0567	PLD3	-0,0636	NMU	0,0350
NBLA00301	0,0567	CDC45L	0,0632	CDC2	0,0349
SLCO1C1	-0,0565	DLGAP5	0,0627	CCNA2	0,0344
NOL4	0,0562	ERC1	-0,0627	KIF14	0,0343
CFI	-0,0561	TRIP13	0,0625	MAD2L1	0,0340
RHBDF1	-0,0560	CENPE	0,0625	AURKB	0,0338
OLFML3	-0,0552	MLF1IP	0,0616	MLF1IP	0,0332
GDAP1L1	0,0552	FEN1	0,0616	POPDC3	0,0328
TOP2A	0,0547	DNA2	0,0616	FZD2	0,0326
C4A /// C4B	-0,0546	PMAIP1	-0,0615	GALNT6	0,0323
DBH	0,0545	ABCB1	-0,0615	BMP7	0,0318
COBL	-0,0541	IGHM	0,0614	CDCA4	0,0313
PDZD2	-0,0539	FAM115A /// FAM115B	-0,0614	TRIP13	0,0310
ACTL6B	0,0538	ATP1A3	-0,0611	SULF1	0,0308
CAPN6	-0,0536	DGKB	-0,0609	BAG3	0,0308
COL9A3	-0,0535	RFC4	0,0608	GPR177	0,0308
GPR137B	-0,0534	MS4A6A	0,0607	TPX2	0,0305
FUT9	0,0534	KIF3B	-0,0607	PDLIM4	0,0304
SFRP4	-0,0534	ENDOG	0,0607	EZH2	0,0303
DAAM2	-0,0531	ODZ3	-0,0605	RXRG	0,0297
WWTR1	-0,0529	MCM2	0,0604	CDC45L	0,0295
ADCY1	0,0528	TUBB2A /// TUBB2B	-0,0600	BLM	0,0294
NT5DC2	0,0528	FBLN1	0,0600	SERPINE1	0,0292
MAD2L1	0,0525	PLAC8	0,0599	KIF11	0,0292
GINS2	0,0523	IGL@ /// IGLC2 /// IGLV2-14	0,0593	SMC4	0,0290
HRASLS3	-0,0523	MKI67	0,0584	HBG1 /// HBG2	0,0287
BUB1B	0,0517	HOXC10	-0,0583	CYP1B1	0,0281
EMP2	-0,0517	CDCA4	0,0581	NUSAP1	0,0278
GAS7	-0,0516	METTL7A	0,0577	INHBA	0,0276
AHR	-0,0516	SULF1	0,0577	TYMS	0,0273
MAB21L1	0,0516	DCN	0,0573	UBE2C	0,0271
PLLP	-0,0514	EIF3I	-0,0570	FANCI	0,0269
MEOX2	-0,0514	ZKSCAN1	-0,0568	CGA	0,0268

ENDOD1	-0,0513	PIK3R1	-0,0565	HBA1 /// HBA2	0,0266
PDGFRL	-0,0512	ZNF91	-0,0563	ERBB4	0,0263
MIA	-0,0512	SRPX	0,0563	NEK2	0,0262
DHRS3	-0,0510	POSTN	0,0563	BIRC5	0,0260
TGFBR3	-0,0510	CHD5	-0,0556	KIF4A	0,0259
EZH2	0,0509	ADAMDEC1	0,0555	LIF	0,0254
NKAIN1	0,0509	CHRDL1	0,0551	CALB1	0,0254
ENOX1	0,0508	RPH3A	-0,0550	TLE2	0,0251
CRLF1	-0,0508	CDH4	0,0547	ADAMTS1	0,0248
SLC38A1	0,0506	NEBL	-0,0544	HBB	0,0247
FCGBP /// LOC100133944	-0,0505	DENND2D	0,0543	PLAT	0,0247
CD302	-0,0505	HIST1H2AC	0,0542	SOX9	0,0247
FOXM1	0,0505	PDAP1	-0,0541	VIP	0,0244
PON2	-0,0504	SHANK2	-0,0540	IGF2BP3	0,0242
SEMA3C	-0,0504	STAT3	-0,0540	GATA6	0,0242
GATM	-0,0502	ADCYAP1	-0,0537	HJURP	0,0240
LPL	-0,0502	PLA2G2A	0,0537	POSTN	0,0239
CXADR	0,0502	CILP	0,0535	G0S2	0,0237
GPC3	-0,0501	FAIM3	0,0532	S100A8	0,0237
PTX3	-0,0501	FLJ22662	0,0530	FAM114A1	0,0236
CHST1	0,0499	NTRK1	-0,0526	CXCR7	0,0235
EDNRB	-0,0499	CSGALNACT1	0,0520	SV2C	0,0234
MCM2	0,0498	GINS1	0,0519	ST6GALNAC5	0,0233
C1R	-0,0497	EPHA5	-0,0515	F12	0,0232
SOX9	-0,0497	GPR22	-0,0514	PDGFA	0,0229
ANXA1	-0,0496	MS4A4A	0,0508	TOP2A	0,0227
OLFML2A	-0,0496	C1S	0,0507	IL6	0,0227
EGR3	-0,0495	ATAD2	0,0507	GOLM1	0,0226
PLAT	-0,0495	AKAP7	-0,0503	LOC348162 /// LOC642799 /// LOC729602	0,0225
CDH1	-0,0494	CXorf57	0,0502	RFC4	0,0225
GRIA2	0,0494	YIPF5	-0,0502	RPH3A	0,0225
ADAMTS8	-0,0492	ACHE	-0,0502	MCM2	0,0217
ADAM22	0,0491	OGDH	-0,0502	CTNNAL1	0,0217
CCNB2	0,0491	CCL19	0,0501	GABRP	0,0217
LOC284244	0,0491	CTSZ	0,0501	MELK	0,0212
VGLL3	-0,0490	EEF1A2	-0,0500	EPB41L4B	0,0211
PBK	0,0488	S100A8	0,0500	PRC1	0,0208
PLEKHA4	-0,0488	AURKB	0,0495	KIF18B	0,0205
MICALL2	-0,0486	LEF1	0,0493	DTL	0,0201
AHNAK	-0,0481	LYZ	0,0493	MCM10	0,0201
KIF11	0,0480	HLA-DQA1 /// HLA-DQA2	0,0493	HMGB3	0,0198
GRAMD3	-0,0480	XIST	0,0490	GINS2	0,0195
COL21A1	-0,0478	ABCB1 /// ABCB4	-0,0489	NBLA00301	0,0194
GNG12	-0,0475	LOC100133662 /// RPS4Y1	-0,0484	NELL1	0,0194
TNXB	-0,0475	EPB41L3	-0,0480	TNFAIP6	0,0190
ADD2	0,0472	NNMT	0,0479	HELLS	0,0190
PAFAH1B3	0,0471	CTGF	0,0478	FABP6	0,0189
FOSL2	-0,0471	KIF5C	-0,0478	KLF9	0,0186
KANK2	-0,0468	PRSS3	-0,0476	BCL2A1	0,0186
C8orf4	-0,0467	CALY	-0,0475	GPM6B	0,0185
APBA2	0,0467	MCM10	0,0473	DNA2	0,0184
CXCL14	-0,0467	CD48	0,0473	BACE2	0,0183
FAM107A	-0,0467	LOXL1	0,0472	ADAMTS8	0,0180
C1S	-0,0466	ALDOC	-0,0472	CNN3	0,0178
THBS4	-0,0466	CAMK2B	-0,0472	PAWR	0,0177
CELSR3 /// SLC26A6	0,0464	CFD	0,0469	ENDOG	0,0176
FMO3	-0,0464	EYA4	-0,0467	PON2	0,0173
GULP1	-0,0464	P2RX5	0,0467	GINS1	0,0172
EPB41L2	-0,0464	HBB	0,0466	ARHGEF10	0,0172
PRELP	-0,0463	CGA	-0,0461	KIF2C	0,0170
LOC157627	0,0462	POU4F2	-0,0457	LRBA	0,0165
EYA1	0,0462	PTGFR	-0,0457	MKI67	0,0163
MT1M	-0,0461	SERPINB9	0,0456	PLCE1	0,0159
SLC18A1	0,0461	CDH12	-0,0456	ANKRD57	0,0159
FMO1	-0,0461	CRIP2	-0,0453	ZWINT	0,0159
KLF9	-0,0460	IL10RA	0,0448	CD24	0,0158
TNFAIP6	-0,0459	PDE10A	-0,0448	SLC6A15	0,0156
ALDH1A3	-0,0459	RAI14	0,0447	CDC20	0,0156
CTNNAL1	-0,0459	SFRP4	0,0445	PDE8B	0,0156
GINS1	0,0459	CALB1	-0,0445	ESPL1	0,0154
IGF2BP3	0,0459	MEIS2	-0,0442	ATAD2	0,0154
MT1F	-0,0458	LRBA	0,0442	TBC1D30	0,0149
LUM	-0,0457	GUCA1A	-0,0441	PCDH8	0,0147
ERBB2	-0,0456	GOLM1	-0,0437	EIF2S3	0,0145
MET	-0,0455	LOC100127887 /// SYT2	-0,0432	hCG_1644608 /// SET	0,0142
ADH1B	-0,0455	MRC1 /// MRC1L1	0,0431	RELN	0,0137
RGS7	0,0454	HMOX1	0,0431	MPHOSPH8	0,0137
ITGB4	-0,0453	LUM	0,0429	SLC2A14 /// SLC2A3	0,0136
TAC1	-0,0450	MXRA5	0,0429	KCNS3	0,0134
ITGA6	-0,0449	CCR7	0,0428	MT1M	0,0133
TNS3	-0,0449	C1R	0,0427	FNDC3B	0,0133
FAM64A	0,0449	KIF1A	-0,0427	THBS4	0,0132
SASH1	-0,0449	ENTPD4 /// LOXL2	0,0427	PCSK2	0,0131
CHRDL1	-0,0449	VSIG4	0,0425	GPR137B	0,0131
STMN4	0,0447	ISLR	0,0425	PDGFRA	0,0131
EEF1A2	0,0447	HMGB3	0,0425	INSM1	0,0130
TRAM2	-0,0446	RAB3B	-0,0424	LDLR	0,0124
GPR177	-0,0444	SCARB2	-0,0422	PRND	0,0118
MT1E	-0,0443	CXCL12	0,0421	THBD	0,0114
NETO2	0,0443	AMIGO2	-0,0421	MEX3D	0,0114
C1orf165	0,0443	CCL21	0,0421	MAOB	0,0113
TNXA /// TNXB	-0,0442	PTPRC	0,0419	STC1	0,0109
SCN3B	0,0441	EFS	0,0418	HEPH	0,0109
CGA	-0,0441	ZNF804A	0,0418	P2RX5	0,0108
BHLHB3	-0,0440	FOS	0,0418	ADCYAP1	0,0108
DKFZP586H2123	-0,0440	CCL3 /// CCL3L1 /// CCL3L3 /// LOC728830	0,0416	DIXDC1	0,0108
DPYSL4	0,0439	HLA-DMA	0,0416	TRAM2	0,0107
SLC4A8	0,0439	GALNT6	-0,0414	CRLF1	0,0107
THBD	-0,0438	TRBC1	0,0412	POU4F2	0,0106
THBS1	-0,0436	HCLS1	0,0405	NID2	0,0106
FZD2	-0,0436	MAPT	-0,0403	LRRC37A /// LRRC37A2	0,0098
LOC100134306 /// MYT1L	0,0435	MFAP5	0,0403	THBS1	0,0098
NELL2	0,0435	C7	0,0402	LOC653188 /// SMA4 /// SMA5	0,0098
TPX2	0,0433	IGL@	0,0399	UTS2	0,0098
KAL1	-0,0432	RASSF2	0,0396	CCL11	0,0097
MT1X	-0,0432	ADH1B	0,0396	EIF3I	0,0097
HAND1	0,0431	CFH	0,0394	MET	0,0096
MAGI1	0,0430	EVI2B	0,0394	PTPRK	0,0093
LDLR	-0,0430	LOC100133233 /// TRAF3IP3	0,0391	KCNE4	0,0092
TSPAN8	-0,0429	CTSH	0,0391	KDELR3	0,0091
CRISPLD2	-0,0429	TFAP2B	-0,0390	SCARB2	0,0090
RIMBP2	0,0428	CCL18	0,0390	F3	0,0087
MRC1 /// MRC1L1	-0,0428	HLA-DRB4	0,0389	CALB2	0,0085

TTK	0,0426	ACVR2B	-0,0388	HAS2	0,0084
METTL7A	-0,0426	PDE8B	-0,0387	MAGEA4	0,0083
DPP6	0,0425	JUP /// KRT19	0,0387	MXRA5	0,0083
CXCL12	-0,0425	PDGFC	0,0387	ATF3	0,0082
DUSP5	-0,0424	FUT9	0,0386	EGFL8 /// PPT2	0,0081
KDELR3	-0,0423	BMP7	0,0386	SERPINA3	0,0081
HLA-DMA	-0,0422	FAM153A /// FAM153B /// FAM153C	0,0384	ZWILCH	0,0081
GLT8D2	-0,0422	ZWILCH	0,0383	SIX3	0,0080
FABP6	0,0420	GNAO1	-0,0382	RSRC1	0,0079
TYMS	0,0419	ADIPOQ	0,0381	LPL	0,0079
ANKRD57	-0,0419	PTGER4	0,0379	DKFZP586H2123	0,0077
CCL2	-0,0419	RAC3	-0,0378	MGC39900 /// TMSL8	0,0077
SERPINF1	-0,0417	TRBC1 /// TRBC2 /// TRBV19	0,0377	AQP1	0,0076
DISC1	-0,0416	CXCR7	0,0375	NELL2	0,0076
CENPF	0,0415	ERBB4	-0,0375	OLFML2A	0,0074
ASPM	0,0414	INHBA	0,0374	CCDC68	0,0073
PTGDS	-0,0413	GZMB	-0,0373	STARD13	0,0072
ZEB2	-0,0412	MGC39900 /// TMSL8	0,0373	EMP2	0,0070
ZWINT	0,0412	FEV	0,0371	EGR3	0,0069
SORBS1	-0,0412	DDX3Y	-0,0370	CCL3 /// CCL3L1 /// CCL3L3 /// LOC728830	0,0066
INSM1	0,0411	TPBG	0,0370	FXYD1	0,0063
KCNE4	-0,0411	SEMA3E	-0,0369	SDC4	0,0062
NAP1L2	-0,0411	CYR61	0,0368	GDAP1L1	0,0058
SLC6A2	0,0410	JARID1D	-0,0368	COL21A1	0,0057
RARRES2	-0,0410	CTNND2	-0,0363	FMO3	0,0056
NEK2	0,0410	IGKC	0,0363	EMP1	0,0055
ISLR	-0,0409	SLC18A1	-0,0363	NDUFS7	0,0055
MYT1	0,0409	MMP9	0,0362	ASPN	0,0055
ARHGEF10	-0,0409	RIT2	-0,0361	HMOX1	0,0051
FGF13	0,0406	hCG_1644608 /// SET	-0,0360	SERPINF1	0,0049
SCN7A	-0,0405	LY96	0,0359	OLFML2B	0,0048
CNTNAP2	0,0405	PEG3 /// ZIM2	-0,0359	SLCO1C1	0,0047
IL6	-0,0405	ITM2A	0,0359	VGLL3	0,0046
NGFR	-0,0404	CYP1B1	0,0358	OLFML3	0,0046
FCGR2B	-0,0404	SNRPN	-0,0355	NR4A2	0,0045
SPON2	-0,0404	CXCL2	0,0352	DUSP5	0,0044
PCSK1	-0,0402	MAGEA12	-0,0351	GAS1	0,0043
ZFHX3	0,0401	LCK	0,0351	FAM107A	0,0043
CD24	0,0401	LTBP2	0,0350	COCH	0,0042
HAS2	-0,0401	RALYL	-0,0349	CHL1	0,0042
RIMS3	0,0401	COL14A1	0,0347	DAAM2	0,0041
EFNA5	0,0401	EIF2S3	-0,0346	NOL4	0,0041
DIRAS3	-0,0400	GABBR1 /// UBD	0,0341	CXCL14	0,0041
FAM114A1	-0,0398	FLRT2	0,0335	OGN	0,0038
RFC4	0,0398	CD3D	0,0334	MAFF	0,0037
PDGFC	-0,0397	OLFML2B	0,0333	NT5DC2	0,0033
TRIP13	0,0397	FXYD5	0,0333	GPX1	0,0033
CFH /// CFHR1	-0,0396	TCL1A	0,0331	ITGA6	0,0032
MMP9	0,0396	ZNF652	-0,0328	PTPRZ1	0,0032
VSIG4	-0,0389	EPB41L4B	-0,0328	GNG12	0,0030
S100A6	-0,0388	FAM5C	0,0328	TNXB	0,0030
IGF1	-0,0388	MS4A1	0,0327	F13A1	0,0028
ADAMTS1	-0,0387	KCNK3	-0,0327	SEMA3C	0,0028
FOSB	-0,0387	ASCL1	0,0326	FEN1	0,0026
FNDC3B	-0,0380	SPOCK2	-0,0326	TNXA /// TNXB	0,0025
HMGB3	0,0380	MAGEA4	-0,0326	ACVR2B	0,0024
CCNB1	0,0378	PLSCR4	0,0325	CELSR3 /// SLC26A6	0,0023
KIF14	0,0377	PTPRK	0,0325	SEMA3B	0,0023
TUBB2A /// TUBB2B	0,0377	FMO1	0,0325	COL9A3	0,0021
DAB2	-0,0376	REEP1	-0,0325	SOX11	0,0020
PALM2-AKAP2	-0,0375	LTB	0,0324	PAFAH1B3	0,0020
ACVR2B	0,0375	POPDC3	-0,0324	ATP1A2	0,0019
KIF20A	0,0375	IL1R1	0,0323	PMP2	0,0018
TFAP2B	0,0374	THBS2	0,0322	FLJ22662	0,0015
OLFML2B	-0,0374	F13A1	0,0322	GZMB	0,0013
NUSAP1	0,0374	SLITRK5	0,0320	PLLP	0,0011
NCAN	0,0373	IGF2BP3	0,0316	VSIG4	0,0008
TUBB4	0,0373	CSAG2 /// CSAG3	-0,0313	ITGB4	0,0008
KIF4A	0,0372	CAV1	0,0312	CILP	0,0007
BACE2	-0,0372	IGF2	0,0312	GPR126	0,0007
HSPA12A	-0,0371	CFH /// CFHR1	0,0311	SASH1	0,0005
EMCN	-0,0371	GPX1	-0,0310	DHRS3	0,0003
IFIT1	-0,0371	PCDH7	0,0309	ANGPTL7	0,0001
FGL2	-0,0371	ATP6V1G2	-0,0308	SLC38A1	0,0001
TGIF1	-0,0370	CD52	0,0308	ST6GALNAC2	0,0001
EMP1	-0,0370	LOC348162 /// LOC642799 /// LOC729602	-0,0307	PDAP1	-0,0001
MAFF	-0,0370	RSRC1	-0,0307	CAV1	-0,0002
UBE2C	0,0369	GJA1	0,0307	MICALL2	-0,0002
TOX3	0,0369	COLEC12	0,0307	WFDC1	-0,0002
IER3	-0,0368	TRIM22	0,0303	PLEKHA4	-0,0003
TRIM22	-0,0367	DIRAS3	-0,0300	ENDOD1	-0,0003
KIF15	0,0366	FASN	-0,0299	CTGF	-0,0003
NID2	-0,0366	LY6H	0,0298	THBS2	-0,0007
RXRG	-0,0366	LOC652493	0,0297	RHBDF1	-0,0008
RAC3	0,0366	PPP1R1A	0,0296	LAMA2	-0,0008
NNMT	-0,0365	EYA1	-0,0295	FCGBP /// LOC100133944	-0,0011
CEBPB	-0,0365	VSNL1	-0,0294	EDNRB	-0,0011
MLF1IP	0,0364	CNN3	-0,0294	GJA1	-0,0012
FLJ22662	-0,0363	DAB2	0,0293	MT1X	-0,0012
MAPT	0,0363	LRRC37A /// LRRC37A2	-0,0293	SOX10	-0,0013
HMG20B	-0,0362	OLFML3	0,0289	TM4SF1	-0,0013
SH3GL3	0,0361	C4A /// C4B	0,0288	ERBB3	-0,0013
BIRC5	0,0359	PCDH8	0,0287	CAPN6	-0,0016
CDC20	0,0358	KANK2	0,0287	MPZ	-0,0016
JUP /// KRT19	0,0358	DARC	0,0287	SST	-0,0018
TM4SF1	-0,0357	PRELP	0,0286	ABCG2	-0,0018
MYO10	-0,0356	LOC653188 /// SMA4 /// SMA5	-0,0284	CFI	-0,0018
EPB41L4B	-0,0355	AMFR	-0,0284	GPC3	-0,0019
SCN3A	0,0354	NPY	0,0283	C8orf4	-0,0019
RALGPS1	0,0354	KDELR3	0,0283	CEBPB	-0,0019
LIF	-0,0353	ADAMTS5	0,0282	LOC157627	-0,0021
CRH	-0,0352	MET	-0,0281	TSPAN8	-0,0021
ZNF536	-0,0352	CCL2	0,0279	SLC4A8	-0,0023
GATA2	0,0351	ELAVL2	-0,0279	ABCB1	-0,0023
ETV1	-0,0349	FAM69A	-0,0279	MOXD1	-0,0024
VSNL1	0,0347	CD24	-0,0278	ALDH1A3	-0,0024
AHNAK2	-0,0347	GABRB1	-0,0277	SPON2	-0,0024
CKS2	0,0344	MEOX2	0,0273	AMFR	-0,0025
CHRNA3	0,0341	MAB21L2	0,0273	CXADR	-0,0025

FGF1	-0,0340	TTTY15	-0,0273	MT1E	-0,0026
COLEC12	-0,0339	PDGFD	0,0272	CHRDL1	-0,0028
ALOX5AP	-0,0336	IL7	0,0272	KANK2	-0,0029
FEN1	0,0335	COPG2IT1	-0,0271	RNASE4	-0,0030
ATF3	-0,0335	SYT17	-0,0270	GAS7	-0,0030
CAMK2B	0,0335	NRN1	-0,0269	NCAN	-0,0031
RASSF2	-0,0334	CXCL10	0,0269	EPB41L2	-0,0031
PDGFRA	-0,0333	G0S2	0,0269	SLITRK5	-0,0034
F12	0,0333	TH	-0,0268	FCGR2B	-0,0035
CCNA2	0,0333	A2BP1	-0,0268	HSD3B2	-0,0035
AQP1	-0,0333	TAC1	0,0268	C10orf116	-0,0038
LY96	-0,0332	SCN3A	-0,0268	CDH1	-0,0038
MMP12	0,0332	EIF1AY	-0,0267	SRPX	-0,0039
TH	0,0331	AK5	-0,0266	PDZD2	-0,0039
MS4A4A	-0,0329	FBLN5	0,0266	ASPA	-0,0040
C7	-0,0328	RELN	-0,0265	EYA4	-0,0040
ATP1A3	0,0328	KLF4	0,0265	ISL1	-0,0040
MS4A6A	-0,0326	MAGEA6	-0,0264	MIA	-0,0042
INHBA	-0,0326	IGK@ /// IGKC /// IGKV3-20 /// IGKV3D-11 /// IGKV3D-15	0,0264	PDGFRL	-0,0043
KIF5C	0,0324	MFAP4	0,0264	PCP4	-0,0044
SNF1LK	-0,0324	ABCA8	0,0263	ALOX5AP	-0,0044
PDGFA	-0,0323	PRAME	0,0261	GLT8D2	-0,0045
PLCE1	-0,0323	HTR3A	-0,0260	HTR3A	-0,0045
TBC1D30	0,0322	F12	0,0260	PCSK1	-0,0049
ESPL1	0,0322	PDLIM4	-0,0258	FOS	-0,0055
ZWILCH	0,0321	OSBPL3	0,0258	FOSB	-0,0055
MELK	0,0319	IGF1	0,0257	WWTR1	-0,0056
ADAMTS5	-0,0319	IFI44L	0,0257	KIF3B	-0,0057
KLF4	-0,0318	MAGEA3	-0,0255	MYOT	-0,0059
THBS2	-0,0317	IL6	0,0254	CDH18	-0,0060
POU4F2	0,0317	POU2AF1	0,0253	EFNA5	-0,0061
GNG3	0,0317	FGL2	0,0253	ITIH5	-0,0061
SLC6A15	0,0317	PRNP	-0,0253	FBLN1	-0,0062
NTRK1	0,0314	PLAT	-0,0253	MEIS2	-0,0065
BAG3	-0,0314	BACE2	0,0252	SCN7A	-0,0066
DDC	0,0314	ZFPM2	0,0252	ERBB2	-0,0066
DARC	-0,0313	SLC19A2	0,0251	IGF1	-0,0068
ITM2A	-0,0312	GPR137B	0,0249	MEOX2	-0,0068
IL1R1	-0,0311	TGIF1	0,0246	RBP4	-0,0068
KIF18B	0,0310	ALOX5AP	0,0245	MFAP5	-0,0070
KCNK3	0,0309	SEMA5A	0,0245	ZNF652	-0,0071
LRRN3	0,0309	ZCCHC24	0,0244	CCL8	-0,0072
CXCL2	-0,0309	SPON2	0,0241	GRAMD3	-0,0073
SIX3	0,0308	FXYD7	0,0241	BHLHB3	-0,0075
PDGFD	-0,0308	KLF9	-0,0241	ADAM22	-0,0076
CDH4	0,0307	ADCY2 /// LOC100133953	-0,0241	TGFBR3	-0,0077
COCH	0,0307	LOC100130216 /// USP9Y	-0,0240	LGI1	-0,0079
PRNP	-0,0307	GRIA2	-0,0240	AHNAK	-0,0079
GNAO1	0,0307	DUSP4	-0,0238	S100B	-0,0079
CDC2	0,0306	ARHGEF10	0,0238	IGFBP6	-0,0081
IFI44L	-0,0305	PTGDS	0,0237	NTRK3	-0,0081
MEG3	0,0305	HLA-DRA	0,0235	CX3CR1	-0,0082
PLA2G2A	-0,0305	CD302	0,0234	RIMBP2	-0,0082
KIF2C	0,0305	SNX7	-0,0233	TOX3	-0,0083
FAM115A /// FAM115B	0,0305	CCL8	0,0233	FLRT2	-0,0084
AURKB	0,0304	KIAA0644	-0,0232	P2RY14	-0,0084
TPBG	-0,0304	BCL2A1	0,0231	IFIT3	-0,0085
HLA-DRA	-0,0304	ACTA1	0,0231	MEG3	-0,0086
PRAME	0,0303	ABLIM3	-0,0229	MATN2	-0,0088
PENK	-0,0302	NAV3	-0,0229	NNMT	-0,0088
FXYD7	0,0299	ZEB2	-0,0229	EMCN	-0,0089
MFAP5	-0,0299	PTGS2	0,0228	DISC1	-0,0089
RAI14	-0,0297	PSPH	-0,0227	PLEKHB1	-0,0090
PTGER4	-0,0296	STC1	-0,0225	GPM6A	-0,0092
MX2	-0,0296	SPP1	0,0223	ADIPOQ	-0,0092
BCL6	-0,0296	FCGR2B	0,0223	SEMA3E	-0,0093
KCNS3	-0,0295	ASS1	0,0223	SFRP4	-0,0094
ATP6V1G2	0,0294	THBS4	0,0222	ACTA1	-0,0094
FEV	0,0294	HELLS	0,0221	SPP1	-0,0095
EFS	-0,0294	FAM70A	-0,0221	FUT9	-0,0095
FANCI	0,0293	CACNA2D3	0,0220	MT1F	-0,0096
CDKN3	0,0293	ADCY1	-0,0220	KLF4	-0,0098
IGH@ /// IGHG1 /// IGHG2 /// IGHM /// IGHV4-31	-0,0292	NID2	0,0220	MMP12	-0,0099
TG	0,0292	DNM3	-0,0220	GABRB1	-0,0100
SLC18A2	0,0291	TRAM2	0,0219	NDN	-0,0100
ECEL1	0,0290	NBLA00301	-0,0219	GATA3	-0,0100
TMEM158	-0,0289	PDGFRL	0,0219	IER3	-0,0101
GPR22	0,0289	ANXA1	0,0218	MAB21L2	-0,0102
TLE2	-0,0288	MT1X	0,0218	FMO1	-0,0102
CYR61	-0,0288	SCN3B	-0,0218	ISLR	-0,0103
ZFP36	-0,0286	NMU	0,0217	OGDH	-0,0103
XK	0,0286	CXCL9	0,0216	PLA2G2A	-0,0105
A2BP1	0,0286	TUBB4	-0,0214	GUCA1A	-0,0106
DNA2	0,0285	ARHGAP15	0,0212	PLSCR4	-0,0107

PRKCB	0,0284	ASPN	0,0212	COX7A1	-0,0109
RPRM	0,0284	RNASE4	0,0211	CRH	-0,0112
HLA-DRB4	-0,0283	AHNAK2	0,0210	SNF1LK	-0,0113
MAOB	-0,0283	PALM2-AKAP2	-0,0208	ABCB1 /// ABCB4	-0,0113
CD163	-0,0282	MC4R	-0,0208	ZKSCAN1	-0,0116
DLGAP5	0,0281	PCSK1	-0,0206	ZCCHC24	-0,0116
CTNND2	0,0281	AMPH	-0,0206	SNRPN	-0,0123
EFNB3	0,0280	CNR1	-0,0205	NRN1	-0,0123
PDLIM4	-0,0279	RBP4	0,0205	FAM153A /// FAM153B /// FAM153C	-0,0123
CDC45L	0,0279	CNTNAP2	-0,0204	TNS3	-0,0123
ADAMDEC1	0,0276	EFNB3	-0,0204	SORBS1	-0,0123
CXCR7	-0,0276	MAGEA2 /// MAGEA2B /// MAGEA6	-0,0203	CCL18	-0,0128
ZFPM2	0,0275	STMN4	-0,0202	ZEB2	-0,0129
BAI2	0,0274	RCAN2	-0,0202	ADCY2 /// LOC100133953	-0,0132
MX1	-0,0273	CXCL14	0,0201	CTAG1A /// CTAG1B	-0,0137
PTGS2	-0,0269	DPP6	-0,0201	EYA1	-0,0137
ABCB1	-0,0269	FGF9	0,0199	FGF9	-0,0137
IFITM1	-0,0269	CRYAB	-0,0199	ZBTB38	-0,0139
LY6H	0,0269	NAP1L2	-0,0197	PRAME	-0,0141
GJA1	-0,0269	RARRES2	0,0197	CDH4	-0,0142
BLM	0,0267	BAI2	-0,0196	DGKB	-0,0144
ST6GALNAC5	-0,0267	APOD	0,0196	KIAA0644	-0,0150
EFEMP1	-0,0265	SLC6A15	-0,0196	CRISPLD2	-0,0155
SLIT1	0,0265	ADAM22	-0,0195	MAGEB2	-0,0156
MKI67	0,0264	DYNC1I1	-0,0193	RIT2	-0,0161
IL10RA	-0,0264	EMCN	0,0193	PSPH	-0,0161
FLRT2	-0,0264	GAL	-0,0193	PDGFD	-0,0162
HBB	0,0264	OAS2	0,0192	CNR1	-0,0163
ALDH1A2	0,0262	NDN	0,0191	GPR22	-0,0164
CNN3	-0,0262	F3	0,0191	YIPF5	-0,0164
ABCB1 /// ABCB4	-0,0262	TNS3	0,0190	MAGI1	-0,0164
PCDH7	0,0261	ECEL1	-0,0189	PDGFC	-0,0165
CTGF	-0,0261	TOX3	-0,0189	KCNK3	-0,0165
COX7A1	-0,0260	ACTL6B	-0,0188	NKAIN1	-0,0166
SST	-0,0260	SV2B	-0,0188	PLP1	-0,0168
TCEAL2	0,0258	BHLHB3	-0,0186	HAND1	-0,0175
EVI2B	-0,0255	PDZD2	-0,0186	PTGFR	-0,0176
BST2	-0,0249	CTAG1A /// CTAG1B	-0,0186	FASN	-0,0177
HLA-DQA1 /// HLA-DQA2	-0,0248	OGN	0,0185	LOC100127887 /// SYT2	-0,0178
ELAVL2	0,0247	ANKRD57	-0,0185	AHR	-0,0178
SLC2A14 /// SLC2A3	-0,0247	LAMA2	0,0183	CD36	-0,0180
ENDOG	0,0247	EGR3	0,0183	PRELP	-0,0182
CAV1	-0,0246	NPFF	-0,0183	ZFP36	-0,0182
FXYD5	-0,0241	HBA1 /// HBA2	0,0183	SHANK2	-0,0183
GOLM1	0,0241	IFITM1	0,0182	DIRAS3	-0,0185
CCL8	-0,0241	ATF3	-0,0181	STAT3	-0,0186
DNASE1L3	-0,0239	NHLH2	-0,0179	ANXA1	-0,0187
LEF1	-0,0239	GLT8D2	0,0178	LOXL1	-0,0188
CDCA4	0,0239	TCEAL2	0,0178	CFH	-0,0188
PDE8B	-0,0238	LOC100134306 /// MYT1L	-0,0177	TFAP2B	-0,0189
RCAN2	-0,0236	LOC157627	0,0177	HOXC10	-0,0192
IFI27	-0,0236	EPB41L2	0,0175	FGF13	-0,0192
SERPINB9	-0,0233	THBD	0,0175	CFD	-0,0195
ECT2	0,0233	SST	0,0175	ACTL6B	-0,0198
CFD	-0,0231	LGI1	-0,0175	ADD2	-0,0203
STC1	-0,0231	DISC1	-0,0174	NPTX2	-0,0205
MEX3D	0,0230	MOXD1	0,0173	PALM2-AKAP2	-0,0207
HBG1 /// HBG2	0,0230	SLC38A1	-0,0171	FEV	-0,0213
CENPE	0,0228	GULP1	-0,0171	SH3GL3	-0,0214
HJURP	0,0228	SLC18A2	-0,0170	MAL	-0,0215
HCLS1	-0,0227	PLP1	-0,0168	ENOX1	-0,0216
FASN	0,0227	EMP2	0,0167	ABCA8	-0,0218
CNTN1	0,0223	WWTR1	0,0165	DPYSL4	-0,0218
NPFF	0,0223	SCN7A	-0,0165	C4A /// C4B	-0,0220
ABLIM3	-0,0222	CRISPLD2	0,0164	COBL	-0,0220
PEG3 /// ZIM2	0,0220	HAND1	-0,0163	SV2B	-0,0220
MAGEL2	0,0219	FOSB	0,0163	EEF1A2	-0,0221
LOC653188 /// SMA4 /// SMA5	0,0219	DPYSL4	-0,0162	CXCL10	-0,0222
GFRA3	-0,0216	CHST1	0,0162	BCL6	-0,0222
IFIT3	-0,0216	DUSP5	-0,0159	CSAG2 /// CSAG3	-0,0224
ASS1	-0,0216	EMP1	0,0159	LTBP2	-0,0224
MEIS2	0,0215	DHRS3	0,0158	DBH	-0,0226
STAT3	-0,0212	MYO10	0,0157	LOC100134306 /// MYT1L	-0,0227
HELLS	0,0211	FABP6	-0,0157	PIK3R1	-0,0228
SYT17	0,0209	UTS2	-0,0157	IL1R1	-0,0229
OSBPL3	0,0208	SLC17A6	-0,0156	EFEMP1	-0,0237
GALNT6	0,0208	RGS7	-0,0152	RAC3	-0,0237
LTBP2	-0,0207	IGFBP6	0,0149	NGFR	-0,0238
FOS	-0,0205	CDH19	-0,0149	GATM	-0,0239
CUX2	0,0203	ATP10D	0,0149	LUM	-0,0239
SNX10	-0,0202	PCSK2	-0,0149	CFH /// CFHR1	-0,0240
KIAA0644	0,0202	PENK	-0,0148	APBA2	-0,0244
MCM10	0,0200	TNXA /// TNXB	0,0147	AKAP7	-0,0245
SLC19A2	-0,0199	SEMA3C	-0,0147	STMN4	-0,0247

CXorf57	0,0198	PTX3	0,0147	KIF5C	-0,0247
FAM5C	0,0197	BAG3	0,0145	CXCL12	-0,0250
PLXNA2	0,0197	FGF1	-0,0145	METTL7A	-0,0251
SNX7	-0,0197	TMEM158	0,0143	LOC284244	-0,0252
ATAD2	0,0196	ITIH5	0,0143	MRC1 /// MRC1L1	-0,0254
FAM153A /// FAM153B /// FAM153C	0,0196	SLC2A14 /// SLC2A3	-0,0143	DDC	-0,0256
CXCL10	-0,0195	ISG15	-0,0143	CYR61	-0,0259
HBA1 /// HBA2	0,0193	CTNNAL1	0,0141	CSGALNACT1	-0,0260
IGK@ /// IGKC	-0,0193	NT5DC2	0,0141	HIST1H2AC	-0,0260
CCDC68	0,0191	CDH1	-0,0141	ADH1B	-0,0262
S100A8	-0,0191	ENDOD1	-0,0138	EPB41L3	-0,0263
CHD5	0,0190	GRAMD3	0,0137	CHRNA3	-0,0265
ALDOC	-0,0190	MAB21L1	0,0135	KIF1A	-0,0267
PTGFR	-0,0190	MT1F	0,0134	RARRES2	-0,0267
EPB41L3	-0,0183	ZNF536	-0,0134	TAC1	-0,0268
LOC100127887 /// SYT2	0,0182	SLC6A2	-0,0134	CDH12	-0,0268
PAWR	-0,0181	DDC	-0,0133	SLC17A6	-0,0268
RAB3B	0,0179	MT1E	0,0132	EFNB2	-0,0269
SEMA5A	-0,0178	OLFML2A	-0,0131	ZNF365	-0,0272
LTB	0,0178	GPM6A	-0,0130	CDH19	-0,0272
KIF1A	0,0177	FMO3	0,0129	NAP1L2	-0,0273
CPEB1	-0,0177	NETO2	0,0129	PLXNA2	-0,0274
LRBA	0,0177	KCNS3	-0,0129	HRASLS3	-0,0274
SHANK2	0,0176	BCL6	0,0128	MYT1	-0,0277
SMC4	0,0176	IL8	0,0128	A2BP1	-0,0278
P2RX5	0,0173	PLEKHB1	-0,0128	ZNF804A	-0,0278
DIXDC1	-0,0173	S100B	-0,0127	ERC1	-0,0284
EFNB2	0,0172	NELL2	-0,0127	COL14A1	-0,0285
FGF9	0,0170	ALDH1A1	0,0126	KCNQ2	-0,0291
ASCL1	0,0170	RIMBP2	-0,0125	CD163	-0,0295
POSTN	0,0168	AHNAK	0,0124	DAB2	-0,0295
COPG2IT1	0,0166	EFNA5	0,0124	PRNP	-0,0297
SERPINE1	-0,0165	SOX9	-0,0122	S100A6	-0,0298
ADIPOQ	-0,0164	ZFP36	0,0122	SCN3A	-0,0299
RSRC1	0,0163	MAL	0,0121	TUBB2A /// TUBB2B	-0,0300
ISG15	-0,0162	NTRK3	0,0121	LY96	-0,0302
PRPH	0,0162	GPR177	-0,0120	RASSF2	-0,0303
SERPINA5	-0,0161	CEBPB	0,0120	APOD	-0,0309
MC4R	0,0160	GABRP	-0,0119	RPRM	-0,0309
SNRPN	0,0160	TNXB	0,0119	FGL2	-0,0311
NTRK3	0,0159	CRLF1	0,0119	MC4R	-0,0317
CALB1	0,0157	TWIST1	-0,0119	ZNF91	-0,0318
CTSH	-0,0157	THBS1	0,0119	CRYAB	-0,0320
IGF2	-0,0152	C10orf116	0,0117	HSPA12A	-0,0325
ATP8A2	0,0152	GLDC	0,0116	CTSZ	-0,0326
HSPA6	-0,0151	PRND	-0,0116	CCL2	-0,0326
SLITRK5	-0,0151	XK	0,0115	MAGEA9 /// MAGEA9B	-0,0334
CRABP1	-0,0150	AHR	0,0114	CPEB1	-0,0334
PTTG1	0,0149	TLE2	-0,0113	RALYL	-0,0337
EIF2S3	0,0146	HEPH	0,0113	HTR2C	-0,0340
CCL18	0,0145	MYOT	0,0113	GRIA2	-0,0344
NELL1	0,0143	SOX11	-0,0112	SLC6A2	-0,0344
MXRA5	-0,0143	IL7R	0,0112	MX2	-0,0345
GABRB1	0,0142	CHODL	-0,0111	MAGEA2 /// MAGEA2B /// MAGEA6	-0,0345
EPHA5	0,0142	PMP2	-0,0109	PLD3	-0,0350
hCG_1644608 /// SET	0,0138	SNX10	-0,0108	MAGEL2	-0,0353
AKAP7	-0,0138	ALDH1A3	0,0107	ELAVL2	-0,0356
C7orf16	0,0137	KCNQ2	-0,0107	MAGEA11	-0,0357
ODZ3	0,0136	HSD3B2	-0,0107	MAPT	-0,0369
RPH3A	0,0135	MAGEA10	0,0106	ARHGAP15	-0,0380
REEP1	0,0133	COBL	0,0106	IL10RA	-0,0381
GPM6A	-0,0129	GATA3	0,0105	TG	-0,0382
RBP4	-0,0128	P2RY14	0,0104	FBLN5	-0,0384
PLD3	0,0123	IFIT3	-0,0103	RCAN2	-0,0385
ADCY2 /// LOC100133953	0,0122	CFI	0,0103	DNASE1L3	-0,0390
DNM3	0,0121	SDC4	-0,0102	FABP4	-0,0396
GAL	0,0121	FAM107A	0,0102	MAGEA5	-0,0398
ATP10D	-0,0120	GNG3	-0,0101	ADCY1	-0,0400
GLDC	0,0119	WFDC1	-0,0101	GATA2	-0,0405
IGK@ /// IGKC /// IGKV3-20 /// IGKV3D-11 /// IGKV3D-15	-0,0118	MAGEL2	0,0100	HLA-DRB4	-0,0406
CCL11	-0,0118	DLK1	-0,0100	ODZ3	-0,0408
IL8	-0,0117	DNASE1L3	0,0099	XK	-0,0408
CCL19	0,0115	CCL11	0,0099	IFI44L	-0,0410
PDAP1	0,0114	CRABP1	-0,0099	BST2	-0,0416
SCARB2	-0,0113	GATA2	0,0098	SERPINA5	-0,0418
NMU	0,0112	GATA6	0,0098	LRRN3	-0,0427
PRSS3	0,0111	ADD2	0,0097	ZFHX3	-0,0431
CDH12	0,0110	LIF	0,0096	OAS2	-0,0433
NPTX2	-0,0110	EGFL8 /// PPT2	-0,0096	CD302	-0,0433
GUCA1A	-0,0109	HAS2	0,0095	ITM2A	-0,0438
AK5	-0,0109	LOC284244	0,0094	C1orf165	-0,0441
BCL2A1	-0,0108	CHRNA3	-0,0094	MS4A4A	-0,0441
MAGEA6	-0,0107	MAGEA11	0,0094	ALDH1A1	-0,0444
SULF1	0,0107	NR4A2	-0,0094	MMP9	-0,0444

EYA4	0,0105	GNG12	-0,0094	CNTNAP2	-0,0445
PTPRC	-0,0104	EFNB2	-0,0091	DCN	-0,0446
MAGEA10	0,0103	MAGEA9 /// MAGEA9B	-0,0091	IFIT1	-0,0448
HOXC10	-0,0102	ISL1	0,0089	AMPH	-0,0449
GZMB	-0,0102	LRRN3	0,0089	ATP1A3	-0,0452
DYNC1I1	-0,0101	KAL1	-0,0088	MS4A1	-0,0453
LYZ	-0,0101	NELL1	-0,0088	FAM115A /// FAM115B	-0,0454
ADM	-0,0101	SERPINA5	0,0088	EPHA5	-0,0455
POPDC3	-0,0100	ANGPTL7	0,0087	RAB3B	-0,0456
SPP1	-0,0100	IFI27	0,0087	EVI2B	-0,0457
DGKB	0,0100	TNFAIP6	0,0086	TCEAL2	-0,0459
HTR2C	0,0099	MX2	0,0085	RIMS3	-0,0462
FAM69A	0,0099	PEG3	-0,0085	GNAO1	-0,0462
PCSK2	-0,0098	NKAIN1	-0,0084	ADRB2	-0,0463
GAS1	-0,0098	SLC4A8	0,0083	NEBL	-0,0464
AMPH	0,0097	RIMS3	-0,0080	TCL1A	-0,0466
GPX1	-0,0096	MAFF	-0,0080	MAGEA10	-0,0466
RALYL	0,0095	TBC1D30	0,0079	VSNL1	-0,0470
G0S2	-0,0094	HOXC4 /// HOXC6	0,0078	RALGPS1	-0,0470
LOC100133662 /// RPS4Y1	0,0094	CRH	-0,0078	AHNAK2	-0,0471
GABRP	0,0093	GPC3	0,0076	C1R	-0,0474
CALB2	0,0089	ABCG2	0,0074	CHD5	-0,0476
KIF3B	-0,0089	HSPA12A	-0,0073	DPP6	-0,0476
PPP1R1A	-0,0088	IFIT1	0,0073	MS4A6A	-0,0485
CCL3 /// CCL3L1 /// CCL3L3 /// LOC728830	-0,0086	HBG1 /// HBG2	-0,0073	IGH@ /// IGHA1 /// IGHA2 /// IGHV3OR16-13 /// LOC100126583	-0,0489
TTTY15	0,0085	MX1	0,0073	LOC100133233 /// TRAF3IP3	-0,0494
LCK	0,0085	ERBB2	0,0072	CXorf57	-0,0499
IGKC	-0,0085	SORBS1	0,0072	ALDOC	-0,0500
DUSP4	-0,0085	NGFR	0,0072	ISG15	-0,0502
IGL@ /// IGLC2 /// IGLV2-14	-0,0084	ENOX1	0,0072	IGL@	-0,0502
LOC100130100	-0,0084	DAAM2	0,0071	AKR1C2	-0,0503
XIST	-0,0084	VIP	-0,0070	NPFF	-0,0509
CSAG2 /// CSAG3	-0,0083	NDUFS7	0,0069	ATP6V1G2	-0,0509
TWIST1	-0,0080	PLXNA2	-0,0069	IFI27	-0,0510
LOC100130216 /// USP9Y	0,0078	PAFAH1B3	0,0068	REEP1	-0,0518
LRRC37A /// LRRC37A2	0,0078	PLLP	-0,0067	SLC18A1	-0,0521
NAV3	-0,0078	ADAMTS8	0,0066	SCN3B	-0,0523
AMIGO2	-0,0078	EDNRB	0,0066	IGK@ /// IGKC /// IGKV3-20 /// IGKV3D-11 /// IGKV3D-15	-0,0526
CILP	-0,0077	SLIT1	0,0066	CAMK2B	-0,0526
NDN	-0,0076	GFRA3	-0,0065	CNTN1	-0,0527
EIF1AY	0,0076	ITGB4	-0,0065	HCLS1	-0,0532
NEBL	0,0076	GATM	0,0064	PCDH7	-0,0532
CD48	-0,0074	S100A6	-0,0064	AK5	-0,0532
CTSZ	-0,0074	CXADR	-0,0062	FAM69A	-0,0534
YIPF5	0,0074	PON2	0,0062	IFITM1	-0,0541
FBLN1	-0,0073	C1orf165	0,0062	SYT17	-0,0543
ERBB4	0,0072	FAM114A1	0,0061	PTGER4	-0,0544
AMFR	-0,0072	MIA	-0,0061	CTNND2	-0,0546
ADRB2	0,0072	LPL	0,0060	SLC18A2	-0,0547
CCR7	0,0072	CALB2	0,0059	PTGDS	-0,0552
CD3D	0,0072	PRKCB	0,0059	POU2AF1	-0,0552
IL7R	0,0071	CNTN1	-0,0059	ACHE	-0,0553
ACHE	0,0071	RPRM	-0,0058	CXCL9	-0,0557
ASPN	-0,0071	MEG3	-0,0055	AMIGO2	-0,0561
EIF3I	-0,0070	SNF1LK	-0,0055	COPG2IT1	-0,0562
CXCL9	0,0069	ETV1	-0,0055	SERPINB9	-0,0564
RAMP3	0,0069	CCDC68	0,0055	ADAMDEC1	-0,0565
NDUFS7	0,0068	TGFBR3	-0,0054	DENND2D	-0,0566
ZBTB38	-0,0067	NPTX2	0,0053	IGKC	-0,0570
MPHOSPH8	-0,0067	CUX2	-0,0049	GNG3	-0,0572
MAGEA3	-0,0067	SLCO1C1	0,0048	LEF1	-0,0574
HSD3B2	-0,0067	APBA2	-0,0048	PPP1R1A	-0,0575
CCL21	0,0066	COCH	0,0048	LOC652493	-0,0575
CREB5	-0,0063	RXRG	0,0047	HOXC4 /// HOXC6	-0,0579
MAGEA11	0,0061	TM4SF1	0,0047	BAI2	-0,0582
SV2B	-0,0060	ZFHX4	-0,0047	LYZ	-0,0584
ZNF652	0,0059	SIX3	0,0047	FAM5C	-0,0584
IGL@	-0,0058	FNDC3B	0,0047	GFRA3	-0,0585
PDE10A	-0,0057	SEMA3B	-0,0047	CHST1	-0,0598
CDH18	0,0056	FZD2	0,0047	PRPH	-0,0607
DENND2D	0,0054	FCGBP /// LOC100133944	-0,0047	SNX10	-0,0608
PTPRK	-0,0054	COL21A1	-0,0046	ATP8A2	-0,0610
PMAIP1	-0,0054	ADRB2	0,0046	ZFHX4	-0,0618
FABP4	-0,0054	MAGI1	0,0043	GABBR1 /// UBD	-0,0620
ERC1	0,0053	COX7A1	0,0043	TH	-0,0624
LOXL1	0,0051	CAPN6	0,0043	NPY	-0,0630
OAS2	-0,0051	GAS7	-0,0041	PLAC8	-0,0630
TCL1A	-0,0051	IER3	-0,0040	PEG3	-0,0637
PEG3	0,0051	FBP1	0,0038	FXYD5	-0,0646
IGH@ /// IGHA1 /// IGHA2 /// IGHV3OR16-13 /// LOC100126583	0,0051	PCP4	-0,0037	EFNB3	-0,0654
NPY	-0,0050	TSPAN8	0,0035	LCK	-0,0657
CD52	0,0048	KCNE4	0,0034	HLA-DRA	-0,0659
CTAG1A /// CTAG1B	0,0048	CELSR3 /// SLC26A6	0,0033	IL7R	-0,0662

ZKSCAN1	0,0047	ERBB3	-0,0032	ECEL1	-0,0666
BMP7	0,0047	PLCE1	-0,0031	MAGEA12	-0,0670
ZFHX4	0,0046	GPM6B	0,0030	MAB21L1	-0,0672
CALY	0,0044	DKFZP586H2123	0,0030	C7	-0,0673
TRBC1 /// TRBC2 /// TRBV19	0,0038	PTPRZ1	-0,0030	PEG3 /// ZIM2	-0,0678
SPOCK2	-0,0037	FGF13	-0,0029	TRIM22	-0,0680
ENTPD4 /// LOXL2	0,0037	PAWR	-0,0029	CRIP2	-0,0683
JARID1D	0,0035	PLEKHA4	-0,0029	CD3D	-0,0688
MAGEB2	0,0034	ADAMTS1	-0,0029	MX1	-0,0688
TRBC1	-0,0031	MPZ	-0,0028	C1S	-0,0690
LOC100133233 /// TRAF3IP3	0,0030	MATN2	-0,0028	CCR7	-0,0692
HOXC4 /// HOXC6	0,0029	HRASLS3	0,0028	ALDH1A2	-0,0696
PCP4	-0,0028	STARD13	-0,0027	RAMP3	-0,0697
SEMA3E	0,0027	ASPA	-0,0026	FAIM3	-0,0700
MAGEA5	0,0026	C7orf16	-0,0026	IGL@ /// IGLC2 /// IGLV2-14	-0,0704
IGHM	0,0026	MYT1	-0,0025	RGS7	-0,0704
NHLH2	0,0026	MT1M	0,0024	FAM70A	-0,0707
DDX3Y	0,0026	HTR2C	0,0023	TUBB4	-0,0711
OGDH	0,0025	ADM	0,0021	PTPRC	-0,0712
PSPH	-0,0024	MICALL2	-0,0019	MAGEA6	-0,0715
SLC17A6	0,0023	BST2	-0,0018	LY6H	-0,0725
ZNF91	0,0023	ST6GALNAC2	-0,0018	DNM3	-0,0730
HTR3A	0,0023	MAGEA5	-0,0017	OSBPL3	-0,0736
ACTA1	0,0022	CPEB1	0,0016	CD52	-0,0737
SV2C	-0,0022	SOX10	-0,0016	SELL	-0,0743
AKR1C2	0,0021	DBH	0,0016	C7orf16	-0,0747
FAM70A	0,0021	MEX3D	0,0015	NAV3	-0,0752
MFAP4	-0,0020	ATP1A2	-0,0014	CTSH	-0,0759
HIST1H2AC	0,0020	ATP8A2	-0,0014	HLA-DMA	-0,0768
FAIM3	-0,0020	SASH1	0,0014	MAGEA3	-0,0769
MAGEA2 /// MAGEA2B /// MAGEA6	-0,0019	MAOB	0,0014	CD48	-0,0793
CRIP2	0,0018	NOL4	-0,0013	DYNC1I1	-0,0795
LOC348162 /// LOC642799 /// LOC729602	-0,0017	LDLR	0,0013	IGHM	-0,0798
PLAC8	0,0014	VGLL3	0,0013	ZFPM2	-0,0808
LOC652493	-0,0014	RHBDF1	-0,0013	LOC100130100	-0,0818
DLK1	-0,0014	MAGEB2	-0,0011	FXYD7	-0,0826
PRND	0,0012	SERPINA3	-0,0011	CACNA2D3	-0,0826
PIK3R1	0,0010	HMG20B	0,0011	LTB	-0,0835
CSGALNACT1	0,0008	SERPINE1	0,0010	PRKCB	-0,0844
GATA6	-0,0008	PDGFA	-0,0010	DARC	-0,0844
RIT2	0,0007	FXYD1	-0,0010	CALY	-0,0854
MAGEA12	-0,0007	C8orf4	0,0009	TRBC1 /// TRBC2 /// TRBV19	-0,0881
PCDH8	0,0006	GDAP1L1	-0,0009	CCL21	-0,0883
GABBR1 /// UBD	-0,0006	ZFHX3	0,0009	IGK@ /// IGKC	-0,0896
FBP1	-0,0006	AKR1C2	-0,0008	ABLIM3	-0,0913
MS4A1	-0,0005	AQP1	0,0007	SPOCK2	-0,0928
MAGEA9 /// MAGEA9B	0,0004	ITGA6	0,0007	HLA-DQA1 /// HLA-DQA2	-0,0956
POU2AF1	-0,0003	TG	0,0007	C3	-0,0957
HMOX1	0,0003	COL9A3	0,0005	TRBC1	-0,0966
MAGEA4	-0,0002	PRPH	0,0005	NTRK1	-0,0977
SELL	-0,0002	GPR126	0,0004	IL7	-0,1004
CD36	0,0001	CX3CR1	0,0002	IGH@ /// IGHG1 /// IGHG2 /// IGHM /// IGHV4-31	-0,1007
CHODL	0,0001	FOSL2	-0,0002	JUP /// KRT19	-0,1071
ZNF365	0,0000	HSPA6	0,0002	CCL19	-0,1245
CNR1	0,0000	RALGPS1	-0,0001	XIST	-0,1336

**Common variables (present in both data sets)**
A2BP1
ABCA8
ABCB1
ADAMDEC1
ADAMTS1
ADRB2
AHNAK2
AKAP7
AKR1C2
ALDH1A2
ALDOC
AMIGO2
AMPH
ANGPTL7
APOD
ARHGAP15
ASCL1
ASPA
ASPM
ASPN
ASS1
ATF3
ATP6V1G2
BAG3
BIRC5
BUB1B
C1S
C3

C4A /// C4B
C7
C7orf16
CACNA2D3
CALB1
CALY
CCL18
CCL19
CCL2
CCL21
CCNB1
CCNB2
CD163
CD48
CD52
CDC20
CDH18
CDH19
CENPF
CFH
CGA
CKS2
CNTNAP2
COBL
COPG2IT1
COX7A1
CPEB1
CRH
CRISPLD2
CRYAB
CTGF
CTNNAL1
CUX2
CXCL14
CXCL2
CXCL9
CYR61
DBH
DCN
DDC
DDX3Y
DIRAS3
DLGAP5
DLK1
DNASE1L3
DPP6
DTL
DYNC1I1
EFEMP1
EIF1AY
EPB41L3
EPB41L4B
EPHA5
EYA1
F12
FAIM3
FAM64A
FAM69A
FAM70A
FGF13
FOS
FOSB
FOXM1
FUT9
FZD2
GABBR1 /// UBD
GABRB1
GAL
GINS2
GJA1
GPM6B
GPR22
HBA1 /// HBA2
HBB
HBG1 /// HBG2
HLA-DMA
HLA-DQA1 /// HLA-DQA2
HLA-DRA
HLA-DRB4
HOXC4 /// HOXC6
HRASLS3
HSD3B2
HSPA6
IFI44L

IGF2
IGH@ /// IGHA1 /// IGHA2 /// IGHV3OR16-13 /// LOC100126583
IGH@ /// IGHG1 /// IGHG2 /// IGHM /// IGHV4-31
IGHM
IGK@ /// IGKC
IGL@ /// IGLC2 /// IGLV2-14
IL7
IL8
INSM1
JARID1D
JUP /// KRT19
KCNK3
KIF15
KIF20A
KIF2C
KIF4A
LGI1
LOC100133662 /// RPS4Y1
LYZ
MAB21L1
MAB21L2
MAGEA10
MAGEA11
MAGEA3
MAGEA4
MAGEA6
MAGEA9 /// MAGEA9B
MAL
MAOB
MATN2
MCM2
MEIS2
MELK
MET
MGC39900 /// TMSL8
MIA
MLF1IP
MMP12
MMP9
MT1E
MT1X
MX1
NAV3
NCAN
NDN
NEBL
NHLH2
NMU
NOL4
NPY
NR4A2
NT5DC2
NTRK1
NUSAP1
ODZ3
OLFML2A
P2RX5
PBK
PDGFRA
PLAC8
PLAT
PLP1
PMP2
POSTN
POU2AF1
PRAME
PRC1
PRKCB
PRNP
PTGDS
PTTG1
RAB3B
RALYL
RAMP3
RBP4
RCAN2
RELN
RGS7
RRM2
S100A8
S100B
SCN3A
SELL
SEMA3B

SERPINA3
SERPINA5
SERPINF1
SIX3
SLC18A1
SLC18A2
SMC4
SNF1LK
SOX10
SPOCK2
SPP1
SRPX
SST
ST6GALNAC2
ST6GALNAC5
STC1
SV2B
SV2C
TAC1
TCL1A
TFAP2B
TG
TH
THBS4
TNS3
TOP2A
TPX2
TRBC1
TRBC1 /// TRBC2 /// TRBV19
TRIP13
TWIST1
TYMS
UBE2C
VIP
VSNL1
XIST
ZFPM2
ZNF804A
ZNF91
ZWINT

Column 1-6: Variables (genes/probe-sets) and their PCA loadings for Principal components 1, 2, and 3 (PC1, PC2, PC3) in data-set 1 and 2 (De Preter and McArdle/Wilzén respectively). Common variables: Genes/probe-sets that were present in the PCA analysis of both data-sets.
