# Supplementary figures and images for: A 6-gene signature identifies four molecular subgroups of neuroblastoma
Source: Cancer Cell Int. 2011 Apr 14;11:9. doi: 10.1186/1475-2867-11-9 (PMC3095533; doi:10.1186/1475-2867-11-9)

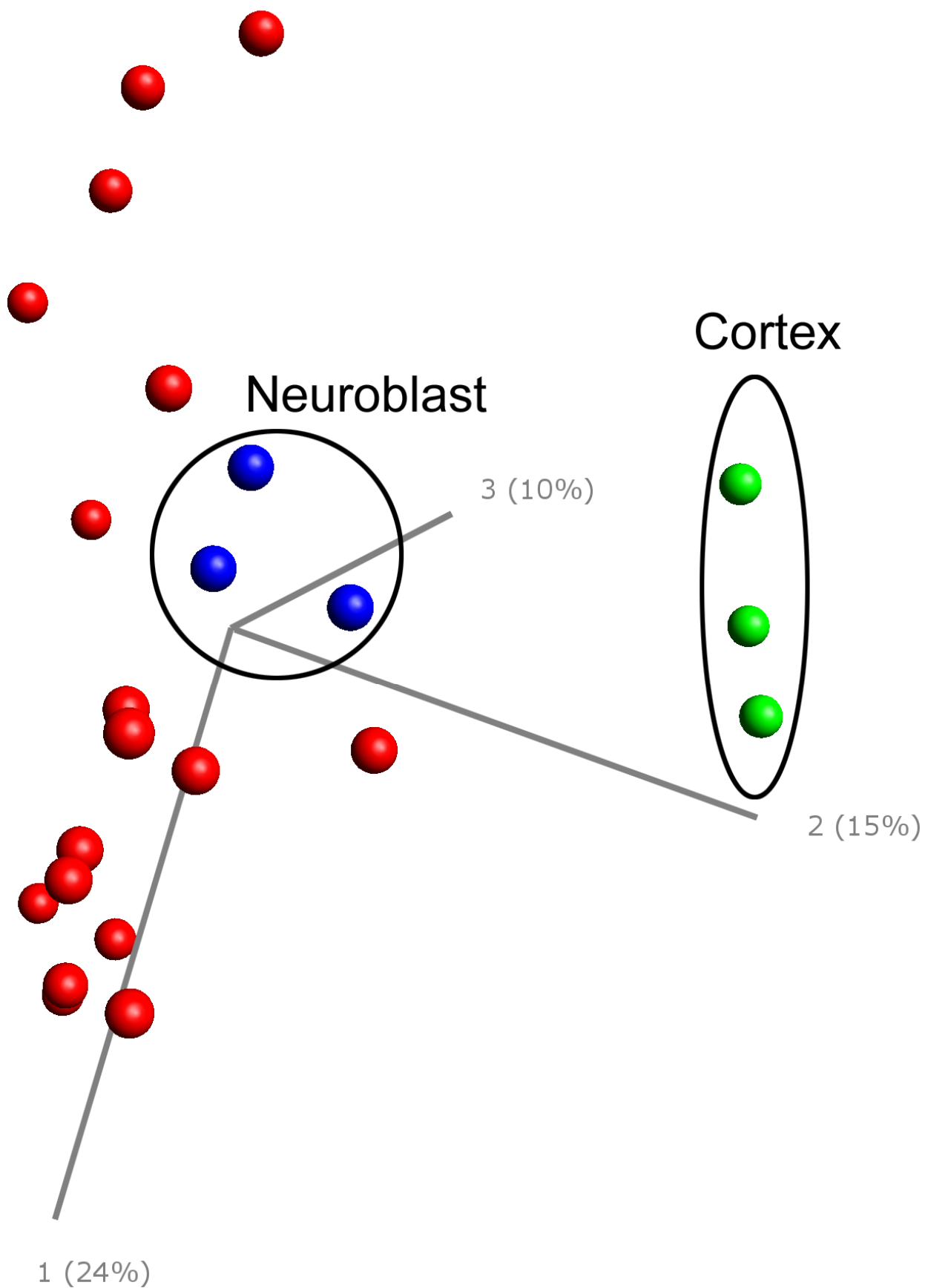

Supplement: Additional file 2 — PCA of cortex, neuroblast and NB samples. Unfiltered Principal Components Analysis (PCA) of the De Preter data set (7438 variables, 23 samples. Colour codes of spheres: Red = neuroblastoma tumour specimens; Blue = Neuroblasts; Green = Cortex. [file 1475-2867-11-9-S2.PDF]

### ALK

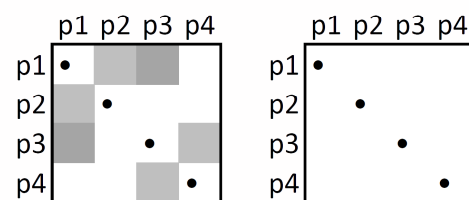

### BIRC5

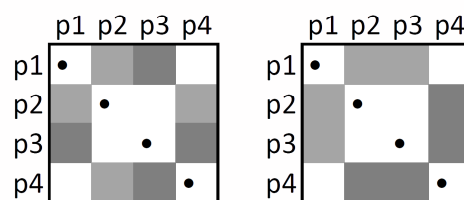

### CCND1

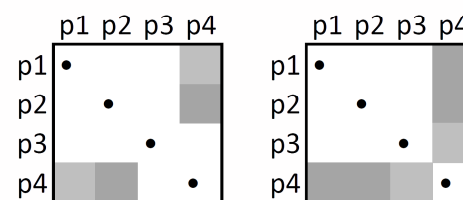

### MYCN

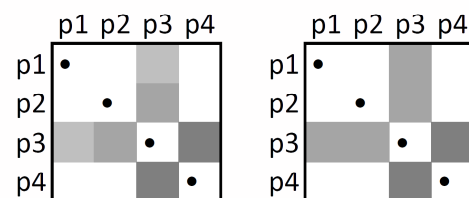

### NTRK1

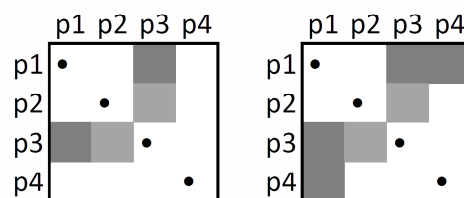

### PHOX2B

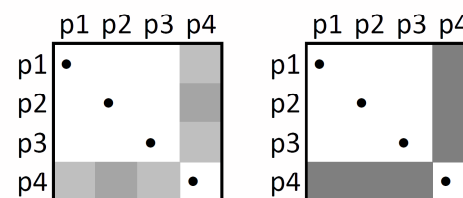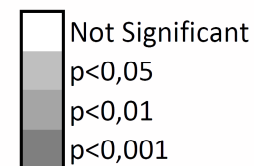

Supplement: Additional file 5 — Multiple comparisons by Post hoc test (Tukey). Gene expression of ALK, BIRC5, CCND1, MYCN, NTRK1, and PHOX2B in PCA clusters p1-p4 of the two data sets De Preter (left) and McArdle/Wilzén (right) was analysed by a Post-hoc test (Tukey). Significance level is marked by a grey colour scale. [file 1475-2867-11-9-S5.PDF]

A

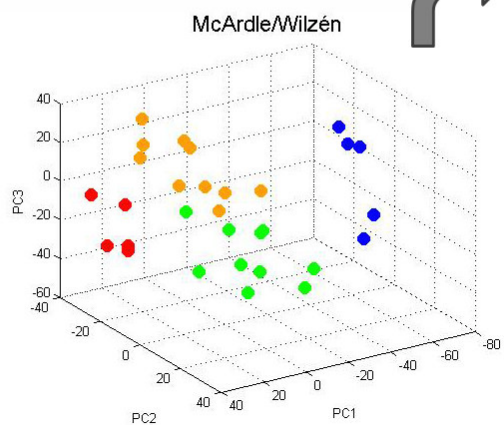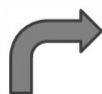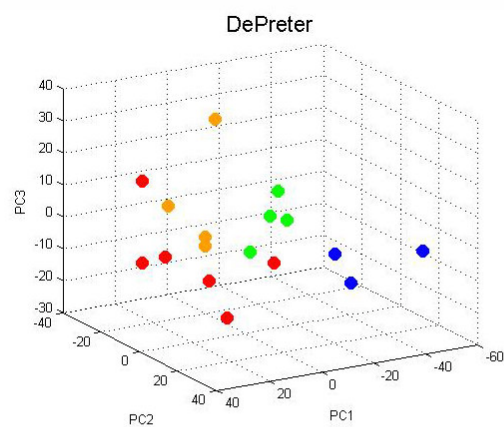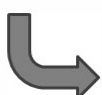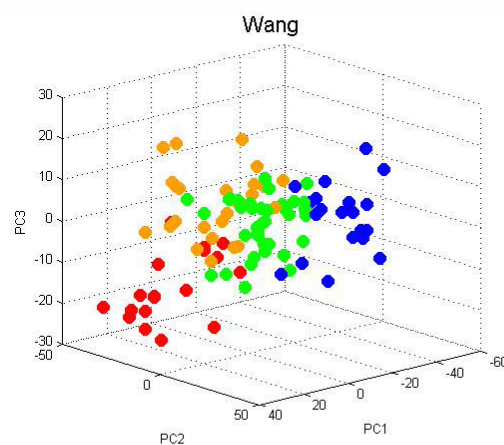

B

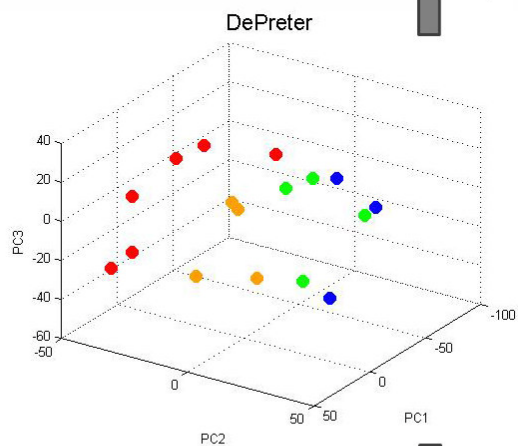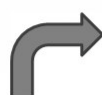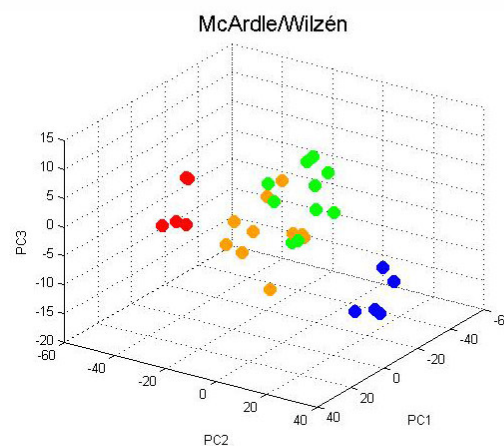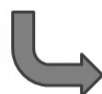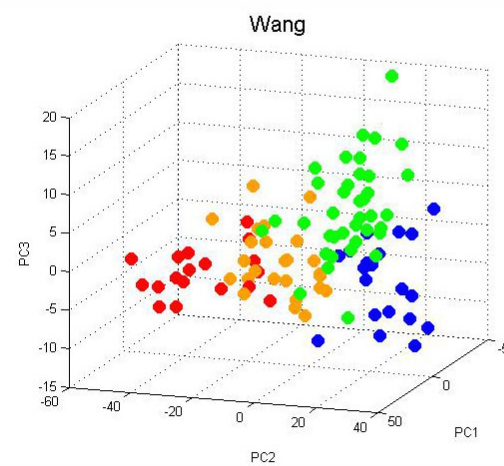

Supplement: Additional file 7 — PCA validation of p- and h-groups using unfiltered expression data. Principal Components Analysis (PCA) of unfiltered global expression data (4728 genes) from three data sets (De Preter, McArdle/Wilzén, and Wang). A. PCA plotted by loadings generated from the McArdle/Wilzén data set. B. PCA plotted by loadings generated from the De Preter data set. Cases (spheres) are coloured by their group assignments: Green = p1/h1, Orange = p2/h2, Red = p3/h3, Blue = p4/h4. [file 1475-2867-11-9-S7.PDF]

De Preter data set

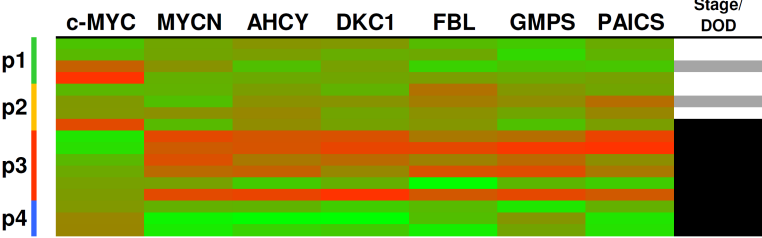

McArdle/Wilzén data set

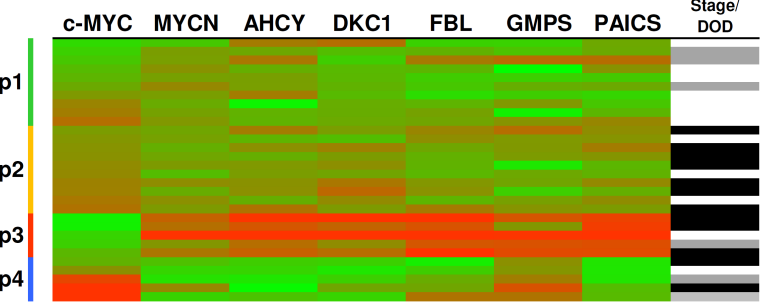

Wang data set

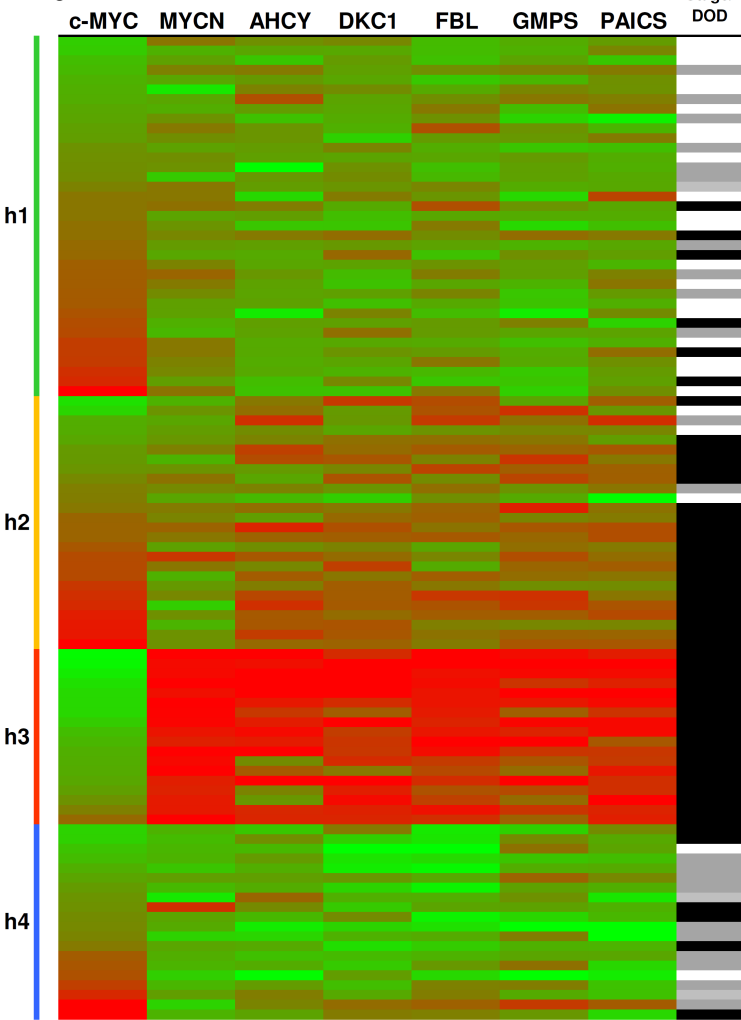

Supplement: Additional file 8 — Expression heat map of MYCN, c-MYC and MYCN/c-MYC downstream targets. The two test data sets De Preter (n = 17, Upper left panel) and McArdle/Wilzén (n = 30, lower left panel) are divided into four PCA clusters (p1-p4), and the verification data set Wang (n = 102, right panel) is divided into four hierarchical clusters (h1-h4). The heat-map colour scale is based on standard deviations (sd) and ranges from +2 sd (red) to -2 sd (green). Status of prognostic factors is shown by black and white squares to the right of each panel. Stage/DOD: Black = INSS stage 4 or dead of disease, Dark grey = INSS stage 3, White = Low INSS stage (stage 1 or 2) and alive, Light grey = Not determined. [file 1475-2867-11-9-S8.PDF]
