# Supplementary material for: A 6-gene signature identifies four molecular subgroups of neuroblastoma
Source: Cancer Cell Int. 2011 Apr 14;11:9. doi: 10.1186/1475-2867-11-9 (PMC3095533; doi:10.1186/1475-2867-11-9)

Step 1: Subgroup discovery by PCA (data sets 1 & 2)

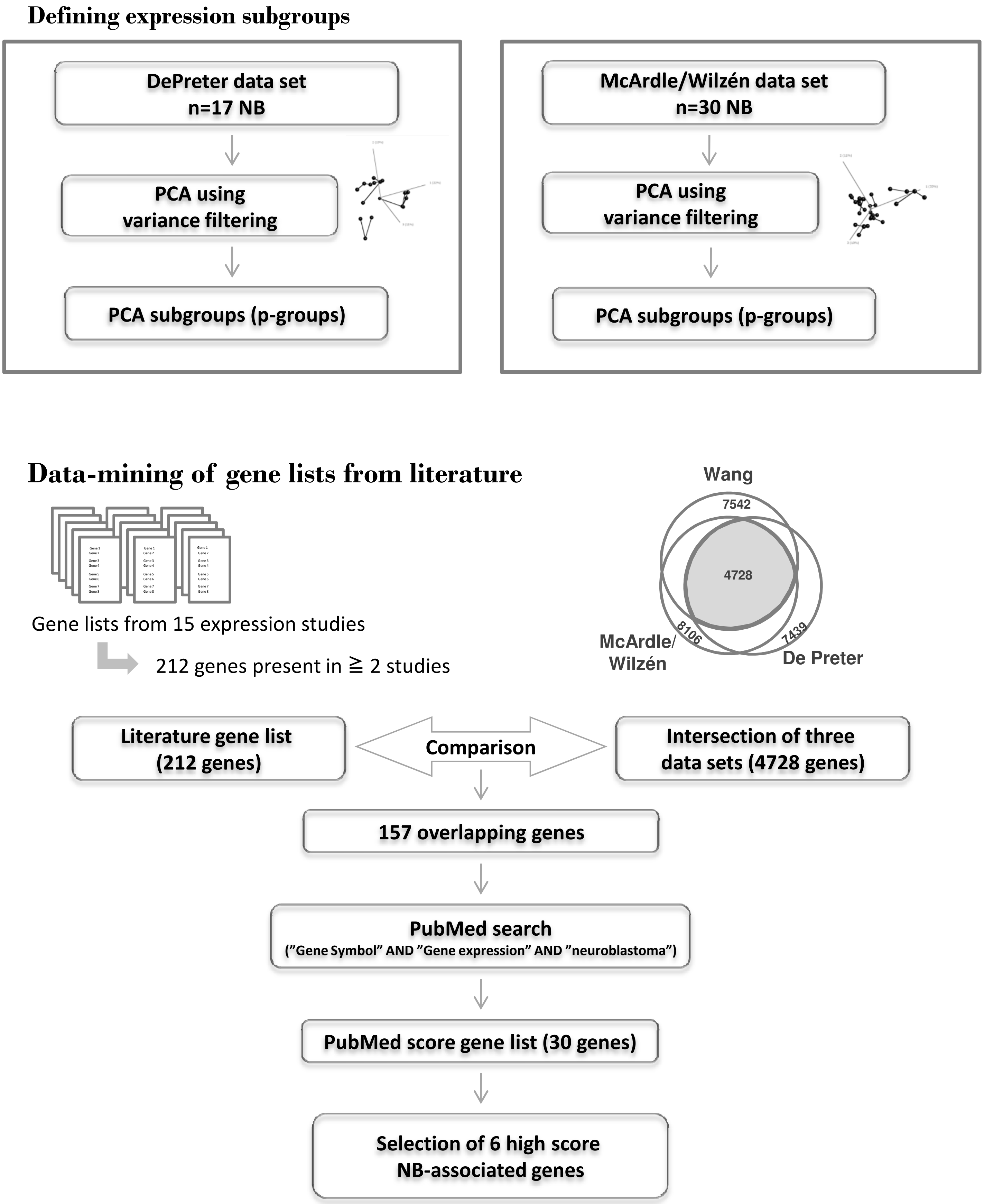

Step 2: Verification by hierachical clustering & PCA (data set 3)

Defining a classifier gene set

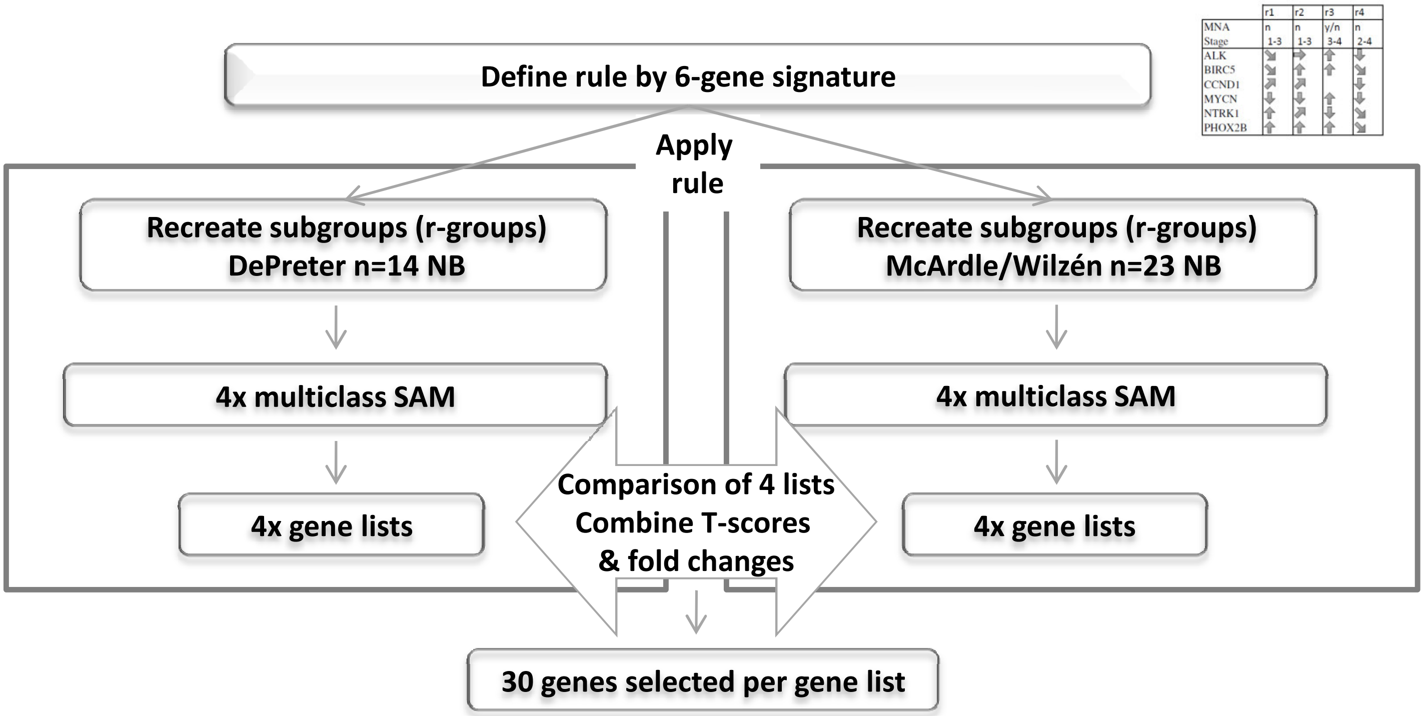

Hierarchical clustering & PCA

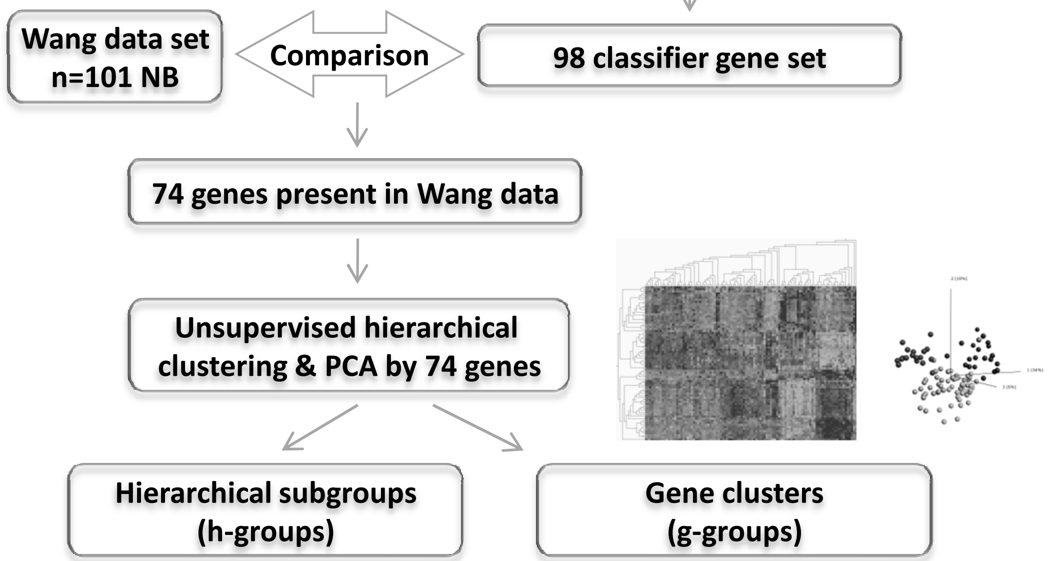

Supplement: Additional file 3 — Workflow of the study. Step 1: Subtype discovery by unsupervised PCA of two data sets (De Preter and McArdle/Wilzén) from three microarray expression studies (upper panel). Data-mining of gene lists from literature, resulting in the selection of 6 NB-associated genes (lower panel). Step 2: Defining the 74-gene subtype discrimination gene set by SAM (upper panel). Verification of subgroup existence by hierarchical clustering and PCA in a third data set (Wang) using the 74-gene set (lower panel). [file 1475-2867-11-9-S3.PDF]
