# Supplementary material for: A 6-gene signature identifies four molecular subgroups of neuroblastoma
Source: Cancer Cell Int. 2011 Apr 14;11:9. doi: 10.1186/1475-2867-11-9 (PMC3095533; doi:10.1186/1475-2867-11-9)
Supplement: Additional file 4 — Gene lists from literature & hits in PubMed. A. List of 15 expression studies used for the data-mining. B. PubMed searches of 157 and 30 genes respectively. PubMed searches were performed as follows: Search 1(left): 157 genes, search term "Gene Symbol"[TIAB] AND "Gene expression"[MeSH Terms] AND "neuroblastoma"[MeSH Terms]. Search 2 (right):30 genes, search term "Gene Symbol"[TIAB] OR "Alias name"[TIAB]) AND "Gene expression"[MeSH Terms] AND "neuroblastoma"[MeSH Terms]. The six NB-associated genes ALK, BIRC5, CCND1, MYCN, NTRK1, and PHOX2B were selected for further analysis (see text for details). [file 1475-2867-11-9-S4.PDF]

A. Gene lists from 15 expression studies

Reference	Reference no.	Genes included in each list
McArdle L et al., 2004	25	31 transcripts most strongly associated with the major genetic subtypes of NB.
Ohira M et al., 2005	19	41 top-ranked genes used for prediction of 2 year and 5 year prognosis of NB (machine learning).
Schramm et al., 2005	26	133 genes: 30 top-ranked genes identified by SAM analysis (MYCN-amplification vs. normal MYCN, High NTRK1 expression vs. low NTRK1 expression, Stage 1/2 vs. stage 4, Stage 4 vs. stage 4s) and the 39 top-ranked genes identified by PAM analysis.
Asgharzadeh et al., 2006	15	55 gene module from a multigene model (PCA).
De Preter K et al., 2006	22	191 genes differentially expressed UF-F and F-UF.
Fischer M, et al., 2006	24	18 genes differentially expressed in stage IVS and IV NB as determined by QPCR.
Oberthuer A et al., 2006	16	144 classifier gene set (PAM).
Wang et al., 2006	28	155 genes: 79 genes mapping to distal 1p with significantly lower expression in both test and validation data sets. 38 genes from unsupervised hierarchical clustering of NB. Top 50 differentially expressed genes in each binary comparison of LOH versus no LOH for chromosome bands 1p36 and 11q23.
Oberthuer A et al., 2007	18	38 top-ranked classifier genes selected by the 10 times repeated 10-fold cross-validation of PAM.
Warnat P et al., 2007		72 differentially expressed genes of advanced stage (3 or 4) tumours without MYCN amplification that show contrasting outcomes (alive or dead) at five years after initial diagnosis.
Albino et al., 2008	21	16 genes differentially expressed in stroma-poor and stroma-rich neuroblastic tumors selected by SAM and Game theory methods.
De Preter et al., 2009	14	132 PAM classifier set
Vermeulen et al., 2009	20	59 genes selected using an innovative data-mining strategy
Thorell et al., 2009	27	89 top-ranked differentially expressed genes from microarray.
Fischer et al., 2010	23	220 genes differentially expressed genes from SAM (NormF vs. NormUF and LOH11qF vs. LOH11qUF with a fold change above 2).

The intersection of the 15 gene lists resulted in 1012 unique genes, out of which 212 genes were present in at least 2 of the 15 gene lists.

B. PubMed searches

Search 1

	Gene name	Hits		Gene name	Hits
1	MYCN	59	80	NEBL	0
2	TH	21	81	NCAM1	0
3	NPY	10	82	NAV3	0
4	DBH	4	83	MTHFD2	0
5	GAL	3	84	MRPL3	0
6	MEIS1	2	85	MMP9	0
7	GAP43	2	86	ME3	0
8	CDC42	2	87	MCM6	0
9	CAMTA1	2	88	MATN2	0
10	TNFRSF25	1	89	MAPT	0
11	S100B	1	90	MAP7	0
12	PTS	1	91	MAP2K4	0
13	PTN	1	92	MAL	0
14	PHOX2B	1	93	MAGEA10	0
15	PCNA	1	94	MAD2L1	0
16	PAM	1	95	LOC284244	0
17	NTRK1	1	96	KIFAP3	0
18	LMO3	1	97	KIF1B	0
19	GNB1	1	98	INPP1	0
20	GATA3	1	99	IL7	0
21	ENO1	1	100	IGL@	0
22	E2F1	1	101	IGHM	0
23	DDX1	1	102	HRK	0
24	CLSTN1	1	103	HMGB2	0
25	CHD5	1	104	HIVEP2	0
26	CD44	1	105	GNAI1	0
27	CCND1	1	106	GMPS	0
28	CADM1	1	107	GFRA3	0
29	BIRC5	1	108	GCH1	0
30	ALK	1	109	GATA2	0
31	WSB1	0	110	GABARAPL1	0
32	ULK2	0	111	FYN	0
33	UBE2C	0	112	FUCA1	0
34	TYMS	0	113	FEZ1	0
35	TWIST1	0	114	FBL	0
36	TRIM28	0	115	EYA1	0
37	TOP2A	0	116	EPS15	0
38	TNFRSF10B	0	117	EPHA5	0
39	TKT	0	118	EPB41L3	0
40	TK1	0	119	ELAVL4	0
41	TFAP2B	0	120	EIF2S1	0
42	SYN3	0	121	EFNB2	0
43	STX12	0	122	E2F3	0
44	SOX4	0	123	DUSP4	0
45	SLC6A8	0	124	DST	0
46	SLC6A2	0	125	DPYSL3	0
47	SLC25A5	0	126	DLK1	0
48	SLC18A1	0	127	DIRAS3	0
49	SH3GL3	0	128	DDC	0
50	SCG2	0	129	CYP1B1	0
51	RRM2	0	130	CTNNBIP1	0
52	RNF11	0	131	CNTNAP2	0
53	RGS7	0	132	CNR1	0
54	PTTG1	0	133	CLNS1A	0
55	PTPRH	0	134	CLCN6	0
56	PTPRF	0	135	CKS2	0
57	PTP4A2	0	136	CENPF	0
58	PTGER3	0	137	CENPA	0
59	PRKCZ	0	138	CDKN3	0
60	PRKACB	0	139	CDC2L2	0
61	PRDM2	0	140	CDC2	0
62	PRAME	0	141	CCNB2	0
63	PMP22	0	142	CCNB1	0
64	PMAIP1	0	143	CBFB	0
65	PLXNC1	0	144	CAMK2B	0
66	PLP1	0	145	CALB1	0
67	PLAT	0	146	BTBD3	0
68	PINK1	0	147	BAI3	0
69	PIK3R1	0	148	ATIC	0
70	PHGDH	0	149	ASCL1	0
71	PDLIM5	0	150	ARHGEF7	0
72	PDE4DIP	0	151	AMIGO2	0
73	PAICS	0	152	ALDH1A2	0
74	PAFAH1B1	0	153	ALCAM	0
75	OLFML2A	0	154	AHCY	0
76	ODZ4	0	155	ADRB2	0
77	ODC1	0	156	ADCY1	0
78	NRCAM	0	157	ABCA8	0
79	NHLH2	0			

Search 2

	Gene name	Aliases	Hits	Selected for expression signature	Comments
1	MYCN	N-myc	151	X	Amplified in 25-35% of NB tumours
2	TH	tyrosine hydroxylase	30		Neuroblastoma marker
3	NTRK1	TrkA	16	X	Differentially expressed between tumour subsets
4	NPY	neuropeptide Y	15		Neuroblastoma marker
5	BIRC5	survivin, IAP4, API4	10	X	Differentially expressed between tumour subsets
6	CAMTA1	calmodulin	9		
7	DBH	dopamine beta-hydroxylase	7		
8	GAL	galanin	6		
9	CCND1	cyclin D1	4	X	Amplified in 3-6% of NB tumours
10	MEIS1	Meis homeobox 1	2		
11	GAP43	PP46, B-50	2		
12	CDC42	G25K	2		
13	PTS	6-pyruvoyltetrahydropterin synthase	2		
14	GNB1	guanine nucleotide binding protein	2		
15	CADM1	IGSF4, TSLC1, cell adhesion molecule 1	2		
16	ALK	anaplastic lymphoma kinase	1	X	Neuroblastoma predisposition gene, mutated in 7% of sporadic cases
17	PHOX2B	paired-like homeobox 2b	1	X	Neuroblastoma predisposition gene, mutated in 2% of sporadic cases
18	CD44	PGP1	1		
19	CHD5	KIAA0444, chromodomain helicase DNA binding protein 5	1		
20	CLSTN1	CDHR12, calsyntenin 1	1		
21	DDX1	DBP-RB, DEAD box polypeptide 1	1		
22	E2F1	RBP3, RBAP-1, RBBP-3	1		
23	ENO1	MPB1, enolase 1	1		
24	GATA3	GATA binding protein 3	1		
25	LMO3	Rhombotin-3, RHOM3, Rhom-3	1		
26	PAM	PAL	1		
27	PCNA	proliferating cell nuclear antigen	1		
28	PTN	pleiotrophin	1		
29	S100B	NEF	1		
30	TNFRSF25	DR3, DDR3, APO-3, APO3	1		
