# Supplementary material for: A 6-gene signature identifies four molecular subgroups of neuroblastoma
Source: Cancer Cell Int. 2011 Apr 14;11:9. doi: 10.1186/1475-2867-11-9 (PMC3095533; doi:10.1186/1475-2867-11-9)
Supplement: Additional file 6 — Rules and assignments of r-groups. Rules for r-group assignments (upper table): Groups (r1-r4) were defined based on the standard deviation (sd) of expression for the six NB-associated genes. R-Assignments of samples from data set 1 and 2 into r-groups (lower table): Expression sd intervals of 5 out of 6 genes had to be in agreement with the rules for each r-group in order to be categorized. [file 1475-2867-11-9-S6.PDF]

Additional file 6. Rules and re-assignment to r-groups.

Rules for r-group discriminations

	ALK	*BIRC5*	*CCND1*	*MYCN*	*NTRK1*	*PHOX2B*
**r1**	x<0,2	x<0	-0,4<x<1,6	x<0,5	**x>0,5**	**x>-0,3**
**r2**	-1<x<0,5	**x>0**	-0,4<x<1,6	x<0,5	**0<x<1,5**	**x>-0,3**
**r3**	**x>0,5**	x>0	-0,4<x<1,6	**x>0**	x<-0,5	**x>-0,3**
**r4**	**x<-0,5**	x<0	x<-0,8	**x<-0,5**	x<0,4	x<0

Groups (r1-r4) were defined based on the expression of 6 genes according to standard deviations from the mean of each gene (sd). Rules with no exceptions are highlighted in bold. The color-scale guides the expression level: red=high expression, orange=elevated expression, yellow=mean expression, light green=decreased expression, green=very low expression.

Results of r-group assignments

De Preter data set

Sample	Study	ALK	BIRC5	CCND1	MYCN	NTRK1	PHOX2B	Sum r-group 1	Sum r-group 2	Sum r-group 3	Sum r-group 4	r-group assignment	Comment
NB1	De Preter	-1,65	-0,36	0,12	-0,22	1,17	0,55	6	4	2	2	**1**	
NB2	De Preter	-1,28	-1,23	0,00	-0,21	1,34	0,41	6	4	2	2	**1**	
NB6	De Preter	-1,12	-1,39	0,71	0,15	1,17	0,85	6	4	3	2	**1**	
NB3	De Preter	-0,83	0,15	0,59	0,19	1,44	0,77	5	6	4	1	**2**	
NB4	De Preter	1,12	0,51	0,59	-0,48	0,88	0,91	4	6	4	0	**2**	
NB10	De Preter	0,49	1,08	0,94	-0,74	-0,24	0,30	3	5	3	2	**2**	
NB13	De Preter	-0,10	0,59	0,88	-0,63	-0,15	0,57	4	5	3	2	**2**	
NB18	De Preter	0,65	1,21	-0,61	0,93	-1,24	0,15	1	3	5	1	**3**	
NB12	De Preter	0,39	1,27	1,28	1,58	-1,28	-0,07	2	4	5	2	**3**	
NB17	De Preter	2,05	0,19	-0,72	1,45	-1,19	-0,03	1	2	5	2	**3**	
NB5	De Preter	0,97	0,73	0,59	1,23	-0,77	-0,31	1	3	5	2	**3**	
NB9	De Preter	1,27	1,38	-0,09	1,56	-1,47	0,42	2	4	6	1	**3**	
NB14	De Preter	-1,02	-0,96	-1,97	-1,78	-1,00	-3,19	3	1	1	6	**4**	
NB15	De Preter	-0,58	-0,96	-2,63	-1,60	0,27	-0,17	4	4	1	6	**4**	
NB8	De Preter	-0,43	-1,12	0,60	-0,43	0,30	-0,67	4	4	1	3	**nd**	
NB16	De Preter	0,43	0,79	-0,26	-0,14	-0,41	0,51	3	5	3	1	**nd**	To low NTRK1 expression for assignment to subgroup r2 (see rule above).
NB7	De Preter	-0,32	-1,49	0,09	-0,45	0,30	-1,56	4	4	1	3	**nd**	

McArdle/Wilzén data set

Sample	Study	ALK	BIRC5	CCND1	MYCN	NTRK1	PHOX2B	Sum r-group 1	Sum r-group 2	Sum r-group 3	Sum r-group 4	r-group assignment	Comment
NB23R4	Wilzén	-1,10	-0,58	0,33	-0,35	0,66	0,15	6	4	2	2	**1**	
18	McArdle	-0,72	-1,03	0,73	-0,13	1,72	0,52	5	4	2	2	**1**	
36	McArdle	-2,07	-0,41	0,80	0,14	1,70	0,06	6	3	3	2	**1**	
27	McArdle	-0,24	-0,27	0,76	-0,09	1,28	0,71	6	5	2	1	**1**	
45	McArdle	-0,13	-0,28	0,11	-0,01	0,66	0,26	6	5	2	1	**1**	
NB15E3	Wilzén	-0,14	-0,96	1,15	-0,08	1,53	0,45	6	4	2	1	**1**	
NB12E8	Wilzén	0,13	-0,36	0,73	-0,10	0,64	0,11	6	5	2	1	**1**	
NB14E6	Wilzén	-0,01	0,43	-0,61	0,03	1,32	0,52	4	5	3	0	**2**	
52	McArdle	0,44	0,76	-0,86	0,19	0,07	0,94	2	5	3	2	**2**	
31	McArdle	0,30	0,58	1,08	-0,37	0,18	0,24	3	6	3	1	**2**	
35	McArdle	-0,51	1,00	0,61	-0,42	-0,21	0,26	4	5	3	2	**2**	
47	McArdle	0,43	0,96	0,13	0,24	0,76	0,53	4	6	4	0	**2**	
48	McArdle	0,02	1,56	0,76	-0,02	0,66	0,61	5	6	3	0	**2**	
67	McArdle	-0,02	0,84	1,06	-0,24	0,06	0,60	4	6	3	1	**2**	
53	McArdle	1,46	1,07	-1,32	4,38	-1,25	0,13	1	3	5	2	**3**	
55	McArdle	0,85	0,72	-0,88	0,83	-1,20	0,21	1	3	5	2	**3**	
86	McArdle	-0,27	1,43	1,51	1,09	-1,13	0,53	3	4	5	1	**3**	
42	McArdle	1,23	-0,25	-0,37	0,69	-1,31	0,13	3	3	5	2	**3**	
NB12R4	Wilzén	0,52	1,48	1,26	0,07	-1,30	0,96	3	5	6	1	**3**	
57	McArdle	-1,89	-1,27	-2,08	-1,13	-1,26	-3,14	2	1	1	6	**4**	
56	McArdle	-0,44	-1,08	-1,20	-1,16	0,06	-0,93	2	3	0	5	**4**	
25	McArdle	-1,09	-1,12	-0,89	-1,39	-1,27	-2,69	2	1	1	6	**4**	
NB15R3	Wilzén	-0,65	-1,03	-1,37	-0,78	-0,24	-0,89	2	2	0	6	**4**	
30	McArdle	-0,57	-1,03	0,07	-0,91	-1,22	0,68	4	4	3	4	**nd**	
54	McArdle	0,29	0,43	-1,06	-0,15	-0,17	0,59	2	4	2	2	**nd**	
59	McArdle	-0,74	-1,12	-0,74	-0,68	0,43	-0,03	3	4	1	4	**nd**	
26	McArdle	0,20	-0,35	1,33	-0,15	0,68	0,41	5	5	2	1	**nd**	Unambiguous results
NB16R4	Wilzén	2,33	-1,13	-0,48	0,35	0,01	-0,75	1	2	2	3	**nd**	
NB10R2	Wilzén	2,37	-1,01	-1,31	0,37	-1,25	-1,89	1	1	3	4	**nd**	
37	McArdle	0,00	2,02	0,73	-0,22	-0,62	0,73	4	5	4	1	**nd**	To low NTRK1 expression for assignment to subgroup r2 (see rule above).

Standard deviations from the mean are presented for each gene. Based on the rules above, the sum of correct expression interval for 6 genes for each r-group (1-4) was calculated. The sums of agreement with rule had to be >5 in order to categorize samples into r-groups. Each samples was categorized according to the highest sum (highlighted). Samples resulting in sums <5 or were not assigned to any group; nd= not determined. Non-assignment could also be due to other facts (see comment);
